# Peptide-conjugated oligonucleotides evoke long-lasting myotonic dystrophy correction in patient-derived cells and mice

**DOI:** 10.1172/JCI128205

**Published:** 2019-09-30

**Authors:** Arnaud F. Klein, Miguel A. Varela, Ludovic Arandel, Ashling Holland, Naira Naouar, Andrey Arzumanov, David Seoane, Lucile Revillod, Guillaume Bassez, Arnaud Ferry, Dominic Jauvin, Genevieve Gourdon, Jack Puymirat, Michael J. Gait, Denis Furling, Matthew J.A. Wood

**Affiliations:** 1 Sorbonne Université, Inserm, Association Institut de Myologie, Centre de Recherche en Myologie, Paris, France.; 2 Department of Physiology, Anatomy and Genetics, University of Oxford, South Parks Road, Oxford, United Kingdom.; 3 Department of Paediatrics, John Radcliffe Hospital, and; 4 MDUK Oxford Neuromuscular Centre, University of Oxford, Oxford, United Kingdom.; 5 Medical Research Council, Laboratory of Molecular Biology, Cambridge, United Kingdom.; 6 Sorbonne Paris Cité, Université Paris Descartes, Paris, France.; 7 Unit of Human Genetics, Hôpital de l’Enfant-Jésus, CHU Research Center, Quebec, Canada.

**Keywords:** Therapeutics, Genetic diseases, Muscle

## Abstract

Antisense oligonucleotides (ASOs) targeting pathologic RNAs have shown promising therapeutic corrections for many genetic diseases including myotonic dystrophy (DM1). Thus, ASO strategies for DM1 can abolish the toxic RNA gain-of-function mechanism caused by nucleus-retained mutant *DMPK* (DM1 protein kinase) transcripts containing CUG expansions (CUGexps). However, systemic use of ASOs for this muscular disease remains challenging due to poor drug distribution to skeletal muscle. To overcome this limitation, we test an arginine-rich Pip6a cell-penetrating peptide and show that Pip6a-conjugated morpholino phosphorodiamidate oligomer (PMO) dramatically enhanced ASO delivery into striated muscles of DM1 mice following systemic administration in comparison with unconjugated PMO and other ASO strategies. Thus, low-dose treatment with Pip6a-PMO-CAG targeting pathologic expansions is sufficient to reverse both splicing defects and myotonia in DM1 mice and normalizes the overall disease transcriptome. Moreover, treated DM1 patient–derived muscle cells showed that Pip6a-PMO-CAG specifically targets mutant *CUGexp-DMPK* transcripts to abrogate the detrimental sequestration of MBNL1 splicing factor by nuclear RNA foci and consequently MBNL1 functional loss, responsible for splicing defects and muscle dysfunction. Our results demonstrate that Pip6a-PMO-CAG induces long-lasting correction with high efficacy of DM1-associated phenotypes at both molecular and functional levels, and strongly support the use of advanced peptide conjugates for systemic corrective therapy in DM1.

## Introduction

Myotonic dystrophy (DM1), one of the most common muscular dystrophies in adults (1), is an RNA-dominant disorder caused by the expression of expanded microsatellite repeats located in the 3′ untranslated region (UTR) of the DM1 protein kinase (*DMPK*) gene (2–4). Mutant transcripts containing an expanded CUG tract are retained within the nucleus as discrete foci (5, 6). Due to their high affinity for CUG expansions (CUGexps), RNA binding factors from the MBNL family are sequestered within the CUGexp-RNA foci, leading to their functional loss (7), which is a central pathophysiologic mechanism in DM1 (8). Thus, altered splicing regulatory activity of MBNL1 in striated muscles results in many alternative splicing misregulations, including those in *CLCN1*, *INSR*, *BIN1*, *DMD*, and *SCN5A* pre-mRNAs that have been associated respectively with myotonia, insulin resistance, muscle weakness, dystrophic process, and cardiac conduction defects, all symptoms of DM1 (9–14).

Given that the toxic RNA gain-of-function mechanism in DM1 is driven by a pathologic CUGexp tract within *DMPK* mRNAs, therapeutic approaches using modified antisense oligonucleotides (ASOs) aimed at either degrading CUGexp transcripts using RNase H–active ASOs (15–17) or releasing sequestered MBNL1 from CUGexp-containing RNAs using RNase H–inactive CAG*n* ASOs (1–20) have demonstrated efficient reversal of DM1-associated phenotypes. However, unlike other muscle diseases like Duchenne muscular dystrophy (DMD), the integrity of the skeletal muscle fiber membrane is not compromised in DM1, resulting in poor penetration of naked phosphorodiamidate oligomer–ASOs (PMO-ASOs) or 2′-O-methyl–ASOs (21). Backbone modifications led to the design of more potent chemistries, including 2′-O-methoxyethyl or 2′-4′–constrained ethyl–modified residues that enhanced ASO delivery and consequently therapeutic efficacy in skeletal muscles of DM1 mice (15, 17). This approach has been tested in a human clinical trial (NCT02312011); however, systemic administration of the selected naked ASO did not achieve sufficient concentrations in skeletal muscle of patients to have marked clinical benefits. Therefore, tissue uptake remains a major challenge for ASO therapies in DM1.

Among strategies to improve delivery of ASOs in DM1 skeletal muscles, cell-penetrating peptide conjugates offer an attractive avenue (22–24). Indeed, systemic administration of advanced Pip6a peptides conjugated with splice-switch oligonucleotides has shown promising results in DMD mice by restoring a truncated but functional dystrophin (25). Pip6a is a cell-penetrating peptide composed of a hydrophobic core region flanked on each side by arginine-rich domains containing β-alanine and aminohexanoyl spacers. This peptide sequence has the ability to deliver associated cargoes across the plasma and endosomal membranes and is stable to serum proteolysis (25). Here, we have investigated the potential of Pip6a conjugates to enhance RNase H–inactive CAG*n*-PMO delivery in skeletal muscles of DM1 mice in order to target nucleus-retained mutant-CUGexp transcripts and inhibit toxicity associated with expanded repeats.

## Results and Discussion

To test whether advanced Pip6a conjugates enhanced ASO delivery and activity in skeletal muscles of DM1 mice following systemic administration, antisense PMOs composed of a CAG7 sequence, either naked (PMO) or conjugated to Pip6a peptide (Pip6a-PMO), were injected into the tail vein of HSA-LR mice. This well-characterized DM1 mouse model expresses 250 CTG repeats in the 3′ UTR of the human skeletal muscle α actin (*HSA*) gene and displays molecular as well as functional DM1-associated abnormalities (26). The skeletal muscle–specific expression of CUGexp RNAs in HSA-LR mice leads to the formation of ribonuclear foci that sequester MBNL1 proteins, resulting in splicing defects and myotonia, as found in DM1 patients (11, 27).

**Figure 1:**
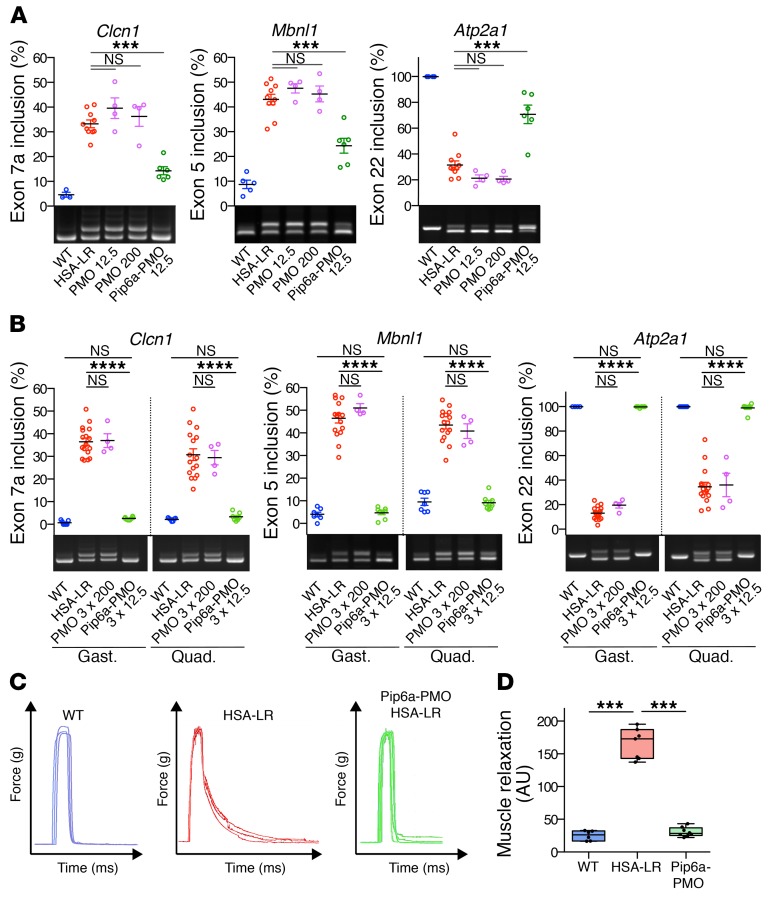
Pip6a-PMO corrects molecular and functional defects in HSA-LR mice. HSA-LR mice were injected with single or multiple doses of naked PMO-CAG7 (PMO) or Pip6a-PMO-CAG7 (Pip6a-PMO) by intravenous injection and analyzed 2 weeks after treatment. (**A**) Quantification of splicing correction induced by a single 12.5 mg/kg dose of Pip6a-PMO, or 12.5 or 200 mg/kg dose of PMO in gastrocnemius of treated mice (WT *n* = 4; HSA-LR *n* = 10; PMO *n* = 4; Pip6a-PMO *n* = 6). (**B**) Representative RT-PCR results and quantification of alternative splicing profiles in gastrocnemius (gast.) and quadriceps (quad.) of HSA-LR mice treated with 3 injections of PMO at 200 mg/kg or Pip6a-PMO at 12.5 mg/kg (WT *n* = 7; HSA-LR *n* = 16; PMO *n* = 4; Pip6a-PMO *n* = 8). (**C**) Representative maximal force/time curves obtained by in situ force measurements of Pip6a-PMO–treated mice (3 times 12.5 mg/kg) compared with WT and HSA-LR mice. (**D**) Correction of myotonia (measured as the area under the force/time curve during relaxation after maximal muscle contraction) in Pip6a-PMO–injected HSA-LR mice (WT *n* = 6; HSA-LR *n* = 7; Pip6a-PMO *n* = 8). Data are expressed as mean ± SEM, except for **D** (boxes, 25th–75th percentile; whiskers, min to max; black line, the mean). ***P < 0.001; ****P < 0.0001 by 1-way ANOVA with Newman-Keuls post hoc test. NS, not significant.

Two weeks after a single injection of a dose of 12.5 mg/kg, mice were sacrificed and the correction of DM1-splicing defects was used as a biomarker of ASO activity. Pip6a-PMO induced a partial but significant correction of several MBNL1-dependent splicing defects (e.g., *Clcn1* exon 7a, *Mbnl1* exon 5, and *Atp2a1* exon 22) in HSA-LR gastrocnemius muscle, whereas no effect was observed with naked PMO at the same dose or even at a much higher dose of 200 mg/kg (Figure 1A). Moreover, 2 and 3 systemic injections of Pip6a-PMO (12.5 mg/kg) led to an almost complete normalization and a full correction, respectively, of DM1-splicing profiles of *Clcn1*, *Mbnl1*, and *Atp2a1* genes in both HSA-LR gastrocnemius and quadriceps muscles (Supplemental Figure 1 and Figure 1B). In contrast, even 3 injections of naked PMO at a high dose of 200 mg/kg did not improve splicing defects. PMO concentration was also quantified in skeletal muscle of HSA-LR mice by a custom ELISA. Results showed PMO concentrations of greater than 1 nM 2 weeks after Pip6a-PMO treatment, whereas mice treated with naked PMO showed low picomolar concentrations, confirming the poor uptake of naked PMO in non–renal tissue (Supplemental Figure 2). Interestingly, systemic administration of Pip6a-PMO in DM1 mice also leads to therapeutic levels of PMO-CAG in heart and diaphragm tissues that are affected in DM1 disease. Despite the fact that restriction of CUGexp expression to the *hACTA1* gene in HSA-LR mice does not allow the assessment of ASO therapy in cardiac tissue, our results strongly suggest that the efficient biodistribution of Pip6a-conjugated PMO into striated muscles could counteract other DM1-related symptoms, including cardiac-conduction defects and respiratory failure, which are the most common causes of death in DM1 patients.

To determine whether Pip6a-PMO treatment can improve muscle function, we evaluated myotonia, a DM1 hallmark characterized by delayed muscle relaxation also detectable in HSA-LR mice. As shown in Figure 1, C and D, Pip6a-PMO treatment completely abolished myotonia in gastrocnemius muscles of HSA-LR mice, which is in agreement with the correction of *Clcn1* exon 7a missplicing responsible for myotonia (28). In contrast, systemic injections of a control Pip6a-PMO composed of a reverse GAC7 sequence (Pip6a-Ctrl) had no effect on either DM1-splicing defects or myotonia in HSA-LR mice (Supplemental Figure 3, A and B). Altogether, these results showed that a low-dose treatment of Pip6a conjugates allows efficient systemic delivery of ASO in skeletal muscle of DM1 mice. Remarkably, optimal beneficial effects at both molecular and functional levels were achieved with a cumulative dose of Pip6a-ASO that is 5 to 10 times lower than either unconjugated or conjugated ASOs previously described and evaluated in the same DM1 mouse model (15, 17, 22, 29).

**Figure 2:**
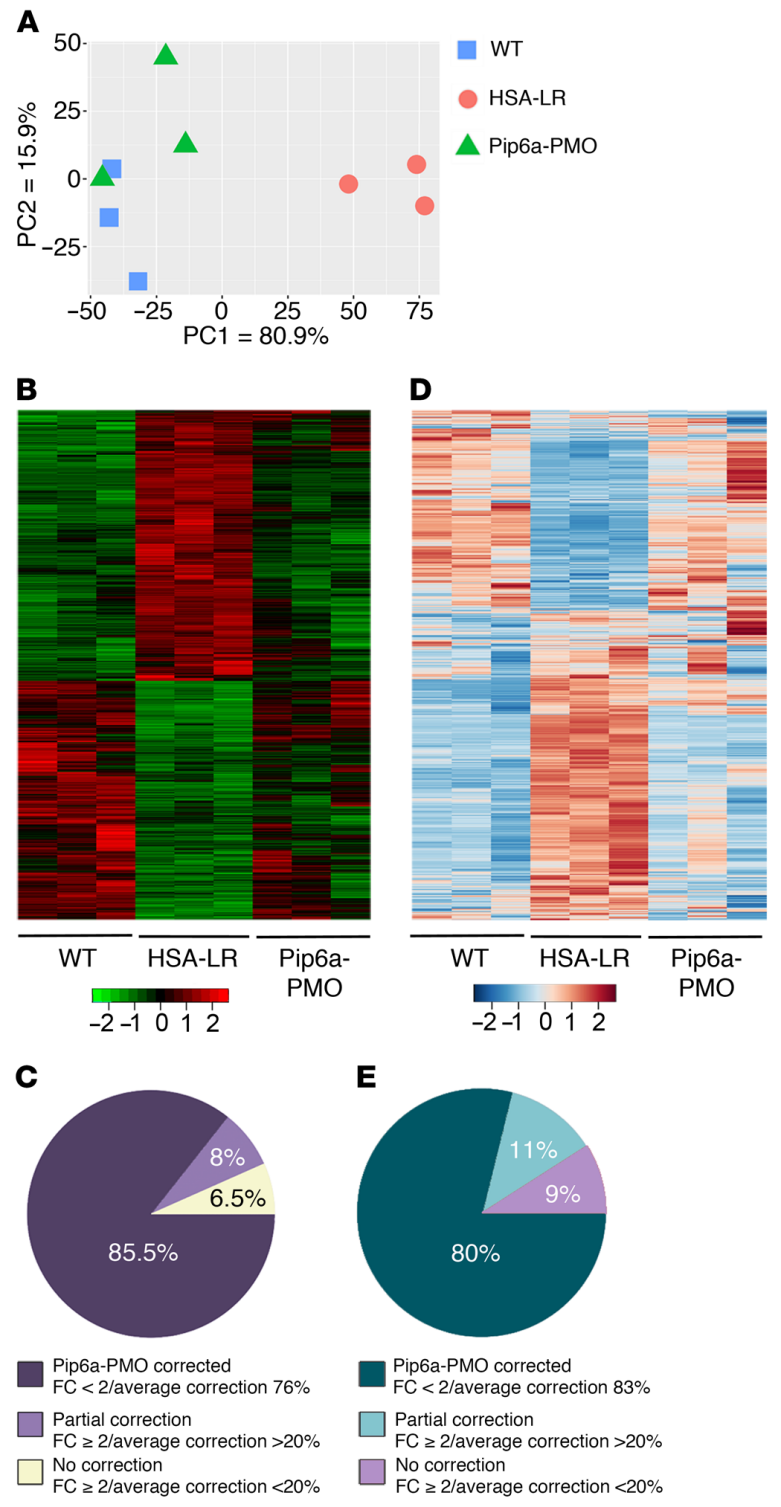
Treatment with Pip6a-PMO normalizes global transcriptome at both expression and splicing levels. Transcriptomic analysis by RNA sequencing was performed on total RNA isolated from gastrocnemius muscles of treated HSA-LR mice compared with HSA-LR and WT mice (*n* = 3). (**A** and **B**) Principal component analysis (**A**) and heatmap graphic (**B**) of all significantly expressed transcripts (adj. *P* < 0.1) reveal a global correction of the gene expression profile with Pip6a-PMO treatment. (**C**) Global correction of gene expression by Pip6a-PMO treatment (*n* = 376; FC ≥ 2, adj. *P* < 0.1): 85.5% of transcripts return to FC < 2 with an average correction index of 76%; 8% of transcripts remain at FC ≥ 2 but with correction index > 20%; 6.5% of transcripts are not corrected. (**D**) Heatmap graphic of all significant deregulated exon_bin (normalized counts) reveals a global correction of missplicing events with Pip6a-PMO treatment. (**E**) Overall correction of alternative splicing profiles by Pip6a-PMO treatment (*n* = 339 splicing events; FC ≥ 2, adj. *P* < 0.1): 80% of events return to FC < 2 with an average correction index of 83%; 11% remain at FC ≥ 2 but with correction index > 20%; 8% are not corrected.

To examine the overall effect of Pip6a-PMO on the skeletal muscle transcriptome of DM1 mice, we performed deep, paired-end RNA sequencing. Principal component analysis (PCA) showed that gene expression in the gastrocnemius muscle of HSA-LR mice treated with Pip6a-PMO was shifted toward that of WT mice (Figure 2A). Moreover, the heatmap generated with all the transcripts having a significant change (*n* = 3176; adj. *P* < 0.1; no threshold in fold change [FC]) confirmed the global gene expression correction in treated HSA-LR mice (Figure 2B). Strikingly, the vast majority (85%) of the most significant changes observed at the gene expression level in HSA-LR mice (FC ≥ 2 and adj. *P* < 0.1; 376 transcripts) were corrected in treated mice with an average correction index of 76% (Figure 2C and Supplemental Table 1). In addition, most of the deregulated genes containing 7 or more CTG repeats in skeletal muscles were also corrected in treated HSA-LR mice (Supplemental Table 2). Transcriptomic analysis also confirmed that Pip6a-PMO treatment induced a global correction of alternative splicing misregulation measured in HSA-LR mice (adj. *P* < 0.1; no threshold in FC; Figure 2D). Thus, 80% of the most deregulated splicing events in HSA-LR mice (FC ≥ 2 and adj. *P* < 0.1; 339 events) were normalized in treated mice an average correction index of 83% (Figure 2E), also leading to correction of the deregulated biological pathways (Supplemental Figure 4 and Supplemental Table 3). Similar results were obtained from the quadriceps muscle of the same treated mice (Supplemental Figure 5), indicating that the muscle transcriptome of DM1 mice could be almost normalized by Pip6a-PMO treatment targeting pathologic CUGexps. In addition, PCA and heatmap analysis (FC ≥ 1.5; *P* < 0.05; *n* = 118) of the proteome assessed by label-free mass spectrometry revealed that protein expression in the quadriceps muscle of treated HSA-LR mice also tends to shift from a disease toward a WT profile, supporting the correction of the DM1 muscle phenotype by Pip6a-PMO treatment (Supplemental Figure 6 and Supplemental Table 4).

**Figure 3:**
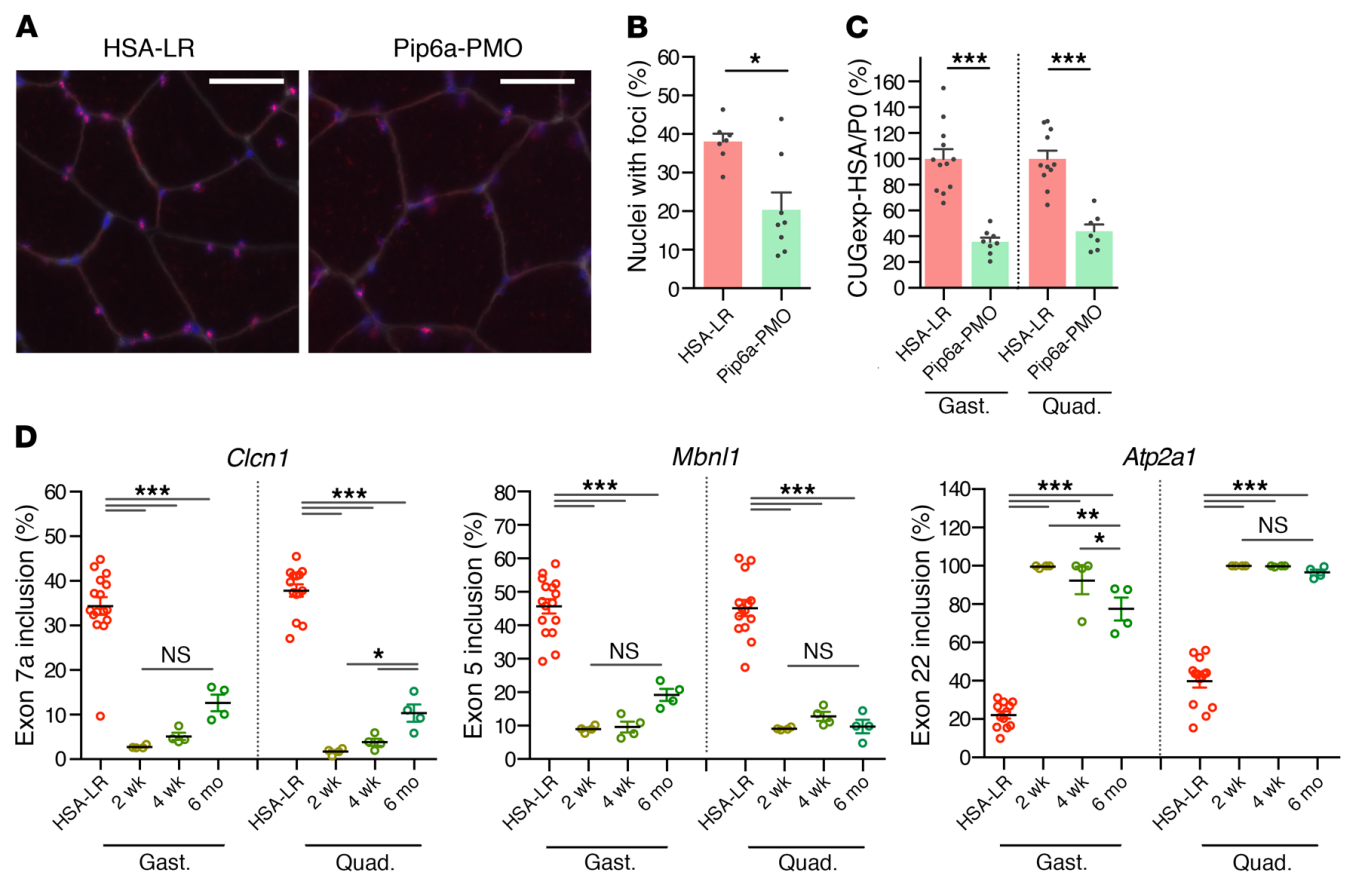
Treatment with Pip6a-PMO normalizes DM1-specific phenotype. HSA-LR mice were injected in the tail vein with three 12.5 mg/kg doses of Pip6a-PMO and analyzed 2 weeks after treatment. (**A**) Representative images of gastrocnemius muscle section stained for CUGexp foci (FISH, red), fiber membranes (WGA, gray), and nuclei (Hoechst, blue). Scale bars: 50 μm. (**B**) Quantification of the number of nuclei with foci in gastrocnemius muscle sections (HSA-LR *n* = 7; Pip6a-PMO *n* = 8). (**C**) Quantification of HSA transcripts levels normalized to murine Rlp0 (P0) by qRT-PCR (HSA-LR *n* = 12; Pip6a-PMO *n* = 8). (**D**) Quantification of splicing correction in gastrocnemius (gast.) and quadriceps (quad.) at 2 weeks, 4 weeks, and 6 months after treatment (HSA-LR *n* = 16; *n* = 4 for each time point of Pip6a-PMO). Data are expressed as mean ± SEM. *P < 0.05; **P < 0.01; ***P < 0.001 by Mann-Whitney test (**B**) or 1-way ANOVA with Newman-Keuls post hoc test (**C** and **D**). NS, not significant.

To further determine the consequences of Pip6a-PMO for CUGexp-RNA behavior, we examined nuclear RNA foci number and CUGexp-RNA levels since it has been shown previously that both features were reduced by RNase H–inactive CAG*n* ASOs (18, 19). Likewise, a similar mechanism of action, which is not yet fully explained, was also observed in Pip6a-PMO–treated HSA-LR mice, as (i) missplicing reversal due to the release of functional MBNL1 from CUGexp-RNA foci was associated with a 50% reduction in the number of myonuclei containing RNA foci in treated muscles (Figure 3, A and B, and Supplemental Figure 7); and (ii) a 60% decrease of CUGexp-RNA steady-state levels was detected following Pip6a-PMO treatment, whereas Pip6a-Ctrl treatment had no effect on CUGexp-RNA expression (Figure 3C and Supplemental Figure 3C).

In addition, the duration of Pip6a-PMO action in HSA-LR skeletal muscles was evaluated because the activity and beneficial effect of nuclease-resistant ASOs could be maintained for several weeks in vivo (15, 30–32). Correction of splicing changes was used as a molecular biomarker and the Pip6a-PMO effect was assessed up to 6 months after treatment. As observed in Figure 3D, splicing misregulation of *Clcn1* exon 7a, *Mbnl1* exon 5, and *Atp2a1* exon 22 pre-mRNAs was completely normalized in both HSA-LR gastrocnemius and quadriceps muscles until 4 weeks after systemic administration. Moreover, a significant 80% to 100% of correction of these splicing defects was still measured 26 weeks after treatment, supporting a long-lasting activity of Pip6a-PMO, which remained nearly complete for a 6-month period. Furthermore, mild histological changes observed in liver or kidney 2 weeks after Pip6a-PMO injections were mostly reversed 6 months later (Supplemental Table 5), supporting transient and reversible side effects of Pip6a-PMO treatment.

**Figure 4:**
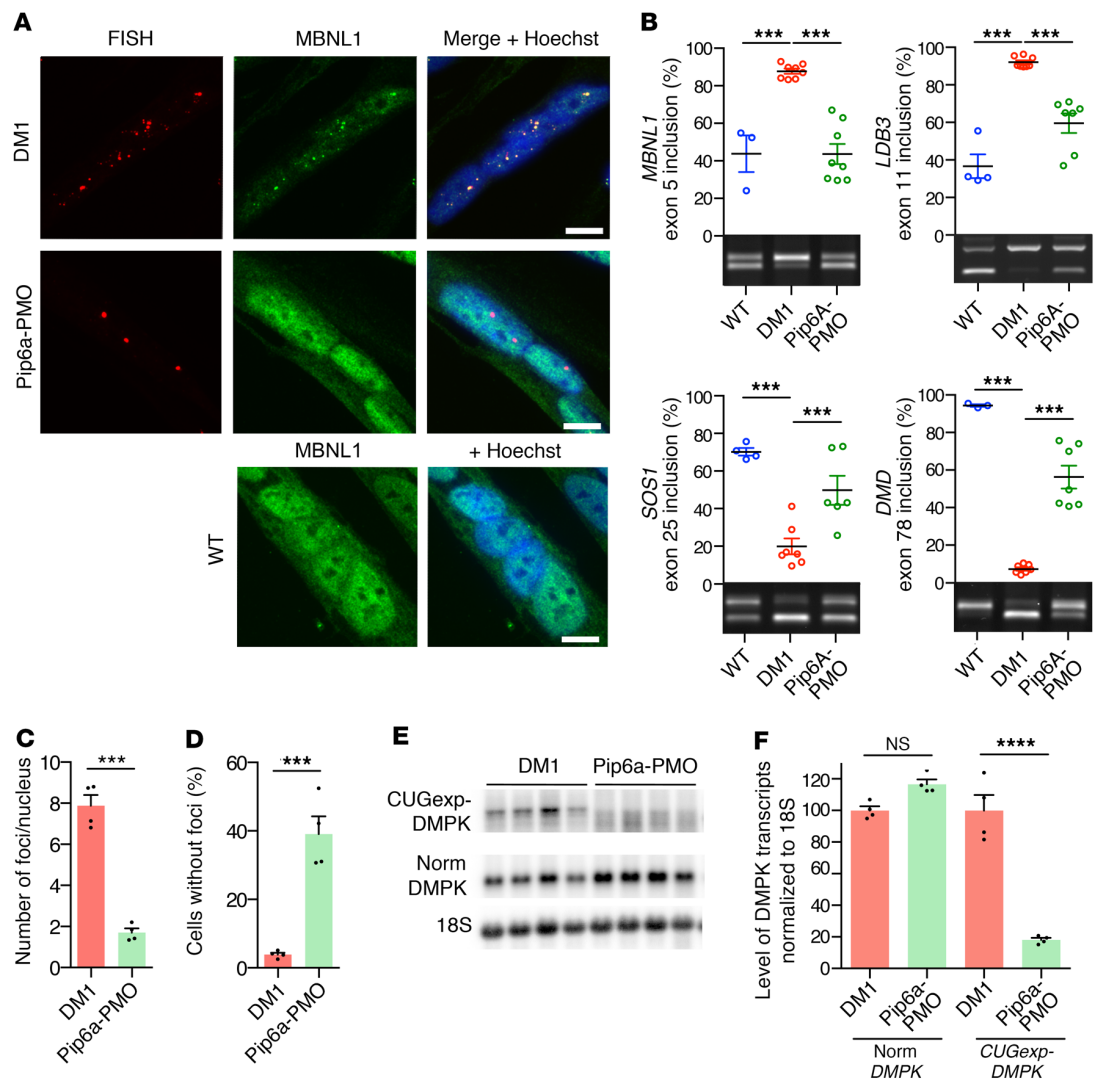
Pip6a-PMO corrects DM1-specific molecular symptoms in DM1 muscle cells. Four-day-differentiated immortalized DM1 myoblasts (2600 CTG repeats) were treated with 1 μM Pip6a-PMO and analyzed after 24 hours. (**A**) Combined FISH (Cy3-CAG, red) and immunofluorescence (MBNL1, green) on DM1 or WT differentiated myoblasts. Scale bars: 10 μm. (**B**) Quantification of splicing corrections by RT-PCR (WT *n* = 4; DM1 and Pip6a-PMO *n* = 7). (**C**) Quantification of mean number of foci per nucleus in treated DM1 differentiated myoblasts (*n* = 4; >500 nuclei per *n*). (**D**) Quantification of the number of nuclei without foci in treated DM1 differentiated myoblasts (*n* = 4; >500 nuclei per *n*). (**E** and **F**) Levels of mutant *DMPK* and normal *DMPK* transcripts analyzed by Northern blot using a *DMPK* probe (*n* = 4). Data are expressed as mean ± SEM. ***P < 0.001; ****P < 0.0001 by 1-way ANOVA with Newman-Keuls post hoc test (**B** and **F**) or Mann-Whitney test (**C** and **D**). NS, not significant.

Lastly, molecular effects of Pip6a-PMO were assessed in human DM1 muscle cells carrying a large expansion (2600 repeats) and expressing within its natural context both mutant *CUGexp-DMPK* and nonmutated normal *DMPK* transcripts (33). Pip6a-PMO treatment induced the displacement and relocation of MBNL1 from RNA foci toward the nucleoplasm as observed in WT muscle cells (Figure 4A). Moreover, MBNL1-dependent splicing defects of *LDB3*, *MBNL1*, *SOS1*, and *DMD* pre-mRNAs that are present in DM1 differentiated muscle cells were significantly corrected by Pip6a-PMO, confirming the functional restoration of MBNL1 (Figure 4B). Similar results were obtained in another DM1 cell line carrying 1300 CTG and as described previously (33) (Supplemental Figure 8). Importantly, no splicing changes were observed in DM1 muscle cells treated with Pip6a-Ctrl or naked PMO nor in WT muscle cells treated with Pip6a-PMO (Supplemental Figure 9). As a consequence of MBNL1 displacement from CUGexp-RNA foci, Pip6a-PMO treatment induced a 79% decrease in the number of foci per nucleus and was associated with a 40% increase in the number of nuclei without foci (Figure 4, C and D), which was consistent with a 77% decrease of *CUGexp*-*DMPK* transcript levels in treated DM1 cells (Figure 4, E and F). A direct measurement of *CUGexp*-*DMPK* transcripts using single-molecule RNA FISH also confirmed the reduced level of *CUGexp-DMPK* mRNA by Pip6a-PMO treatment in muscle cells expressing the human *DMPK* gene with 1500 CTG (Supplemental Figure 10). Remarkably, products of nonmutated *DMPK* alleles were not affected in DM1-treated cells, supporting a specific targeting of Pip6a-PMO composed of a CAG7 sequence to mutant *DMPK* transcripts containing a CUGexp tract (Figure 4, E and F).

In conclusion, our study demonstrates that Pip6a cell-penetrating peptide improves the penetration of ASOs in striated muscles of DM1 mice after systemic delivery. Thus, low-dose treatment with a Pip6a-conjugated PMO directed against pathogenic CUGexp repeats is sufficient to achieve an effective concentration of ASOs in muscle fibers and induces an efficient and long-lasting correction of molecular and functional phenotypes in DM1 mice. Altogether, these results strongly support the development of ASO-conjugate peptides for further clinical trial in DM1 as well as other microsatellite expansion disorders.

## Methods

See the supplemental methods for a full description of all experimental procedures as well as complete unedited blots.

### Statistics.

All group data are expressed as mean ± SEM. Comparisons were performed by Mann-Whitney test or 1-way ANOVA followed by Newman-Keuls or Tukey’s post hoc test using Prism 6 software (GraphPad Software, Inc.). Differences between groups were considered significant when *P* ≤ 0.05.

### Study approval.

Experiments on HSA-LR mice were carried out according to French legislation and Ethics committee approval (number 1760-2015091512001083v6).

### Data availability.

Complete raw data generated from RNA sequencing were deposited in the NCBI’s Gene Expression Omnibus database (GEO GSE134926).

## Author contributions

Experiments were performed by AFK, MAV, LA, AH, DS, AF, LR, and DJ. AA produced the Pip6a-PMO. Transcriptomic analysis was performed by NN. Proteomics data were generated and analyzed by AH. Data were analyzed by AFK, MAV, GB, GG, JP, MJG, MJAW, and DF. The study was designed and written by MJAW and DF. As part of a joint collaborative work, co–first and co–last authors order was decided to ensure both teams have equitable representation.

## Supplementary Material

Supplemental data

## Version 1 09/03/2019

In-Press Preview

## Version 2 09/30/2019

Electronic publication

## Version 3 11/01/2019

Print issue publication
