# Supplementary material for: Peptide-conjugated oligonucleotides evoke long-lasting myotonic dystrophy correction in patient-derived cells and mice
Source: J Clin Invest. 2019 Sep 30;129(11):4739–44. doi: 10.1172/JCI128205 (PMC6819114; doi:10.1172/JCI128205)
Supplement: Supplemental data [file jci-129-128205-s147.pdf]

Supplementary material

Peptide-conjugated oligonucleotides evoke long-lasting myotonic dystrophy correction in patient-derived cells and mice

Arnaud F. Klein[1†], Miguel A. Varela[2,3,4†], Ludovic Arandel[1], Ashling Holland[2,3,4], Naira Naouar[1], Andrey Arzumanov[2,5], David Seoane[2,3,4], Lucile Revillod[1], Guillaume Bassez[1], Arnaud Ferry[1,6], Dominic Jauvin[7], Genevieve Gourdon[1], Jack Puymirat[7], Michael J. Gait[5], Denis Furling[1#*] & Matthew J. A. Wood[2,3,4#*]

[1]Sorbonne Université, Inserm, Association Institut de Myologie, Centre de Recherche en Myologie, CRM, F-75013 Paris, France

[2]Department of Physiology, Anatomy and Genetics, University of Oxford, South Parks Road, Oxford, UK

[3]Department of Paediatrics, John Radcliffe Hospital, University of Oxford, Oxford, UK

[4]MDUK Oxford Neuromuscular Centre, University of Oxford, Oxford, UK

[5]Medical Research Council, Laboratory of Molecular Biology, Francis Crick Avenue, Cambridge, UK

[6]Sorbonne Paris Cité, Université Paris Descartes, F-75005 Paris, France

[7]Unit of Human Genetics, Hôpital de l'Enfant-Jésus, CHU Research Center, QC, Canada

† These authors contributed equally to the work

# These authors shared co-last authorship

Methods

Synthesis of Peptide-PMO Conjugates. Pip6a Ac-(RXRRBRRXRYQFLIRXRBRXRB)-CO OH was synthesized and conjugated to PMO as described previously (1). The PMO sequence targeting CUG expanded repeats (5′-CAGCAGCAGCAGCAGCAGCAG-3′) and PMO control reverse (5′-GACGACGACGACGACGACGAC-3′) were purchased from Gene Tools LLC.

Animal model and ASO injections. Experiments were carried out in the "Centre d'études fonctionnelles" (Faculté de Médecine Sorbonne University) according to French legislation and Ethics committee approval (#1760-2015091512001083v6). HSA-LR mice are gift from Pr. Thornton. The intravenous injections were performed by single or multiple administrations via the tail vein in mice of 5 to 8 weeks of age. Doses of 12.5 mg/kg of Pip6a-PMO-CAG7 and 12.5 or 200 mg/kg of PMO were diluted in 0.9% saline and given at a volume of 5-6 µL/g of body weight. Multiple injections were done at 2 weeks apart. Myotonia was evaluated and tissues were harvested 2 weeks after the last injection. For long-term experiments, tissues were harvested 2, 4 weeks or 6 months after the last injection.

In situ myotonia / muscle relaxation measurement. The isometric contractile properties of gastrocnemius muscle were studied in situ as previously described (2). Mice were anesthetized with a solution of ketamine/xylasine (80 mg/kg and 15 mg/kg, respectively). The knee and foot were fixed with clamps and pins. The distal tendon of the gastrocnemius muscle was attached to a lever arm of a servomotor system (305B, Dual-Mode Lever). Data were recorded and analyzed using PowerLab system (4SP, ADInstruments) and software (Chart 4, ADInstruments). The sciatic nerve (proximally crushed) was stimulated by a bipolar silver electrode using a supramaximal (10-V) square wave pulse of 0.1 ms duration. Absolute maximal isometric tetanic force (P0) was measured during isometric contractions in response to electrical stimulation (frequency of 25 to 150 Hz, train of stimulation of 500 ms). Myotonia was measured as the delay of relaxation muscle after the measure of P0.

Cell culture and Pip6a-PMO treatment. Immortalized myoblasts from healthy individual or DM1 patient with 2600 CTG repeats and immortalized MYOD1-inducible DM1 fibroblasts with 1300CTG repeats were previously described (3). Immortalized murine DMSXL MYOD1-inducible fibroblasts with 1500 CTG repeats in the 3'UTR of the human *DMPK* gene are derived from DMSXL mice (4) and were modified as previously described (5). Immortalized myoblasts were cultivated in a growth medium consisting of a mix of M199:DMEM (1:4 ratio; Life technologies) supplemented with 20% FBS (Life technologies), 50 µg/ml gentamycin (Life technologies), 25 µg/ml fetuin, 0.5 ng/ml bFGF, 5 ng/ml EGF and 0.2 µg/ml dexamethasone (Sigma-Aldrich). Fibroblasts are cultivated in a DMEM growth medium supplemented with 15% FBS and 50 µg/mL gentamycin. Myogenic differentiation was induced by switching confluent cell cultures to DMEM medium supplemented with 5 µg/ml insulin (Sigma-Aldrich) for myoblasts and a additional 4 μg/mL of doxycycline (Sigma-Aldrich) for the trans-differentiation of immortalized MYOD1-inducible fibroblasts. For treatment, WT or DM1 cells are differentiated for 4 days. Then, medium was changed with fresh differentiation medium with pip6a-PMO at a 1 µM concentration. Cells were harvested for analysis 24h after

treatment.

RNA isolation, RT-PCR and qPCR analysis. For mice tissues: prior to RNA extraction, muscles were disrupted in TriReagent (Sigma-Aldrich) using Fastprep system and Lysing Matrix D tubes (MP biomedicals). For human cells: prior to RNA extraction, cells were lysed in a proteinase K buffer (500 mM NaCl, 10 mM Tris-HCl, pH 7.2, 1.5 mM $MgCl_2$, 10 mM EDTA, 2% SDS and 0.5 mg/ml of proteinase K) for 45 min at 55°C. Total RNAs were isolated using TriReagent according to the manufacturer's protocol. One microgram of RNA was reverse transcribed using M-MLV first-strand synthesis system (Life Technologies) according to the manufacturer's instructions in a total of 20 µL. One microliter of cDNA preparation was subsequently used in a semi-quantitative PCR analysis according to standard protocol (ReddyMix, Thermo Scientific). PCR amplification was carried out for 25-35 cycles within the linear range of amplification for each gene. PCR products were resolved on 1.5-2% agarose gels, ethidium bromide-stained and quantified with ImageJ software. The ratios of exon inclusion were quantified as a percentage of inclusion relative to total intensity of isoform signals. To quantify the mRNA expression, real-time PCR was performed using a Lightcycler 480 (Roche). Reactions were performed with SYBR Green kit (Roche) and according to the manufacturer's instructions. PCR cycles were a 15-min denaturation step followed by 50 cycles with a 94°C denaturation for 15 s, 58°C annealing for 20 s and 72°C extension for 20 s. Mouse Rrlp0 mRNA were used as standard. Data were analyzed with the Lightcycler 480 analysis software. Primers are identified in **Supplementary table 6**.

Fluorescent in situ hybridization / immunofluorescence. Fluorescent in situ hybridization (FISH) experiments were done as previously described (6) using a Cy3-labeled 2′OMe (CAG)7 probe (Eurogentec). For combined FISH-Immunofluorescence experiments, immunofluorescence staining was done after FISH last washing as described previously (7) with a rabbit polyclonal anti-MBNL1 antibody (gift from C. Thornton ; 1:2000) followed by a secondary Alexa Fluor 488-conjugated goat anti-rabbit (1:500, Life technologies) antibody. Single molecule FISH (smFISH) was performed as previously described (8). Briefly cells are incubated with a mix a 24 human *DMPK*-specific DMA probes which are hybridized to a Cy3-labelled universal FLAP oligonucleotide (**Supplementary table 6**). Pictures were captured using an Olympus BX60 microscope and Metamorph software (Molecular Devices). Confocal images were taken with a Nikon Ti2 microscope equipped with a motorized stage and a Yokogawa CSU-W1 spinning disk head coupled with a Prime 95 sCMOS camera (Photometrics). Images were processed with Adobe Photoshop software. Automated counting of nuclei with foci, number of foci per nuclei and/or intensity of FISH signal was done using Fiji software and custom scripts.

RNA sequencing. Total RNA from gastrocnemius and quadriceps muscles of WT, HSA-LR and Pip6a-PMO-CAG7 treated mice were sent to the Wellcome Trust Centre for Human Genetics Sequencing Facility. After complete RNA quality control on each sample (quantification in duplicate and RNA6000 Nano LabChip analysis on Bioanalyzer from Agilent), libraries were generated from each sample using TruSeq Stranded mRNA Library Prep Kit (Illumina), where mRNA are isolated by polyA selection, and pooled together by

multiplexing them using barcoded adapters. The pooled libraries were then sequenced on Illumina's HiSeq4000 sequencing system to obtain 150bp paired end reads. Sequence alignment was provided by the Sequencing Facility where the 150bp paired end reads were trimmed and mapped on *Mus musculus* mm10 reference genome using HISAT software (9). Then, all bioinformatics and statistical analysis were performed using custom scripts. Differentially expressed splicing events (exon_bin) between WT, HSA-LR and Pip6a-PMO-CAG7 samples were identified using DEXSeq software(10) by (i) identifying all possible exon_bins from mm10 Gencode annotation (M12 release) and estimating exon_bin counts for each sample with the provided python scripts (ii) normalization and (iii) differential exon usage tests to quantify log2 fold changes between the different conditions and their associated p-values. Differential gene expression was computed using DESeq2 software (11) from gene counts generated by FeatureCounts tool (12). Differentially expressed exon_bins or genes were assessed when the log2 fold change was greater than 2 and the adjusted p-value for multiple comparison above 0.1.

Sample preparation for proteomic analysis. Protein was extracted from WT, HSA-LR untreated and HSA-LR Pip6a-PMO treated (12.5 mg/kg, 3 administrations at 2 weeks intervals) from quadriceps muscle. Tissue was homogenised in lysis buffer (7 M urea, 2 M thiourea, 65 mM CHAPS, 100 mM DTT and supplemented with protease and phosphatase inhibitors) by bead extraction and incubated for 2.5 hours at 4°C. The crude extract was clarified by centrifugation at 16,000*xg* for 20 min. Protein samples were processed using a ReadyPrep 2-D clean up kit (BioRad) and resuspended in 6 M urea, 2 M thiourea (in 10 mM Tris-HCl, pH 8.0). Protein concentration was determined by BCA. Equalised tissue lysates containing 25 µg protein were prepared for label-free mass spectrometry analysis, as described previously (13). Protein lysates were reduced with 10 mM DTT for 30 min at 37°C and alkylated with 55 mM iodoacetamide for 30 min at room temperature in the dark. Samples were diluted with four volumes 50 mM ammonium bicarbonate and digested with 1 µg trypsin and incubated at 37°C overnight. Digestion was terminated by acidification with 2% trifluoroacetic acid (TFA) in 20% acetonitrile (3:1 (v/v) dilution). Peptides were purified using C18 spin columns (Thermo Fisher), lyophilised and stored at -20°C until mass spectrometry analysis.

Liquid chromatography-mass spectrometry analysis. Peptides were analysed by LC-MS/MS using a Dionex Ultimate 3000 nanoUPLC and a QExactive HF mass spectrometer (Thermo Fisher). Peptides were analysed by LC-MS/MS using a Dionex Ultimate 3000 NanoUPLC system via a nano electrospray source coupled to a QExactive HF mass spectrometer (Thermo Fisher). A 500 mm x 75 µm C18 (2 µm particle size) EASY-Spray column was used to separate peptides over a 60 minute gradient of 2 – 35 % acetonitrile in 5 % DMSO, 0.1 % formic acid with a flow rate of 250 nL/min. The mass spectrometer was operated in data-dependent and positive mode. MS1 scans were acquired at a resolution of 60,000 at 200 m/z. The top 12 most abundant precursor ions with a charge of ≥ 2 were selected for HCD fragmentation.

Mass spectrometry data analysis. MS raw files were processed with MaxQuant software, version 1.5.0.0 (14). The raw files were searched against the mouse, *Mus musculus,* UniProt-

SwissProt FASTA database (16,997 proteins) for MS/MS based peptide identification via Andromeda. Enzyme specificity was set to trypsin, with a minimum number of seven amino acids required for peptide identification, a maximum of two missed cleavages and the FDR was set to <1% at the peptide and protein level. Default settings were used for variable and fixed modifications (variable modification; acetylation (N-terminus) and methionine oxidation; fixed modifications; carbamidomethylation). A target-decoy approach to identify peptides and proteins at an FDR <1%(15). Peptide identification was performed with the precursor mass deviation up to 4.5 ppm after time-dependent mass calibration and an allowed fragment mass deviation of 20 ppm. For label-free protein quantification, the MaxLFQ algorithm was employed for intensity determination and normalisation procedures and is fully compatible with peptide and protein separation prior to MS analysis. The minimum ratio count was set to two (16). Quantitative of high-resolution peptide profiles was based on mass-to-charge (*m/z*), retention time and intensity values. MaxQuant was used to calculate pairwise protein ratios by taking the median of all pairwise peptide ratios per protein. Only shared identical peptides were considered for each pairwise comparison, with a minimum number of one ratio count required for each pairwise comparison and a least-squares analysis was employed to rebuild the relative abundance profile for individual proteins. This facilitated the conservation of the total summed intensity for a protein across all the samples under analysis. 'Match between runs' was enabled for sample processing to maximise the number of quantification events across samples, permitting the quantification of high-resolution MS1 features that were not identified by MS2 in each single measurement (17). For matching between runs the retention time alignment window was set to 30 min and the match time window was 1 min.

Northern blot. 8-12 µg of RNA was separated on 1.2% agarose MOPS-EDTA-Sodium Acetate (Sigma-Aldrich) gels containing 0.66 M formaldehyde (Sigma-aldrich) and transferred onto Hybond-N+ membrane (GE Healthcare) by capillary transfer with 10xSSC. Blots were hybridized with random-primed 32P-labeled (AflII-HindIII fragment of *DMPK* cDNA) probe in a hybridization buffer (2% SDS, 10% dextran sulfate, 1xSSPE, 100 µg/ml salmon sperm DNA, 2% Denhart's solution) at 65°C overnight. Signals were analyzed on a phosphoimager (Molecular Imager FX, Bio-Rad) and quantified using Quantity One (Bio-Rad). All values were normalized to *18S* rRNA signal after hybridization with 5'-end 32P-labeled *18S* rRNA-oligonucleotide probes.

ELISA based measurements of oligonucleotide concentrations in tissues. Hybridization-Based ELISAs to determine the concentration of PMO oligonucleotides were performed as described (18) using phosphorothioate probes (Sequence (5'->3') [DIG]C*T*G*C*T*G*C*TGCTGCT*G*C*T*G*C*T*G[BIO]) double-labelled with digoxigenin and biotin. This probe was used to detect Pip6a-PMO or naked PMO concentrations in eight different tissues (brain, kidney, liver, lung, heart, diaphragm, gastrocnemius and quadriceps) from treated HSA-LR mice.

Statistical analysis. All group data are expressed as mean +/- SEM. Between groups, the comparison was performed by Mann-Whitney test or one-way ANOVA test followed by a Newman-Keuls or Tukey post-test using Prism 6 software (GraphPad Software, Inc.).

Differences between groups were considered significant when *P*<0.05 (*, *P*<0.05; **, *P*<0.01; ***, *P*<0.001, ****, P<0,0001). For proteomics data handling, normalization, statistics and annotation enrichment analysis, open-source bioinformatics platforms Perseus (version 1.5.5.3)(19) or R-studio (version 0.99.903) (R Development Core team, 2011) were employed. For pairwise comparison of proteomes a two-sides *t*-test statistic was employed.

Data availability: Complete raw data generated from RNA sequencing were deposited in the Gene Expression omnibus database (GEO ; GSE134926). Codes used for analyzing are available from the corresponding author on reasonable request.

Supplemental references

1. Betts C et al. Pip6-PMO, A New Generation of Peptide-oligonucleotide Conjugates With Improved Cardiac Exon Skipping Activity for DMD Treatment. *Molecular Therapy: Nucleic Acid* 2012;1:e38.

2. Delacroix C et al. Improvement of Dystrophic Muscle Fragility by Short-Term Voluntary Exercise through Activation of Calcineurin Pathway in mdx Mice. *AJPA* 2018;188(11):2662–2673.

3. Arandel L et al. Immortalized human myotonic dystrophy muscle cell lines to assess therapeutic compounds. *Dis Model Mech* 2017;10(4):487–497.

4. Huguet A et al. Molecular, Physiological, and Motor Performance Defects in DMSXL Mice Carrying >1,000 CTG Repeats from the Human DM1 Locus. *PLoS Genet* 2012;8(11):e1003043–19.

5. Chaouch S et al. Immortalized Skin Fibroblasts Expressing Conditional MyoD as a Renewable and Reliable Source of Converted Human Muscle Cells to Assess Therapeutic Strategies for Muscular Dystrophies: Validation of an Exon-Skipping Approach to Restore Dystrophin in Duchenne Muscular Dystrophy Cells. *Human Gene Therapy* 2009;20(7):784–790.

6. Francois V et al. Selective silencing of mutated mRNAs in DM1 by using modified hU7-snRNAs. *Nat. Struct. Mol. Biol.* 2011;18(1):85–87.

7. Klein AF et al. PABPN1 polyalanine tract deletion and long expansions modify its aggregation pattern and expression. *Exp. Cell Res.* 2008;314(8):1652–1666.

8. Tsanov N et al. smiFISH and FISH-quant – a flexible single RNA detection approach with super-resolution capability. *Nucleic Acids Res.* 2016;44(22):e165–e165.

9. Kim D, Langmead B, Salzberg SL. HISAT: a fast spliced aligner with low memory requirements. *Nat Methods* 2015;12(4):357–360.

10. Anders S, Reyes A, Huber W. Detecting differential usage of exons from RNA-seq data. *Genome Res.* 2012;22(10):2008–2017.

11. Love MI, Huber W, Anders S. Moderated estimation of fold change and dispersion for RNA-seq data with DESeq2. *Genome Biol.* 2014;15(12):550.

12. Liao Y, Smyth GK, Shi W. featureCounts: an efficient general purpose program for assigning sequence reads to genomic features. *Bioinformatics* 2014;30(7):923–930.

13. Holland A et al. Label-free mass spectrometric analysis of the mdx-4cv diaphragm identifies the matricellular protein periostin as a potential factor involved in dystrophinopathy-related fibrosis. *Proteomics* 2015;15(13):2318–2331.

14. Cox J, Mann M. MaxQuant enables high peptide identification rates, individualized p.p.b.-range mass accuracies and proteome-wide protein quantification. *Nat Biotechnol* 2008;26(12):1367–1372.

15. Cox J et al. Andromeda: a peptide search engine integrated into the MaxQuant environment. *J. Proteome Res.* 2011;10(4):1794–1805.

16. Cox J et al. Accurate proteome-wide label-free quantification by delayed normalization and maximal peptide ratio extraction, termed MaxLFQ. *Mol. Cell Proteomics* 2014;13(9):2513–2526.

17. Coscia F et al. Integrative proteomic profiling of ovarian cancer cell lines reveals precursor cell associated proteins and functional status. *Nat Commun* 2016;7:12645.

18. Burki U et al. Development and Application of an Ultrasensitive Hybridization-Based ELISA Method for the Determination of Peptide-Conjugated Phosphorodiamidate Morpholino Oligonucleotides. *Nucleic Acid Therapeutics* 2015;25(5):275–284.

19. Tyanova S et al. The Perseus computational platform for comprehensive analysis of (prote)omics data. *Nat Methods* 2016;13(9):731–740.

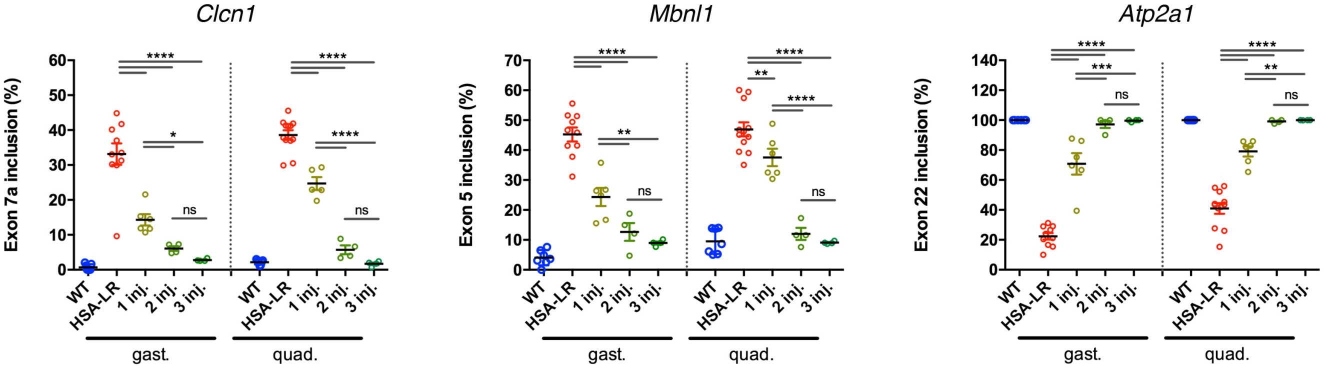

Supplementary figure 1: Dose-dependent effect of Pip6a-PMO on alternative splicing correction in HSA-LR mice. Quantification of the alternative splicing correction in HSA-LR mice treated with multiple intravenous systemic injections of Pip6a-PMO-CAG7 (pip6a-PMO) at the dose of 12.5 mg/kg and sacrificed after 2 weeks (n=7 fot WT, n=10 for HSA-LR, n=6 for 1 inj., n=4 for 2 inj. and 3 inj.). *Data are expressed as mean +/- SEM. Statistics: One-way ANOVA with Newman-Keuls post-test; *, P<0.05; **, P<0.01; ***, P<0.001; ****, P<0.0001.*

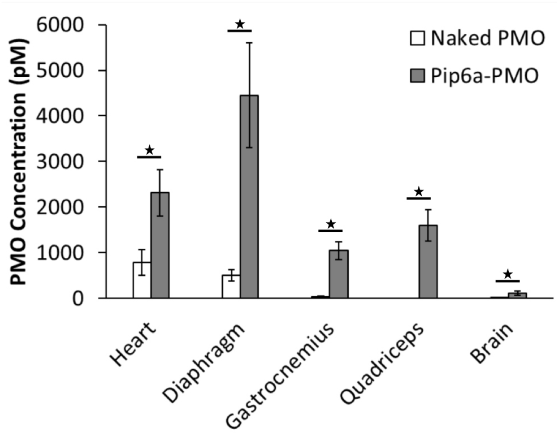

	Naked PMO (pM)	Pip6a-PMO (pM)
Kidney	321974.7	508740.8
Liver	2300.2	175984.6
Heart	781.9	2314.7
Diaphragm	504.7	4448.0
Gastrocnemius	33.9	1047.7
Quadriceps	N.D.	1602.2
Brain	3.4	118.0

Supplementary figure 2: Evaluation of Pip6a-PMO biodistribution reveals optimal delivery to critically affected tissues in DM1. PMOs were detected by a custom ELISA assay using probes labelled with digoxigenin and biotin after 3 intravenous systemic injections of Pip6a-PMO-CAG7 (Pip6a-PMO) at 12.5 mg/kg or 3x200 mg/kg of naked PMO-CAG7 (Naked PMO) in HSA-LR mice. Two weeks after the Pip6a-PMO injections the concentration of PMO in muscle tissues was still >1nM vs the low pM detected after naked PMO injections (despite of the >20-fold difference in molarity of naked PMO vs Pip6a-PMO treatments) (n=4)*. Data are expressed as mean +/- SEM. Statistics: One-way ANOVA with Tukey post-test; *, P<0.05.*

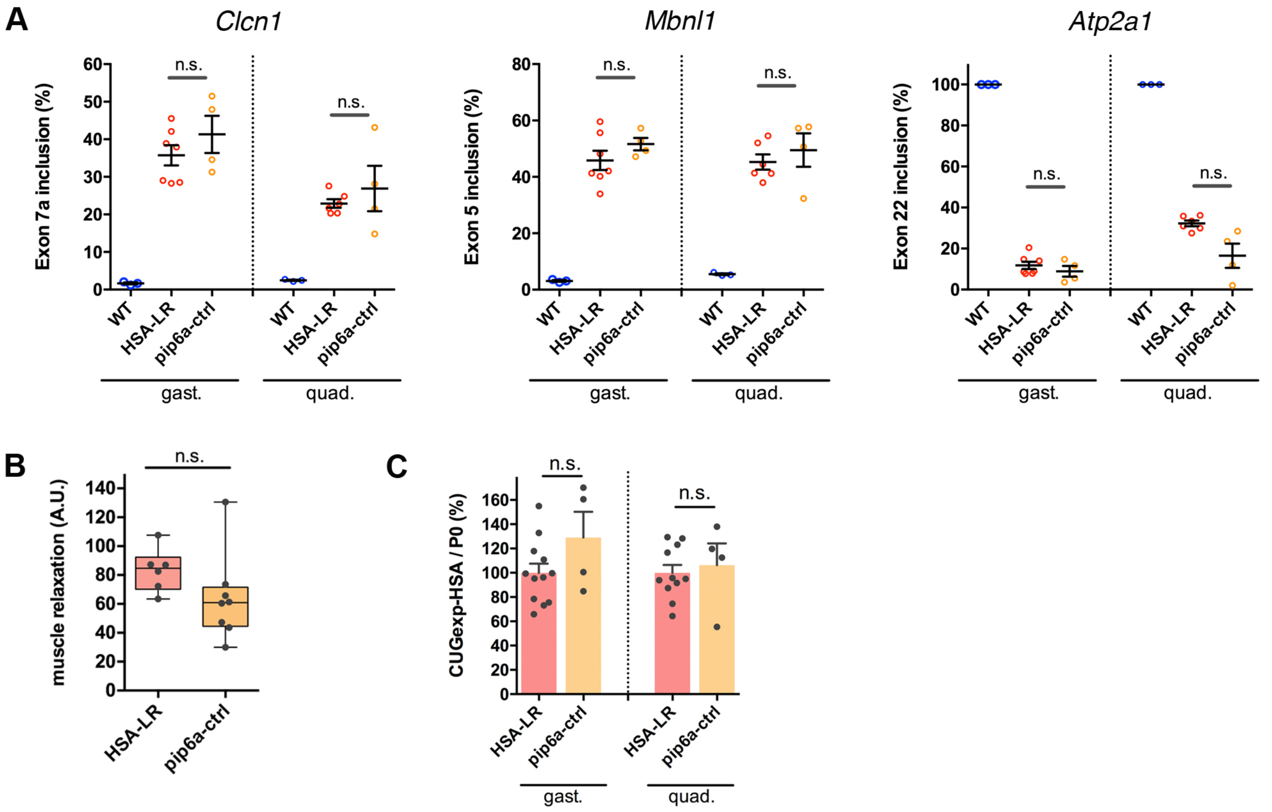

Supplementary figure 3: Treatment with Pip6a-Ctrl (GAC7) has no effect in HSA-LR mice. HSA-LR mice are treated with 3 systemic IV injections of Pip6a-PMO-GAC7 (Pip6a-ctrl) at 12.5 mg/kg and sacrificed 2 weeks after. Treatment with Pip6a-ctrl has no effect on **A)** the alternative splicing profiles of *Clcn1*, *Mbnl1* and *Serca1* as analyzed by RT-PCR (n=7 for HSA-LR and n=4 for Pip6a-PMO-ctrl), **B)** in the levels of myotonia of the *gastrocnemius* as measured as the area under the force/time curve during relaxation after maximal muscle contraction (n=6 for HSA-LR and n=8 for Pip6a-PMO-ctrl) and **C)** in levels of *CUGexp HSA* transcripts as determined by real-time PCR (n=12 for HSA-LR and n=4 for Pip6a-PMO-ctrl). *Data are expressed as mean +/- SEM. Statistics: A, C: One-way ANOVA with Newman-Keuls post-test; B: Mann-Whitney test; *, P<0.05; **, P<0.01; ***, P<0.001.*

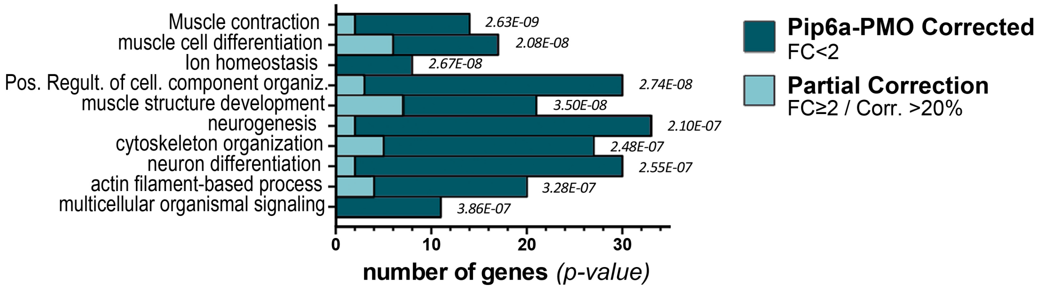

Supplementary figure 4: Treatment with Pip6a-PMO normalizes deregulated pathways in HSA-LR gastrocnemius. Number of genes, p-values and correction level for significantly enriched Biological Process_GO_terms and Pathways in the set of HSA-LR/WT missplicing events using DAVID and Toppgene tools.

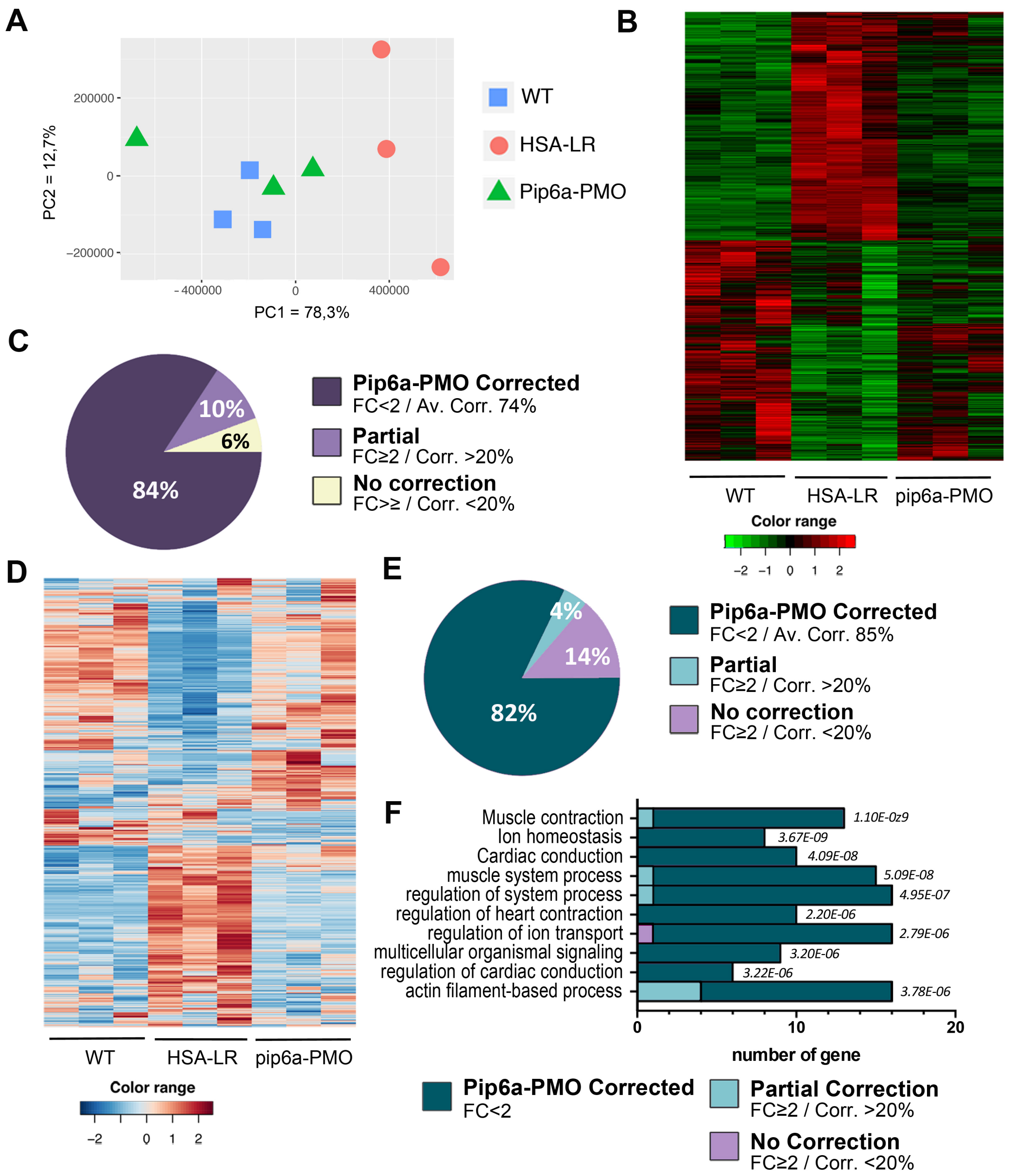

Supplementary figure 5: Treatment with Pip6a-PMO normalizes global transcriptome at both expression and splicing levels. Transcriptomic analysis by RNA sequencing was performed on total RNA of the *quadriceps* muscle of treated mice compared to HSA-LR and wild-type mice (n=3). **A-B)** Principal component analysis (A) and heatmap graphic (bB) of all the significantly expressed transcripts (adj. p-value<0.1) of *quadriceps* muscle from either WT, HSA-LR or Pip6a-PMO-CAG7 (Pip6a-PMO) treated mice reveal a global correction of genes expression profile with Pip6a-PMO treatment. **C)** Treatment with Pip6a-PMO induces the correction of the expression profile in *quadriceps* muscle of treated mice (n=588; FC≥2, adj. p-value<0.1): 84% of transcripts return to FC<2 with an average correction index of 76% ; 10% of transcripts remains at FC>2 but with correction index >20%; 6% of transcripts are not corrected. **D)** Heatmap graphic of all significant deregulated exon_bin (normalized counts) reveals a global correction of missplicing events with Pip6a-PMO treatment. **E)** Treatment with Pip6a-PMO induces a correction of alternative splicing profiles in *quadriceps* muscle of treated mice (n=242 splicing events; FC≥2, adj. p-value<0.1): 82% of events return to FC<2 with an average correction index of 83%; 4% remains at FC>2 but with correction index >20%; 14% are not corrected. **F)** Number of genes and p-values for significantly enriched Biological Process_GO_terms and Pathways in the set of HSA-LR/WT missplicing events using DAVID and Toppgene tools.

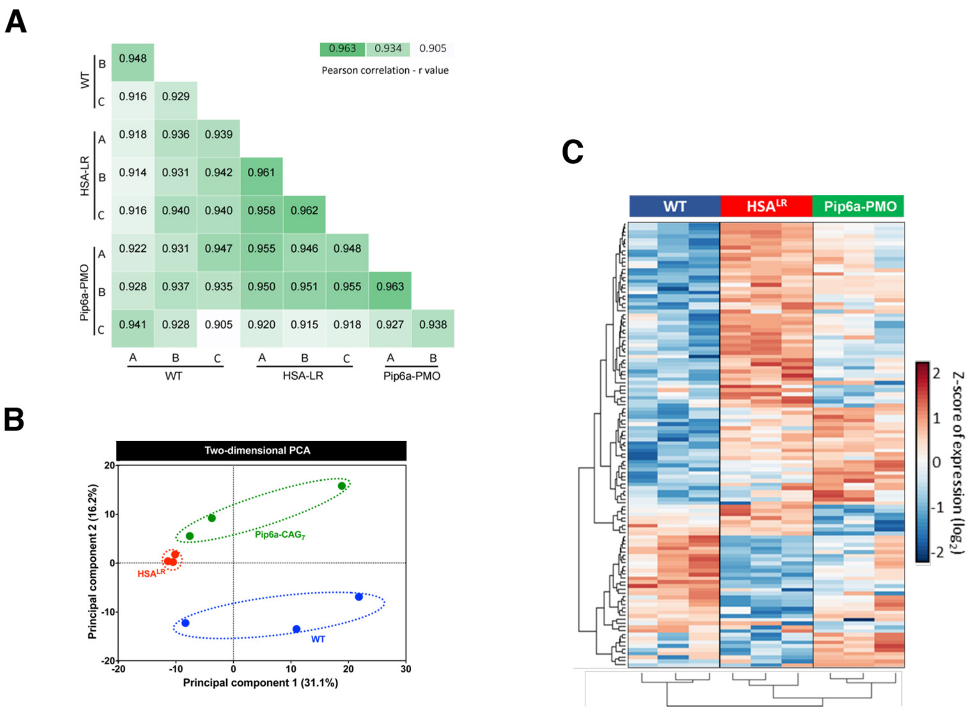

D

	Protein name	Gene name	Accession number	Fold change	Unique peptides	Unique sequence coverage [%]
Increased	Ubiquitin carboxyl-terminal hydrolase isozyme L1	Uchl1	Q9R0P9	29.02	8	56.5
Increased	Musculoskeletal embryonic nuclear protein 1	Mustn1	Q99JI1	20.69	3	48.8
Increased	DnaJ homolog subfamily B member 4	Dnajb4	Q9D832	9.52	7	37.7
Increased	Histone H1.0N-terminally processed	H1f0	P10922	8.12	3	16.5
Increased	Heat shock protein beta-7	Hspb7	P35385	6.85	8	68.0
Increased	Heat shock protein beta-1	Hspb1	P14602	6.25	20	90.9
Decreased	Leukotriene A-4 hydrolase	Lta4h	P24527	-4.28	5	11.5
Decreased	Alpha-2-macroglobulin receptor-associated protein	Lrpap1	P55302	-4.64	3	8.3
Decreased	Ubiquitin thioesterase OTUB1	Otub1	Q7TQI3	-5.26	3	12.2
Decreased	PDZ and LIM domain protein 7	Pdlim7	Q3TJD7	-5.28	9	23.0
Decreased	Dermatopontin	Dpt	Q9QZZ6	-5.64	2	16.4
Decreased	Ig gamma-1 chain C region, membrane-bound form	Ighg1	P01869	-8.16	2	13.6

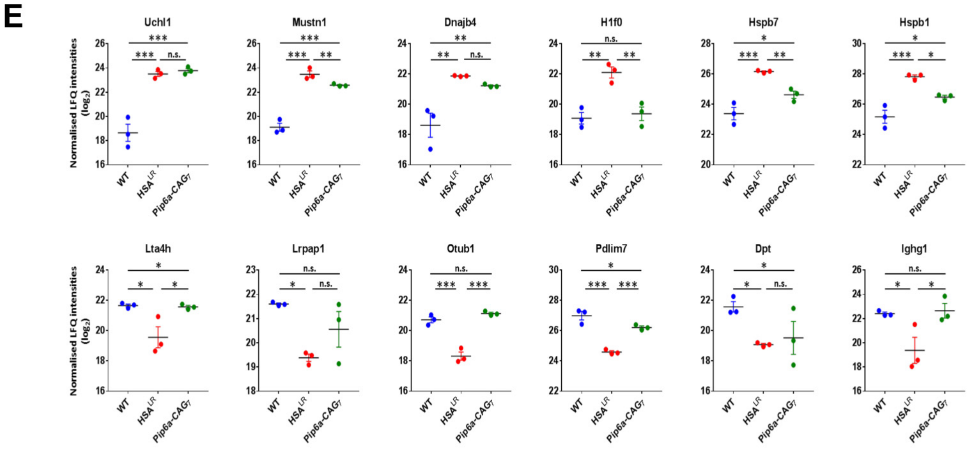

Supplementary figure 6: Treatment with Pip6a-PMO normalizes the DM1 proteomic profile of HSA-LR mice.

Supplementary figure 6: Treatment with Pip6a-PMO normalizes the DM1 proteomic profile of HSA-LR mice. *Quadriceps* muscle samples from WT, HSA-LR and Pip6a-PMO-CAG7 (Pip6a-PMO) treated HSA-LR mice were analyzed by label-free mass spectrometry. **A)** Correlation plots for quadriceps muscle from WT, HSA-LR and Pip6a-PMO treated HSA-LR mice (n=3 biological replicates per group). Each plot compares the quantitative intensity measurement of LC-MS features between two replicates. The Pearson correlation r-values demonstrate high reproducibility between and across all data sets, with a minimum r-value of 0.905 and a maximum of 0.963. **B)** Principal component analysis (PCA) illustrates a clear separation of the WT (blue), HSA-LR (red) and Pip6a-PMO treated HSA-LR (green) proteome, based on component 1 and component 2, which account for 31.1% and 16.2% of variability, respectively. PCA analysis illustrates Pip6a-PMO treated HSA-LR mice have a protein profile shift from a HSA-LR profile towards a WT profile, indicating Pip6a-PMO treatment can induce protein normalisation. **C)** Heatmap of 118 significant differentially expressed proteins from WT, HSA-LR and Pip6a-PMO treated HSA-LR mice. Differentially expressed proteins were analysed by hierarchical clustering of the z-scored normalised LFQ intensities (log2) across all experimental groups. Red indicates increased protein abundance and blue indicates decreased protein abundance. **D)** Table with the top 6 increased and decreased proteins from quadriceps muscle from HSA-LR versus WT mice as revealed by label-free mass spectrometry analysis. **E)** Dot plots of specific proteins of interest identified from outlining the dynamic change of normalised LFQ intensities (log2) from quadriceps muscle samples from WT, HSA-LR and Pip6a-PMO treated HSA-LR mice. *Data are expressed as mean +/- SEM. Statistics: One-way ANOVA and Bonferroni post hoc test; n.s., not significant; *, $P \leq 0.05$; **, $P \leq 0.01$; ***, $P \leq 0.001$.*

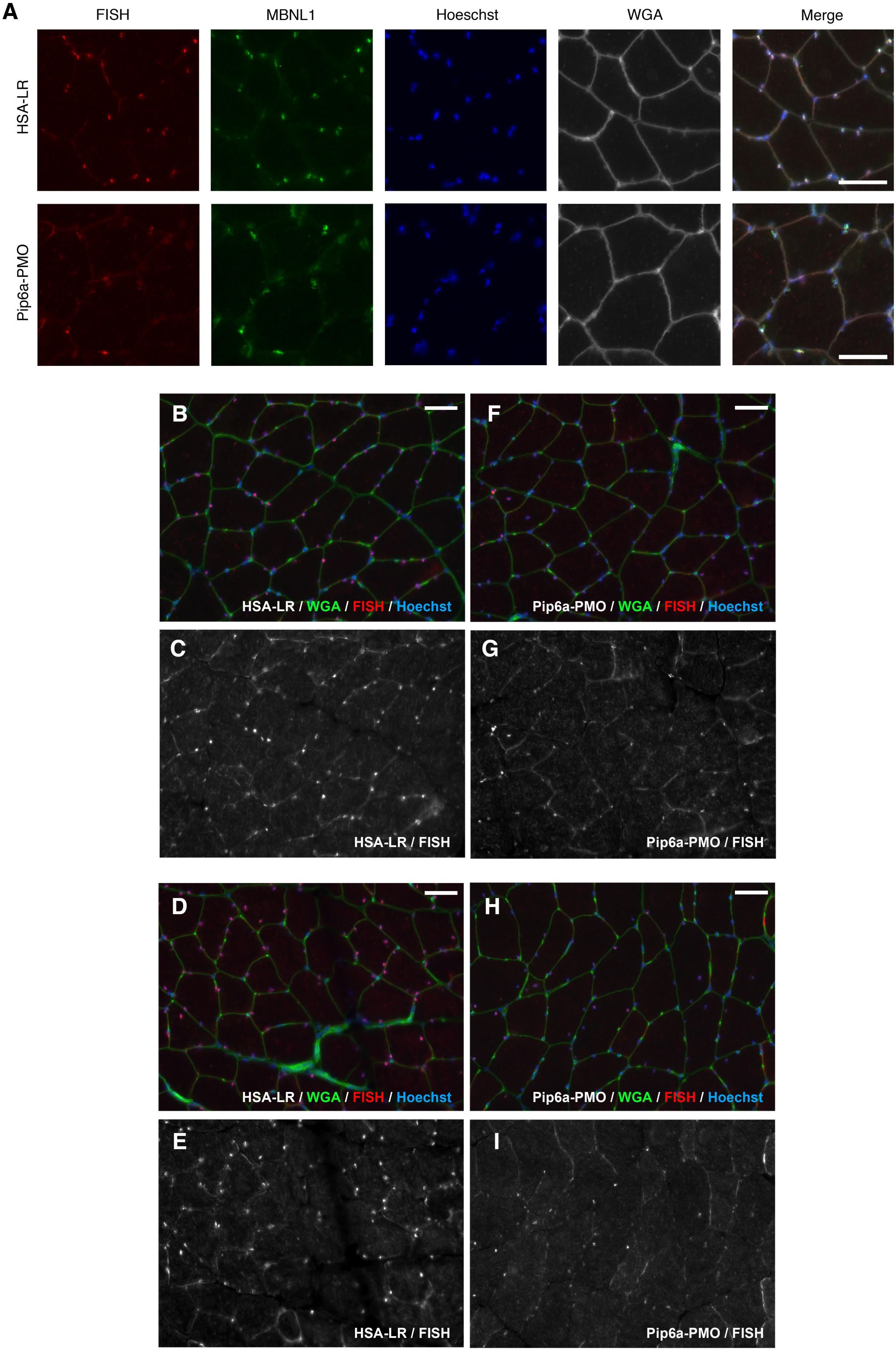
A
FISH
MBNL1
Hoeschst
WGA
Merge
HSA-LR
Pip6a-PMO
B
HSA-LR / WGA / FISH / Hoechst
F
Pip6a-PMO / WGA / FISH / Hoechst
C
HSA-LR / FISH
G
Pip6a-PMO / FISH
D
HSA-LR / WGA / FISH / Hoechst
H
Pip6a-PMO / WGA / FISH / Hoechst
E
HSA-LR / FISH
I
Pip6a-PMO / FISH

Supplementary figure 7: Treatment with Pip6a-PMO reduces the *foci* level in the muscles of HSA-LR mice. HSA-LR mice were treated with 3 systemic injections of Pip6a-PMO-CAG7 (Pip6a-PMO) at a 12.5 mg/kg dose and sacrificed 2 weeks after treatment. **A)** Representative pictures of gastrocnemius muscle section stained for CUGexp foci (FISH, red), Mbnl1 (green), fibers membrane (WGA, grey) and nucleus (Hoechst, blue) (scale bar : 50μm). **B and D**) Representative pictures of non-treated HSA-LR *gastrocnemius* sections stained for *CUGexp-RNA foci* (CAG probe, red), fibers membrane (WGA, green) and nucleus (Hoechst, blue). **C and E**) Same fields as in B and D where only the FISH signal is shown. **F and H**) Representative pictures of Pip6a-PMO treated HSA-LR *gastrocnemius* stained for *CUGexp-RNA foci* (CAG probe, red), fibers membrane (WGA, green) and Hoechst (blue). **G and I**) Same fields as in F and H where only the FISH signal is shown. Scale bar: 50 μm.

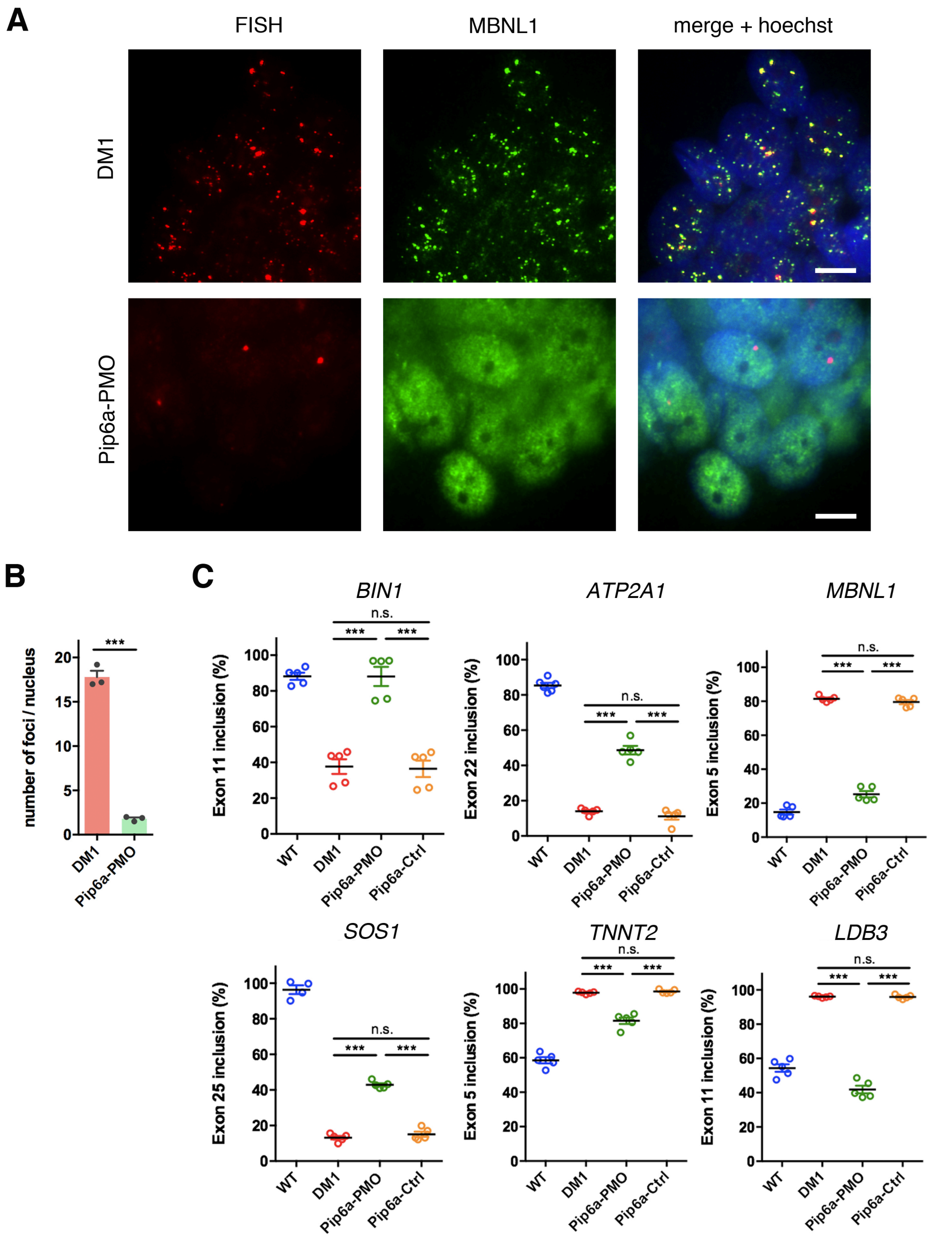

Supplementary Figure 8: Pip6a-PMO corrects DM1-specific molecular symptoms in trans-differentiated muscle cells. Four days trans-differentiated immortalized DM1 fibroblasts (1300 CTG repeats) are treated with Pip6a-PMO-CAG7 (Pip6a-PMO) at 1 µM and analyzed after 24h. **A)** FISH (CAG probe, red) /immuno-fluorescence (MBNL1, green) on DM1 or WT differentiated cells treated with Pip6a-PMO (Scale bar: 10 µm). **B)** Quantification of mean number of foci per nucleus in treated DM1 differentiated cells (n=3; >300 nucleus per n). **C)** Quantification of splicing corrections, using RT-PCR, induced by Pip6a-PMO treatment in immortalized DM1 trans-differentiated muscle cells (n=5). *Data are expressed as mean +/- SEM. Statistics: B, t-test ; C, One-way ANOVA with Newman-Keuls post-test; *, P<0.05; **, P<0.01; ***, P<0.001 ; ns: non-significant.*

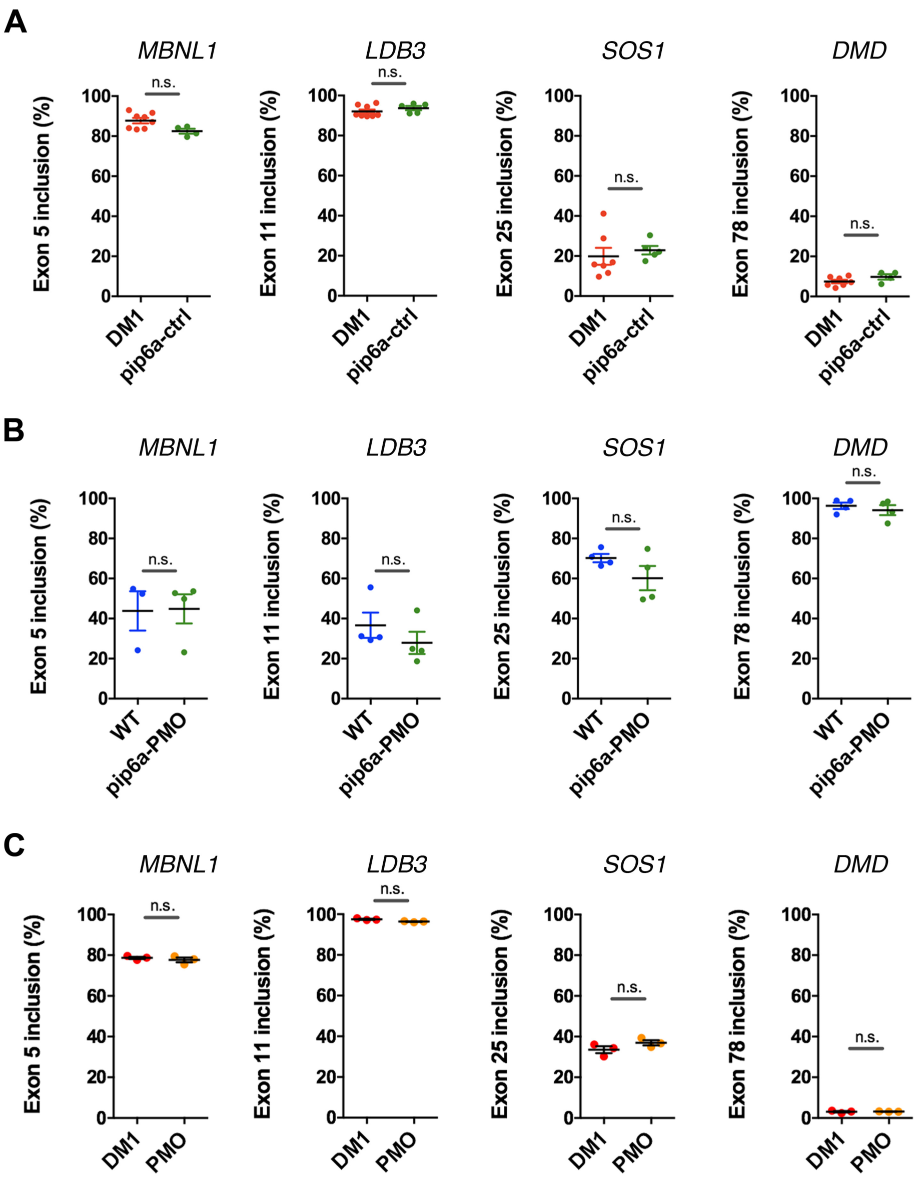

Supplementary figure 9: Effect of Pip6a-PMO on alternative splicing profiles is specific to DM1 cells and CAG antisense sequence. A) Quantification of splicing changes of *LDB3*, *MBNL1*, *SOS1*, *DMD* transcripts in 4 days differentiated immortalized DM1 (2600 CTG repeats) myoblasts treated with Pip6a-PMO-Control (Pip6a-ctrl) at 1 µM and analyzed after 24 h (n=4). **B)** Quantification of splicing changes of *LDB3*, *MBNL1*, *SOS1*, *DMD* transcripts in 4 days differentiated immortalized WT myoblasts treated with Pip6a-PMO-CAG7 (Pip6a-PMO) at 1 µM and analyzed after 24 h (n=4). C) Quantification of splicing changes of *LDB3*, *MBNL1*, *SOS1*, *DMD* transcripts in 4 days differentiated immortalized DM1 (2600 CTG repeats) myoblasts treated with unconjugated PMO-CAG (PMO) at 1 µM and analyzed after 24 h (n=3). *Data are expressed as mean +/- SEM. Statistics: One-way ANOVA with Newman-Keuls post-test; ns: non-significant.*

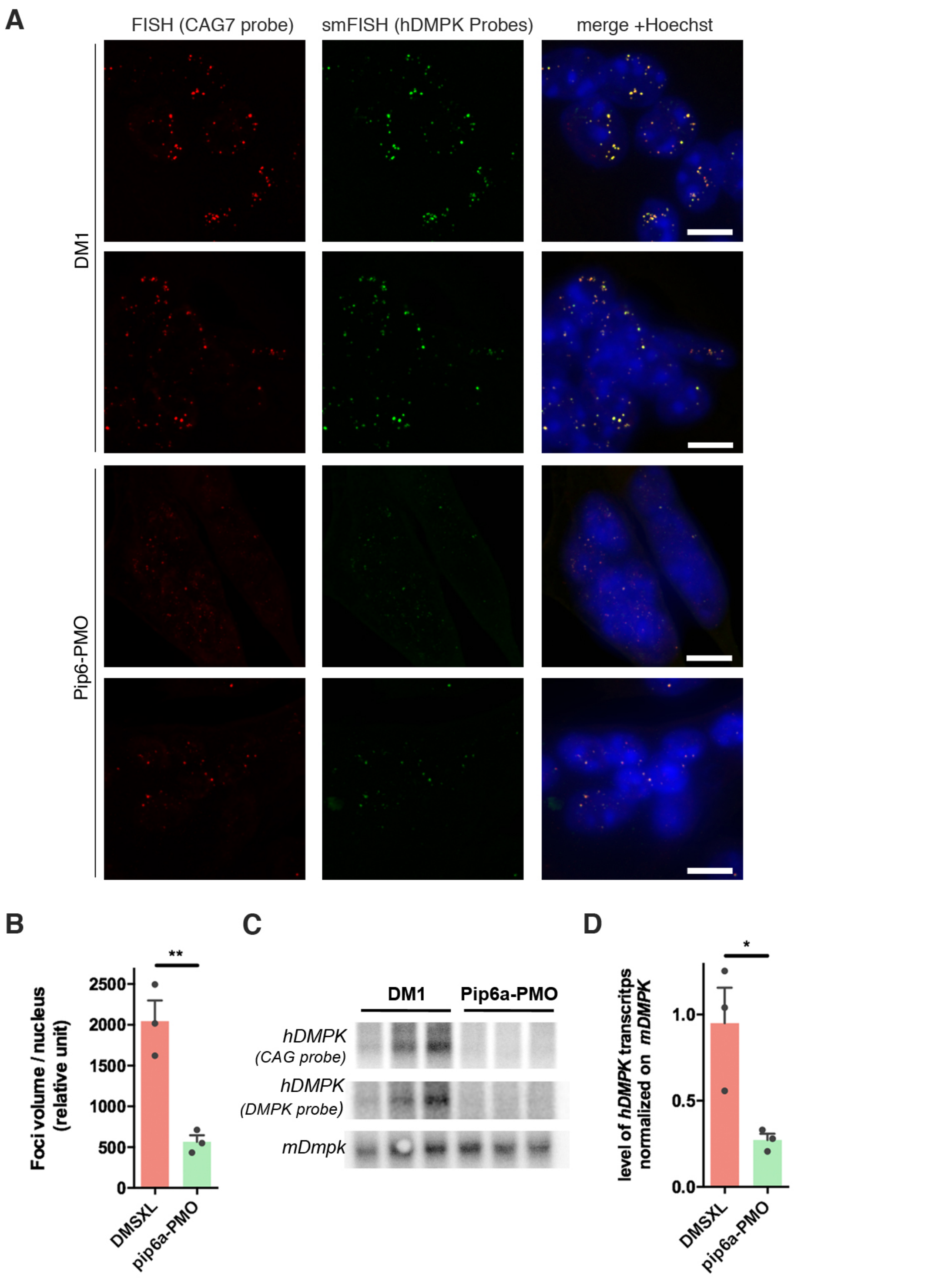

Supplementary figure 10: Pip6a-PMO treatment reduces the quantity of mutated *hDMPK* transcripts in murine DMSXL muscle cells. Three days trans-differentiated immortalized murine DMSXL cells are treated with Pip6a-PMO-CAG7 (Pip6a-PMO) at 1 µM and analyzed after 24h. **A)** FISH (CAG probe, red) /smFISH (24x *hDMPK* probes hybridized to fluorochrome-labelled FLAP oligonucleotide, green) shows that both CAG and DMPK probes exhibit a complete colocalization in DMSXL cells (both non-treated and treated) and that the number and intensity of foci is reduced in treated cells. **B)** Quantification of the foci volume (mean intensity x area of each foci using smFISH signal) in Pip6a treated cells shows an approx. 70% diminution in the quantity of mutated *hDMPK* transcript (n=3, >300 nucleus per n). **C)** Visualization by Northern blot of *hDMPK* and *mDmpk* transcripts using a CAG or a *DMPK* cDNA probe in DMSXL muscle cells treated with pip6a-PMO. **D)** Quantification by northern blot of *hDMPK* levels (normalized to *mDMPK*) shows an approx. 70% reduction in Pip6a treated cells (n=3). *Data are expressed as mean +/- SEM. Statistics: t-test; *, P<0.05; **, P<0.01.*

Supplementary table 1: list and annotation of the most deregulated genes in the gastocnemius muscles of HSA-LR and pip6a-PMO treated mice compared to WT mice.

**Table columns description:**	
Ensembl Gene ID	*ENSEMBL gene identifier*
Name	*HGNC gene name*
Description	*Gene description ans source*
Gene type	*Gene type*
Fold change (log2) WT vs. HSA-LR	*Fold change (log2) between WT and HSA-LR mice*
Fold Change (Log2) WT vs. Pip6a-PMO	*Fold change (log2) between WT and pip6a-PMO treated mice*
WT counts (mean)	*mean of the WT samples counts*
HSA-LR counts (mean)	*mean of the HSA-LR samples counts*
pip6a-PMO counts (mean)	*mean of the pip6a-PMO samples counts*
Adj. p-value WT vs. HSA-LR	*Adjusted p-values for WT vs. HSA-LR*
Adj. p-value WT vs. Pip6a-PMO	*Adjusted p-value for WT vs. Pip6a-PMO*
Correction	*percentage of correction in pip6a-PMO treated compared to HSA-LR*

Ensembl_gene_ID	Name	Description	Gene type	Fold change (Log2) WT vs. HSA	Fold change (log2) WT vs. Pip6a	WT counts (mean)	HSA-LR counts (mean)	pip6a-PMO counts (mean)	Adj. p-value WT vs. HSA-LR	Adj. p-value WT vs. Pip6a-PMO	Correction (%)
ENSMUSG00000025401	Myo1a	myosin IA [Source:MGI Symbol;Acc:MGI:107732]	protein_coding	4.149	3.198	48	2378	966	2.19e-71	1.59e-41	59.62
ENSMUSG00000037474	Dtl	denticleless E3 ubiquitin protein ligase [Source:MGI Symbol;Acc:MGI:1924093]	protein_coding	3.275	0.662	14	232	24	5.88e-48	1.19e-01	94.26
ENSMUSG00000051159	Cited1	Cbp/p300-interacting transactivator with Glu/Asp-rich carboxy-terminal domain 1 [Source:MGI Symbol;Acc:MGI:108023]	protein_coding	3.146	0.306	12	212	15	2.03e-38	7.48e-01	97.78
ENSMUSG00000027827	Kcnab1	potassium voltage-gated channel, shaker-related subfamily, beta member 1 [Source:MGI Symbol;Acc:MGI:109155]	protein_coding	-3.144	-1.597	5611	251	1159	2.43e-37	6.77e-09	18.53
ENSMUSG00000097451	Rian	RNA imprinted and accumulated in nucleus [Source:MGI Symbol;Acc:MGI:1922995]	processed_transcript	3.083	-0.681	866	8496	444	3.94e-77	2.79e-03	104.35
ENSMUSG00000058925	1700011I03Rik	RIKEN cDNA 1700011I03 gene [Source:MGI Symbol;Acc:MGI:1922694]	protein_coding	2.976	1.036	50	1059	153	1.99e-30	2.60e-03	89.39
ENSMUSG00000034206	Polq	polymerase (DNA directed), theta [Source:MGI Symbol;Acc:MGI:2155399]	protein_coding	2.65	1.453	66	505	191	7.46e-47	1.22e-12	71.3
ENSMUSG00000091078	Gm17218	predicted gene 17218 [Source:MGI Symbol;Acc:MGI:4938045]	antisense	2.61	0.84	17	238	40	8.87e-24	3.43e-02	88
ENSMUSG00000039145	Camk1d	calcium/calmodulin-dependent protein kinase ID [Source:MGI Symbol;Acc:MGI:2442190]	protein_coding	2.548	0.42	321	2089	390	1.20e-58	1.61e-01	94.63
ENSMUSG00000034457	Eda2r	ectodysplasin A2 receptor [Source:MGI Symbol;Acc:MGI:2442860]	protein_coding	2.531	0.862	1268	8266	2163	4.46e-53	1.88e-05	85.96
ENSMUSG00000032246	Calml4	calmodulin-like 4 [Source:MGI Symbol;Acc:MGI:1922850]	protein_coding	2.502	0.958	66	477	130	9.73e-37	1.50e-04	83.08
ENSMUSG00000097391	Mirg	miRNA containing gene [Source:MGI Symbol;Acc:MGI:3781106]	lincRNA	2.502	-0.215	39	309	26	4.38e-28	8.45e-01	102.97
ENSMUSG00000020798	Spns3	spinster homolog 3 [Source:MGI Symbol;Acc:MGI:1924827]	protein_coding	2.472	0.305	48	576	61	1.16e-20	7.59e-01	96.72
ENSMUSG00000029223	Uchl1	ubiquitin carboxy-terminal hydrolase L1 [Source:MGI Symbol;Acc:MGI:103149]	protein_coding	2.468	0.978	299	1758	538	1.19e-63	6.47e-09	81.65
ENSMUSG00000100410	2310020H05Rik	RIKEN cDNA 2310020H05 gene [Source:MGI Symbol;Acc:MGI:1916853]	lincRNA	2.454	1.083	179	1189	373	2.10e-36	3.09e-06	79.1
ENSMUSG00000019027	Dnah1	dynein, axonemal, heavy chain 1 [Source:MGI Symbol;Acc:MGI:107721]	protein_coding	2.434	0.751	21	167	39	4.59e-27	3.64e-02	87.11
ENSMUSG00000060275	Nrg2	neuregulin 2 [Source:MGI Symbol;Acc:MGI:1098246]	protein_coding	2.412	0.989	19	246	59	1.23e-19	5.94e-03	82.66
ENSMUSG00000060429	Sntb1	syntrophin, basic 1 [Source:MGI Symbol;Acc:MGI:101781]	protein_coding	2.405	0.51	244	1805	345	4.07e-26	2.74e-01	92.77
ENSMUSG00000042045	Sln	sarcolipin [Source:MGI Symbol;Acc:MGI:1913652]	protein_coding	2.371	0.386	339	7670	618	7.65e-17	6.66e-01	95.77
ENSMUSG00000084939	Gm830	predicted gene 830 [Source:MGI Symbol;Acc:MGI:2685676]	processed_transcript	2.367	2.267	29	209	179	7.52e-29	1.68e-25	15.6
ENSMUSG00000055214	Pld5	phospholipase D family, member 5 [Source:MGI Symbol;Acc:MGI:2442056]	protein_coding	2.356	1.787	22	248	140	4.70e-20	9.29e-11	48.35
ENSMUSG00000040828	Catsperd	catsper channel auxiliary subunit delta [Source:MGI Symbol;Acc:MGI:2147030]	protein_coding	2.354	0.239	5	103	8	5.29e-17	8.55e-01	97.08
ENSMUSG00000022865	Cxadr	coxsackie virus and adenovirus receptor [Source:MGI Symbol;Acc:MGI:1201679]	protein_coding	2.338	0.124	40	281	38	1.22e-24	9.32e-01	98.61
ENSMUSG00000028080	Lrba	LPS-responsive beige-like anchor [Source:MGI Symbol;Acc:MGI:1933162]	protein_coding	2.333	2.123	601	3381	2768	3.56e-44	7.14e-36	24.5
ENSMUSG00000075330	A930003A15Rik	RIKEN cDNA A930003A15 gene [Source:MGI Symbol;Acc:MGI:1915412]	protein_coding	2.301	0.7	25	181	43	1.35e-22	7.62e-02	86.97
ENSMUSG00000049928	Glp2r	glucagon-like peptide 2 receptor [Source:MGI Symbol;Acc:MGI:2136733]	protein_coding	2.285	0.477	13	120	21	8.31e-19	4.71e-01	92.28
ENSMUSG00000100550	2310039L15Rik	RIKEN cDNA 2310039L15 gene [Source:MGI Symbol;Acc:MGI:1916879]	lincRNA	2.284	0.306	7	76	9	3.84e-17	7.69e-01	95.3
ENSMUSG00000024524	Gnal	guanine nucleotide binding protein, alpha stimulating, olfactory type [Source:MGI Symbol;Acc:MGI:95774]	protein_coding	2.253	0.736	76	507	132	8.86e-23	3.60e-02	86.16
ENSMUSG00000021913	Ogdhl	oxoglutarate dehydrogenase-like [Source:MGI Symbol;Acc:MGI:3616088]	protein_coding	2.246	0.433	248	1657	331	8.86e-23	4.34e-01	93.17
ENSMUSG00000015745	Plekho1	pleckstrin homology domain containing, family O member 1 [Source:MGI Symbol;Acc:MGI:1914470]	protein_coding	2.245	1.481	908	5001	2510	7.94e-34	8.51e-14	58.27
ENSMUSG00000036882	Arhgap33	Rho GTPase activating protein 33 [Source:MGI Symbol;Acc:MGI:2673998]	protein_coding	2.22	1.162	60	510	187	9.71e-18	2.73e-04	72.78
ENSMUSG00000101227	Gm29506	predicted gene 29506 [Source:MGI Symbol;Acc:MGI:5580212]	sense_overlapping	2.201	1.377	24	164	77	5.08e-22	1.30e-07	60.64
ENSMUSG00000017716	Birc5	baculoviral IAP repeat-containing 5 [Source:MGI Symbol;Acc:MGI:1203517]	protein_coding	2.2	0.052	15	108	14	1.36e-18	9.75e-01	100.01
ENSMUSG00000021268	Meg3	maternally expressed 3 [Source:MGI Symbol;Acc:MGI:1202886]	lincRNA	2.2	-0.611	1335	6378	743	1.43e-52	1.55e-03	109.05
ENSMUSG00000042254	Cilp	cartilage intermediate layer protein, nucleotide pyrophosphohydrolase [Source:MGI Symbol;Acc:MGI:2444507]	protein_coding	2.2	0.075	3540	23270	3292	1.03e-19	9.65e-01	99.4
ENSMUSG00000028185	Dnase2b	deoxyribonuclease II beta [Source:MGI Symbol;Acc:MGI:1913283]	protein_coding	2.185	0.891	3	60	14	7.85e-15	3.21e-02	79.79
ENSMUSG00000026147	Col9a1	collagen, type IX, alpha 1 [Source:MGI Symbol;Acc:MGI:88465]	protein_coding	2.18	1.843	53	407	282	8.31e-19	1.46e-12	34.45
ENSMUSG00000075408	6030408B16Rik	RIKEN cDNA 6030408B16 gene [Source:MGI Symbol;Acc:MGI:1924967]	lincRNA	2.178	0.46	9	77	14	1.03e-16	5.16e-01	90.74
ENSMUSG00000079049	Serpinb1c	serine (or cysteine) peptidase inhibitor, clade B, member 1c [Source:MGI Symbol;Acc:MGI:2445363]	protein_coding	2.137	2.074	83	393	353	2.92e-42	7.07e-39	13.35
ENSMUSG00000068606	Gm4841	predicted gene 4841 [Source:MGI Symbol;Acc:MGI:3643814]	protein_coding	-2.11	-0.271	1679	232	1105	1.14e-19	7.47e-01	71.77
ENSMUSG00000048388	Fam171b	family with sequence similarity 171, member B [Source:MGI Symbol;Acc:MGI:2444579]	protein_coding	2.106	0.083	89	463	83	2.27e-24	9.54e-01	99.25
ENSMUSG00000101257	2310015K22Rik	RIKEN cDNA 2310015K22 gene [Source:MGI Symbol;Acc:MGI:1916815]	lincRNA	2.067	1.028	78	388	160	2.11e-26	1.18e-05	72.13
ENSMUSG00000025789	St8sia2	ST8 alpha-N-acetyl-neuraminide alpha-2,8-sialyltransferase 2 [Source:MGI Symbol;Acc:MGI:106020]	protein_coding	2.04	0.099	36	187	34	1.76e-19	9.50e-01	98.9
ENSMUSG00000026764	Kif5c	kinesin family member 5C [Source:MGI Symbol;Acc:MGI:1098269]	protein_coding	2.04	1.572	38	341	193	5.93e-14	1.59e-07	45.6
ENSMUSG00000029026	Trp73	transformation related protein 73 [Source:MGI Symbol;Acc:MGI:1336991]	protein_coding	2.031	2.427	19	152	209	2.92e-15	4.91e-21	-36.53
ENSMUSG00000006642	Tcf23	transcription factor 23 [Source:MGI Symbol;Acc:MGI:1934960]	protein_coding	2.025	0.87	31	222	74	7.33e-15	1.90e-02	78.35
ENSMUSG00000031772	Cntnap4	contactin associated protein-like 4 [Source:MGI Symbol;Acc:MGI:2183572]	protein_coding	2.017	0.507	6	76	13	1.28e-12	4.60e-01	89.24

ENSMUSG00000035539	Ccdc180	coiled-coil domain containing 180 [Source:MGI Symbol;Acc:MGI:2685871]	protein_coding	2.014	0.203	1	55	3	**1.02e-12**	**8.85e-01**	96.66
ENSMUSG00000072875	Gpr27	G protein-coupled receptor 27 [Source:MGI Symbol;Acc:MGI:1202299]	protein_coding	2.008	0.946	13	81	31	**1.76e-16**	**4.23e-03**	72.58
ENSMUSG00000073730	4933415F23Rik	RIKEN cDNA 4933415F23 gene [Source:MGI Symbol;Acc:MGI:1914005]	protein_coding	1.995	1.35	122	599	326	**1.20e-21**	**4.59e-09**	54.62
ENSMUSG00000028445	Enho	energy homeostasis associated [Source:MGI Symbol;Acc:MGI:1916888]	protein_coding	-1.99	-0.762	1377	283	672	**3.17e-38**	**3.38e-05**	40.99
ENSMUSG00000000794	Kcnn3	potassium intermediate/small conductance calcium-activated channel, subfamily N, member 3 [Source:MGI Symbol;Acc:MGI:2153183]	protein_coding	1.986	0.714	119	631	202	**3.06e-17**	**4.99e-02**	82.79
ENSMUSG00000085455	1810059H22Rik	RIKEN cDNA 1810059H22 gene		1.984	0.472	19	108	27	**3.66e-16**	**4.32e-01**	89.05
ENSMUSG00000040187	Arntl2	aryl hydrocarbon receptor nuclear translocator-like 2 [Source:MGI Symbol;Acc:MGI:2684845]	protein_coding	-1.975	-0.6	70	6	34	**7.89e-13**	**2.39e-01**	50.22
ENSMUSG00000007279	Scube2	signal peptide, CUB domain, EGF-like 2 [Source:MGI Symbol;Acc:MGI:1928765]	protein_coding	1.973	0.325	138	702	164	**1.34e-17**	**6.56e-01**	93.76
ENSMUSG00000037996	Slc24a2	solute carrier family 24 (sodium/potassium/calcium exchanger), member 2 [Source:MGI Symbol;Acc:MGI:1923626]	protein_coding	1.943	0.84	63	279	109	**1.44e-23**	**9.78e-04**	76.32
ENSMUSG00000024127	Prepl	prolyl endopeptidase-like [Source:MGI Symbol;Acc:MGI:2441932]	protein_coding	1.932	0.309	1066	4267	1190	**5.00e-34**	**4.23e-01**	93.61
ENSMUSG00000058019	Ces5a	carboxylesterase 5A [Source:MGI Symbol;Acc:MGI:1915185]	protein_coding	1.925	-0.111	28	207	20	**8.79e-12**	**9.53e-01**	102.57
ENSMUSG00000074895	Eif4e1b	eukaryotic translation initiation factor 4E family member 1B [Source:MGI Symbol;Acc:MGI:2685119]	protein_coding	1.923	1.223	2	46	20	**2.48e-11**	**4.67e-04**	58.05
ENSMUSG00000031608	Galnt7	UDP-N-acetyl-alpha-D-galactosamine: polypeptide N-acetylgalactosaminyltransferase 7 [Source:MGI Symbol;Acc:MGI:1349449]	protein_coding	1.916	1.482	314	1350	917	**9.94e-23**	**1.12e-12**	42.83
ENSMUSG00000074973	Gm11382	predicted pseudogene 11382 [Source:MGI Symbol;Acc:MGI:3650578]	unprocessed_pseudogene	1.915	1.784	16	95	81	**8.22e-15**	**6.69e-12**	18.9
ENSMUSG00000040640	Erc2	ELKS/RAB6-interacting/CAST family member 2 [Source:MGI Symbol;Acc:MGI:1098749]	protein_coding	1.913	1.004	54	358	138	**4.39e-13**	**3.18e-03**	70.5
ENSMUSG00000034361	Cpne2	copine II [Source:MGI Symbol;Acc:MGI:2387578]	protein_coding	1.909	0.825	633	2417	1006	**1.47e-44**	**1.90e-07**	75.95
ENSMUSG00000100291	2310069B03Rik	RIKEN cDNA 2310069B03 gene [Source:MGI Symbol;Acc:MGI:1916902]	lincRNA	1.907	0.301	65	270	73	**4.55e-25**	**5.99e-01**	93.62
ENSMUSG00000050069	Grem2	gremlin 2, DAN family BMP antagonist [Source:MGI Symbol;Acc:MGI:1344367]	protein_coding	-1.905	-0.389	1156	155	710	**3.43e-13**	**6.06e-01**	61.04
ENSMUSG00000040164	Kcns1	K+ voltage-gated channel, subfamily S, 1 [Source:MGI Symbol;Acc:MGI:1197019]	protein_coding	-1.898	-1.978	45	3	2	**5.08e-11**	**3.37e-11**	-2.75
ENSMUSG00000097961	Gm27000	predicted gene, 27000 [Source:MGI Symbol;Acc:MGI:5504115]	lincRNA	1.898	-0.026	4	46	3	**5.58e-11**	**9.89e-01**	101.6
ENSMUSG00000055003	Lrtm2	leucine-rich repeats and transmembrane domains 2 [Source:MGI Symbol;Acc:MGI:2141485]	protein_coding	1.891	1.61	83	480	348	**3.46e-14**	**1.76e-09**	32.72
ENSMUSG00000085873	Ttc39aos1	Ttc39a opposite strand RNA 1 [Source:MGI Symbol;Acc:MGI:3651956]	antisense	1.885	0.344	3	38	6	**6.06e-11**	**7.27e-01**	91.99
ENSMUSG00000085847	Gm14540	predicted gene 14540 [Source:MGI Symbol;Acc:MGI:3705140]	antisense	1.844	1.467	1	38	24	**1.79e-10**	**7.01e-06**	39.01
ENSMUSG00000029769	Ccdc136	coiled-coil domain containing 136 [Source:MGI Symbol;Acc:MGI:1918128]	protein_coding	1.843	0.807	555	2078	881	**9.33e-33**	**1.50e-05**	75.26
ENSMUSG00000041660	Bbox1	butyrobetaine (gamma), 2-oxoglutarate dioxygenase 1 (gamma-butyrobetaine hydroxylase) [Source:MGI Symbol;Acc:MGI:1891372]	protein_coding	1.815	1.158	5	45	22	**1.38e-10**	**9.17e-04**	56.08
ENSMUSG00000102106	2310043O21Rik	RIKEN cDNA 2310043O21 gene [Source:MGI Symbol;Acc:MGI:1916929]	lincRNA	1.811	0.256	0	35	2	**8.88e-11**	**NA**	94.4
ENSMUSG00000086607	4930511M06Rik	RIKEN cDNA 4930511M06 gene [Source:MGI Symbol;Acc:MGI:1922334]	processed_transcript	1.799	0.19	12	79	14	**8.46e-11**	**8.98e-01**	96.27
ENSMUSG00000060843	Ctnna3	catenin (cadherin associated protein), alpha 3 [Source:MGI Symbol;Acc:MGI:2661445]	protein_coding	1.779	0.584	321	1075	428	**1.60e-56**	**4.57e-05**	82.7
ENSMUSG00000030402	Ppm1n	protein phosphatase, Mg2+/Mn2+ dependent, 1N (putative) [Source:MGI Symbol;Acc:MGI:2142330]	protein_coding	-1.776	-1.167	84	4	16	**1.08e-09**	**1.06e-03**	17.59
ENSMUSG00000047363	Cstad	CSA-conditional, T cell activation-dependent protein [Source:MGI Symbol;Acc:MGI:1925867]	protein_coding	1.769	0.095	18	144	18	**9.79e-10**	**9.60e-01**	98.57
ENSMUSG00000074071	Fam169b	family with sequence similarity 169, member B [Source:MGI Symbol;Acc:MGI:3644026]	protein_coding	1.765	0.126	39	174	39	**1.85e-13**	**9.32e-01**	97.97
ENSMUSG00000026115	Vwa3b	von Willebrand factor A domain containing 3B [Source:MGI Symbol;Acc:MGI:1918103	protein_coding	1.762	0.287	127	508	136	**3.15e-16**	**6.92e-01**	93.06
ENSMUSG00000079502	Cfap77	cilia and flagella associated protein 77 [Source:MGI Symbol;Acc:MGI:2685669]	protein_coding	1.761	0.385	10	96	17	**1.47e-09**	**6.64e-01**	90.47
ENSMUSG00000085087	Gm13528	predicted gene 13528 [Source:MGI Symbol;Acc:MGI:3651630]	antisense	1.754	1.24	6	53	30	**6.08e-10**	**2.53e-04**	48.06
ENSMUSG00000036918	Ttc7	tetratricopeptide repeat domain 7 [Source:MGI Symbol;Acc:MGI:1920999]	protein_coding	1.752	0.376	973	3238	1124	**5.34e-49**	**4.98e-02**	89.99
ENSMUSG00000052942	Glis3	GLIS family zinc finger 3 [Source:MGI Symbol;Acc:MGI:2444289]	protein_coding	1.752	0.849	29	135	57	**1.04e-12**	**1.37e-02**	71.16
ENSMUSG00000035578	Iqcg	IQ motif containing G [Source:MGI Symbol;Acc:MGI:1916957]	protein_coding	1.75	0.704	260	873	381	**1.23e-41**	**1.04e-05**	77.17
ENSMUSG00000054003	Tdrd9	tudor domain containing 9 [Source:MGI Symbol;Acc:MGI:1921941]	protein_coding	1.734	-0.005	28	146	23	**2.81e-10**	**9.97e-01**	100.97
ENSMUSG00000026114	Cnga3	cyclic nucleotide gated channel alpha 3 [Source:MGI Symbol;Acc:MGI:1341818]	protein_coding	1.722	0.279	67	246	74	**1.78e-19**	**6.57e-01**	93.09
ENSMUSG00000021456	Fbp2	fructose bisphosphatase 2 [Source:MGI Symbol;Acc:MGI:95491]	protein_coding	1.717	0.508	1186	4095	1517	**8.19e-25**	**5.41e-02**	85.23
ENSMUSG00000074136	4930513N10Rik	RIKEN cDNA 4930513N10 gene [Source:MGI Symbol;Acc:MGI:2443064]	antisense	1.716	0.53	17	83	26	**6.96e-11**	**3.57e-01**	83.6
ENSMUSG00000075225	Ccdc162	coiled-coil domain containing 162 [Source:MGI Symbol;Acc:MGI:1923223]	protein_coding	1.714	1.083	50	233	123	**6.16e-12**	**3.42e-04**	58.26
ENSMUSG00000016327	Atp1b4	ATPase, (Na+)/K+ transporting, beta 4 polypeptide [Source:MGI Symbol;Acc:MGI:1915071]	protein_coding	1.71	-0.138	281	1342	204	**1.97e-10**	**9.30e-01**	103.8
ENSMUSG00000039496	Cdnf	cerebral dopamine neurotrophic factor [Source:MGI Symbol;Acc:MGI:3606576]	protein_coding	-1.71	-0.328	3763	912	2570	**1.72e-22**	**4.36e-01**	65.37
ENSMUSG00000061126	Cyp4f39	cytochrome P450, family 4, subfamily f, polypeptide 39 [Source:MGI Symbol;Acc:MGI:2445210]	protein_coding	-1.701	-1.831	85	15	12	**1.86e-10**	**4.38e-11**	-5.41
ENSMUSG00000030433	Sbk2	SH3-binding domain kinase family, member 2 [Source:MGI Symbol;Acc:MGI:2685925]	protein_coding	1.695	1.914	3685	13060	14936	**4.76e-19**	**1.75e-23**	-15.11
ENSMUSG00000059970	Hspa2	heat shock protein 2 [Source:MGI Symbol;Acc:MGI:96243]	protein_coding	1.694	1.135	515	1760	1079	**1.66e-25**	**1.13e-10**	53.03
ENSMUSG00000023868	Pde10a	phosphodiesterase 10A [Source:MGI Symbol;Acc:MGI:1345143]	protein_coding	1.682	0.216	207	806	220	**5.16e-13**	**8.23e-01**	95.06
ENSMUSG00000026185	Igfbp5	insulin-like growth factor binding protein 5 [Source:MGI Symbol;Acc:MGI:96440]	protein_coding	-1.66	-0.59	74238	19907	41300	**9.51e-30**	**2.13e-03**	45.65
ENSMUSG00000062778	Chia1	chitinase, acidic 1 [Source:MGI Symbol;Acc:MGI:1932052]	protein_coding	1.651	0.759	10	56	22	**3.27e-09**	**8.90e-02**	72.26
ENSMUSG00000028555	Ttc39a	tetratricopeptide repeat domain 39A [Source:MGI Symbol;Acc:MGI:2444350]	protein_coding	1.647	0.517	16	71	24	**2.81e-10**	**3.70e-01**	83.39
ENSMUSG00000068547	Clca4a	chloride channel accessory 4A [Source:MGI Symbol;Acc:MGI:2139744]	protein_coding	1.64	0.163	9	73	10	**2.49e-08**	**9.25e-01**	96.26
ENSMUSG00000072884	Gm10433	predicted gene 10433 [Source:MGI Symbol;Acc:MGI:3642010]	processed_transcript	1.638	0.18	7	46	8	**2.36e-08**	**9.12e-01**	95.34
ENSMUSG00000085088	4931413K12Rik	RIKEN cDNA 4931413K12 gene [Source:MGI Symbol;Acc:MGI:1918237]	processed_transcript	1.626	1.069	149	644	359	**2.63e-10**	**6.19e-04**	55.85
ENSMUSG00000022144	Gdnf	glial cell line derived neurotrophic factor [Source:MGI Symbol;Acc:MGI:107430]	protein_coding	1.619	0.343	82	410	106	**4.27e-09**	**7.03e-01**	90.58
ENSMUSG00000029055	Plch2	phospholipase C, eta 2 [Source:MGI Symbol;Acc:MGI:2443078]	protein_coding	-1.619	-1.133	57	7	14	**3.37e-08**	**1.38e-03**	16.53
ENSMUSG00000050141	Fam205c	family with sequence similarity 205, member C [Source:MGI Symbol;Acc:MGI:2679716]	protein_coding	1.619	0.672	3	36	11	**4.29e-08**	**2.06e-01**	75.65
ENSMUSG00000025038	Efhc2	EF-hand domain (C-terminal) containing 2 [Source:MGI Symbol;Acc:MGI:1921655]	protein_coding	1.618	0.494	14	70	22	**3.66e-09**	**4.46e-01**	83.63
ENSMUSG00000040875	Osbpl10	oxysterol binding protein-like 10 [Source:MGI Symbol;Acc:MGI:1921736]	protein_coding	1.612	0.923	153	497	276	**2.49e-21**	**4.11e-06**	62.18
ENSMUSG00000024268	Celf4	CUGBP, Elav-like family member 4 [Source:MGI Symbol;Acc:MGI:1932407]	protein_coding	1.6	0.028	14	62	12	**4.95e-09**	**9.87e-01**	100.49
ENSMUSG00000048776	Pthlh	parathyroid hormone-like peptide [Source:MGI Symbol;Acc:MGI:97800]	protein_coding	1.594	0.613	115	397	168	**3.22e-14**	**6.61e-02**	78.28
ENSMUSG00000046413	Irx3os	iroquois homeobox 3, opposite strand [Source:MGI Symbol;Acc:MGI:2441953]	antisense	1.591	0.601	236	1100	408	**4.62e-09**	**2.33e-01**	80.07
ENSMUSG00000051726	Kcnf1	potassium voltage-gated channel, subfamily F, member 1 [Source:MGI Symbol;Acc:MGI:2687399]	protein_coding	-1.589	-0.281	993	171	632	**1.22e-08**	**7.89e-01**	66.66

ENSMUSG00000023913	Pla2g7	phospholipase A2, group VII (platelet-activating factor acetylhydrolase, plasma) [Source:MGI Symbol;Acc:MGI:1351327]	protein_coding	-1.586	-0.994	1567	316	524	**3.02e-09**	**2.98e-03**	19.94
ENSMUSG00000023987	Pgc	progastricsin (pepsinogen C) [Source:MGI Symbol;Acc:MGI:98909]	protein_coding	1.582	0.958	30	112	63	**1.94e-11**	**1.27e-03**	58.91
ENSMUSG00000029174	Tbc1d1	TBC1 domain family, member 1 [Source:MGI Symbol;Acc:MGI:1889508]	protein_coding	1.581	0.334	3793	13523	4523	**1.38e-11**	**6.26e-01**	90.29
ENSMUSG00000029771	Irf5	interferon regulatory factor 5 [Source:MGI Symbol;Acc:MGI:1350924]	protein_coding	1.576	-0.194	429	1483	313	**2.33e-12**	**8.46e-01**	106.32
ENSMUSG00000030616	Sytl2	synaptotagmin-like 2 [Source:MGI Symbol;Acc:MGI:1933366]	protein_coding	-1.556	-1.642	497	133	115	**1.32e-14**	**3.23e-15**	-5.5
ENSMUSG00000021493	Pdlim7	PDZ and LIM domain 7 [Source:MGI Symbol;Acc:MGI:1914649]	protein_coding	-1.55	-0.187	21457	6225	15919	**3.79e-26**	**7.15e-01**	75.91
ENSMUSG00000030353	Tead4	TEA domain family member 4 [Source:MGI Symbol;Acc:MGI:106907]	protein_coding	1.549	0.818	369	1123	615	**8.97e-20**	**7.37e-05**	66.09
ENSMUSG00000052688	Rab7b	RAB7B, member RAS oncogene family [Source:MGI Symbol;Acc:MGI:2442295]	protein_coding	1.532	-0.329	170	515	114	**4.97e-18**	**4.76e-01**	110.99
ENSMUSG00000039419	Cntnap2	contactin associated protein-like 2 [Source:MGI Symbol;Acc:MGI:1914047]	protein_coding	1.522	0.916	1119	3828	2078	**1.67e-11**	**1.01e-03**	59.93
ENSMUSG00000093962	Gm11756	predicted gene 11756 [Source:MGI Symbol;Acc:MGI:3702095]	protein_coding	1.505	0.147	6	42	7	**5.19e-07**	**9.31e-01**	95.97
ENSMUSG00000074178	Gm10638	predicted gene 10638 [Source:MGI Symbol;Acc:MGI:3704316]	protein_coding	-1.491	-0.781	190	40	82	**3.91e-08**	**4.63e-02**	29.54
ENSMUSG00000085980	Gm12408	predicted gene 12408 [Source:MGI Symbol;Acc:MGI:3649921]	lincRNA	1.485	0.695	1	25	8	**5.56e-07**	**NA**	69.65
ENSMUSG00000078771	Evi2a	ecotropic viral integration site 2a [Source:MGI Symbol;Acc:MGI:95458]	protein_coding	1.482	0.953	107	317	196	**1.27e-15**	**1.22e-05**	54.12
ENSMUSG00000035314	Gdpd5	glycerophosphodiester phosphodiesterase domain containing 5 [Source:MGI Symbol;Acc:MGI:2686926]	protein_coding	-1.474	-0.872	675	195	298	**1.19e-14**	**1.27e-04**	24.32
ENSMUSG00000056328	Myh1	myosin, heavy polypeptide 1, skeletal muscle, adult [Source:MGI Symbol;Acc:MGI:1339711]	protein_coding	1.47	-0.297	252333	880063	162532	**2.26e-08**	**7.42e-01**	109.24
ENSMUSG00000026904	Slc4a10	solute carrier family 4, sodium bicarbonate cotransporter-like, member 10 [Source:MGI Symbol;Acc:MGI:2150150]	protein_coding	-1.466	-1.003	97	17	30	**5.53e-07**	**6.40e-03**	16.92
ENSMUSG00000063714	Sp3os	trans-acting transcription factor 3, opposite strand [Source:MGI Symbol;Acc:MGI:1914936]	antisense	1.465	0.296	53	158	59	**3.09e-14**	**6.22e-01**	89.62
ENSMUSG00000100084	2310068J16Rik	RIKEN cDNA 2310068J16 gene [Source:MGI Symbol;Acc:MGI:1917531]	antisense	1.463	0.527	17	65	26	**3.62e-08**	**3.53e-01**	78.3
ENSMUSG00000058831	Opn1sw	opsin 1 (cone pigments), short-wave-sensitive (color blindness, tritan) [Source:MGI Symbol;Acc:MGI:99438]	protein_coding	1.453	0.578	8	41	16	**5.58e-07**	**3.28e-01**	75.52
ENSMUSG00000048154	Kmt2d	lysine (K)-specific methyltransferase 2D [Source:MGI Symbol;Acc:MGI:2682319]	protein_coding	1.451	1.263	6319	17176	14123	**1.12e-26**	**1.82e-19**	28.39
ENSMUSG00000052143	Gm9869	predicted gene 9869 [Source:MGI Symbol;Acc:MGI:3642719]	antisense	1.449	0.406	189	531	224	**3.01e-18**	**2.07e-01**	84.86
ENSMUSG00000061742	Slc22a12	solute carrier family 22 (organic anion/cation transporter), member 12 [Source:MGI Symbol;Acc:MGI:1195269]	protein_coding	1.447	1.397	4	46	42	**1.50e-06**	**2.45e-05**	12.58
ENSMUSG00000040562	Gstm2	glutathione S-transferase, mu 2 [Source:MGI Symbol;Acc:MGI:95861]	protein_coding	-1.442	-1.065	2741	743	938	**1.44e-10**	**4.14e-05**	12.71
ENSMUSG00000026679	Enkur	enkurin, TRPC channel interacting protein [Source:MGI Symbol;Acc:MGI:1918483]	protein_coding	1.433	0.396	14	60	20	**4.80e-07**	**6.27e-01**	84.61
ENSMUSG00000034593	Myo5a	myosin VA [Source:MGI Symbol;Acc:MGI:105976]	protein_coding	1.432	1.11	917	2793	2032	**7.67e-12**	**2.36e-06**	40.4
ENSMUSG00000031925	Maml2	mastermind like 2 (Drosophila) [Source:MGI Symbol;Acc:MGI:2389460]	protein_coding	1.428	0.856	292	850	501	**8.33e-14**	**2.42e-04**	59.06
ENSMUSG00000070305	Mpzl3	myelin protein zero-like 3 [Source:MGI Symbol;Acc:MGI:2442647]	protein_coding	1.419	1.001	340	1401	876	**3.68e-07**	**3.67e-03**	49.11
ENSMUSG00000050592	Fam78a	family with sequence similarity 78, member A [Source:MGI Symbol;Acc:MGI:2443569]	protein_coding	-1.418	-0.474	2242	689	1323	**1.93e-15**	**1.13e-01**	49.31
ENSMUSG00000087413	Gm11266	predicted gene 11266 [Source:MGI Symbol;Acc:MGI:3649705]	antisense	1.414	0.097	220	749	201	**7.10e-08**	**9.53e-01**	97.85
ENSMUSG00000034926	Dhcr24	24-dehydrocholesterol reductase [Source:MGI Symbol;Acc:MGI:1922004]	protein_coding	1.412	0.374	237	669	275	**1.46e-14**	**3.53e-01**	85.84
ENSMUSG00000030772	Dkk3	dickkopf WNT signaling pathway inhibitor 3 [Source:MGI Symbol;Acc:MGI:1354952]	protein_coding	-1.411	-1.586	1743	276	196	**4.05e-06**	**8.11e-07**	-6.38
ENSMUSG00000031394	Opn1mw	opsin 1 (cone pigments), medium-wave-sensitive (color blindness, deutan) [Source:MGI Symbol;Acc:MGI:1097692]	protein_coding	1.404	0.821	402	1103	651	**3.77e-15**	**1.53e-04**	60
ENSMUSG00000085779	Atcayos	ataxia, cerebellar, Cayman type, opposite strand [Source:MGI Symbol;Acc:MGI:1916928]	antisense	1.399	0.303	4229	11578	4651	**2.22e-16**	**4.73e-01**	89.15
ENSMUSG00000047139	Cd24a	CD24a antigen [Source:MGI Symbol;Acc:MGI:88323]	protein_coding	1.397	0.761	16104	41233	24237	**1.37e-30**	**2.41e-08**	63.57
ENSMUSG00000024215	Spdef	SAM pointed domain containing ets transcription factor [Source:MGI Symbol;Acc:MGI:1353422]	protein_coding	1.396	0.31	8	37	10	**2.98e-06**	**7.69e-01**	87.56
ENSMUSG00000041801	Phlda3	pleckstrin homology like domain, family A, member 3 [Source:MGI Symbol;Acc:MGI:1351485]	protein_coding	1.387	0.626	2830	7418	3944	**1.28e-20**	**9.35e-04**	71.83
ENSMUSG00000037681	Esyt3	extended synaptotagmin-like protein 3 [Source:MGI Symbol;Acc:MGI:1098699]	protein_coding	1.385	0.727	10	45	22	**1.86e-06**	**1.21e-01**	65.07
ENSMUSG00000025020	Slit1	slit homolog 1 (Drosophila) [Source:MGI Symbol;Acc:MGI:1315203]	protein_coding	1.378	0.386	117	329	138	**8.44e-12**	**4.07e-01**	84.6
ENSMUSG00000040706	Agmat	agmatine ureohydrolase (agmatinase) [Source:MGI Symbol;Acc:MGI:1923236]	protein_coding	1.366	0.083	13	54	12	**4.06e-06**	**9.66e-01**	97.74
ENSMUSG00000092408	Vhl-ps1	von Hippel-Lindau tumor suppressor-like, pseudogene 1 [Source:MGI Symbol;Acc:MGI:3051121]	processed_pseudogene	1.362	0.503	8	47	16	**7.04e-06**	**4.69e-01**	78.35
ENSMUSG00000035595	1600002K03Rik	RIKEN cDNA 1600002K03 gene [Source:MGI Symbol;Acc:MGI:1917020]	protein_coding	-1.361	-0.156	484	155	368	**3.54e-14**	**8.36e-01**	77.73
ENSMUSG00000057337	Chst3	carbohydrate (chondroitin 6/keratan) sulfotransferase 3 [Source:MGI Symbol;Acc:MGI:1858224]	protein_coding	1.359	-0.173	77	221	57	**2.09e-09**	**8.81e-01**	108.13
ENSMUSG00000090556	Olfr753-ps1	olfactory receptor 753, pseudogene 1 [Source:MGI Symbol;Acc:MGI:3030587]	polymorphic_pseudogene	1.357	0.597	16	74	31	**5.06e-06**	**2.92e-01**	73.17
ENSMUSG00000085772	D630024D03Rik	RIKEN cDNA D630024D03 gene [Source:MGI Symbol;Acc:MGI:3041224]	lincRNA	1.355	0.109	59	195	56	**4.52e-07**	**9.48e-01**	96.93
ENSMUSG00000084819	Gm11967	predicted gene 11967 [Source:MGI Symbol;Acc:MGI:3650297]	antisense	1.35	0.596	69	191	99	**1.38e-11**	**5.50e-02**	72.06
ENSMUSG00000023047	Amhr2	anti-Mullerian hormone type 2 receptor [Source:MGI Symbol;Acc:MGI:105062]	protein_coding	1.337	0.478	140	395	180	**7.19e-10**	**2.51e-01**	79.05
ENSMUSG00000022454	Nell2	NEL-like 2 [Source:MGI Symbol;Acc:MGI:1858510]	protein_coding	1.327	0.65	12	56	26	**1.06e-05**	**2.24e-01**	68.34
ENSMUSG00000036949	Slc39a12	solute carrier family 39 (zinc transporter), member 12 [Source:MGI Symbol;Acc:MGI:2139274]	protein_coding	1.327	-0.054	4	29	3	**1.26e-05**	**NA**	103.48
ENSMUSG00000084790	Gm15879	predicted gene 15879 [Source:MGI Symbol;Acc:MGI:3802012]	antisense	-1.318	0.083	122	23	117	**1.06e-05**	**9.66e-01**	106.39
ENSMUSG00000026893	Gca	grancalcin [Source:MGI Symbol;Acc:MGI:1918521]	protein_coding	1.316	0.512	194	473	247	**1.87e-22**	**6.90e-03**	76.37
ENSMUSG00000027544	Nfatc2	nuclear factor of activated T cells, cytoplasmic, calcineurin dependent 2 [Source:MGI Symbol;Acc:MGI:102463]	protein_coding	1.315	0.337	1553	4036	1732	**2.42e-13**	**4.16e-01**	86.4
ENSMUSG00000044060	Btbd8	BTB (POZ) domain containing 8 [Source:MGI Symbol;Acc:MGI:3646208]	protein_coding	1.315	0.163	270	705	267	**8.14e-12**	**8.54e-01**	94.81
ENSMUSG00000069372	Ctxn3	cortexin 3 [Source:MGI Symbol;Acc:MGI:3642816]	protein_coding	1.309	1.196	2900	9203	7757	**3.65e-07**	**3.08e-05**	22.04
ENSMUSG00000000305	Cdh4	cadherin 4 [Source:MGI Symbol;Acc:MGI:99218]	protein_coding	-1.308	-0.942	3533	1078	1349	**4.39e-08**	**1.03e-03**	14.06
ENSMUSG00000015709	Arnt2	aryl hydrocarbon receptor nuclear translocator 2 [Source:MGI Symbol;Acc:MGI:107188]	protein_coding	-1.304	-0.91	234	74	96	**9.07e-10**	**2.73e-04**	16.82
ENSMUSG00000074217	2210011C24Rik	RIKEN cDNA 2210011C24 gene [Source:MGI Symbol;Acc:MGI:1917384]	protein_coding	-1.293	-0.611	166	45	83	**1.63e-06**	**1.85e-01**	36.27
ENSMUSG00000032315	Cyp1a1	cytochrome P450, family 1, subfamily a, polypeptide 1 [Source:MGI Symbol;Acc:MGI:88588]	protein_coding	1.289	0.908	84	247	167	**2.03e-07**	**3.05e-03**	47.25
ENSMUSG00000060981	Hist1h4h	histone cluster 1, H4h [Source:MGI Symbol;Acc:MGI:2448427]	protein_coding	1.287	0.709	536	1349	817	**1.04e-12**	**1.95e-03**	62.43
ENSMUSG00000026090	2010300C02Rik	RIKEN cDNA 2010300C02 gene [Source:MGI Symbol;Acc:MGI:1919347]	protein_coding	1.285	1.306	49	150	140	**7.29e-07**	**3.16e-06**	7.2
ENSMUSG00000056598	Drc3	dynein regulatory complex subunit 3 [Source:MGI Symbol;Acc:MGI:1921915]	protein_coding	1.282	0.629	63	160	91	**7.67e-12**	**1.99e-02**	67.55
ENSMUSG00000040424	Hipk4	homeodomain interacting protein kinase 4 [Source:MGI Symbol;Acc:MGI:2685008]	protein_coding	-1.275	-0.304	396	139	276	**1.93e-15**	**3.78e-01**	61.53

ENSMUSG00000086134	Gm16159	predicted gene 16159 [Source:MGI Symbol;Acc:MGI:3801949]	antisense	1.271	0.758	40	119	72	**4.50e-07**	**3.34e-02**	58.71
ENSMUSG00000039372	March4	membrane-associated ring finger (C3HC4) 4 [Source:MGI Symbol;Acc:MGI:2683550]	protein_coding	-1.27	-0.818	47	7	14	**3.80e-05**	**6.67e-02**	22.23
ENSMUSG00000062184	Hs6st2	heparan sulfate 6-O-sulfotransferase 2 [Source:MGI Symbol;Acc:MGI:1354959]	protein_coding	1.27	0.452	328	901	414	**3.77e-08**	**3.46e-01**	79.23
ENSMUSG00000045994	B3gat1	beta-1,3-glucuronyltransferase 1 (glucuronosyltransferase P) [Source:MGI Symbol;Acc:MGI:1924148]	protein_coding	1.268	1.1	22	93	72	**2.29e-05**	**1.72e-03**	26.79
ENSMUSG00000024697	Gna14	guanine nucleotide binding protein, alpha 14 [Source:MGI Symbol;Acc:MGI:95769]	protein_coding	1.266	0.747	39	139	79	**1.68e-05**	**9.56e-02**	59.3
ENSMUSG00000035849	Krt222	keratin 222 [Source:MGI Symbol;Acc:MGI:2442728]	protein_coding	1.266	0.172	278	674	276	**7.32e-14**	**8.00e-01**	94.02
ENSMUSG00000092035	Peg10	paternally expressed 10 [Source:MGI Symbol;Acc:MGI:2157785]	protein_coding	1.266	-0.209	157	396	116	**5.08e-11**	**7.78e-01**	110.51
ENSMUSG00000034623	Prss55	protease, serine 55 [Source:MGI Symbol;Acc:MGI:1918287]	protein_coding	1.26	0.093	46	129	43	**3.36e-07**	**9.53e-01**	97.32
ENSMUSG00000056370	Sftpb	surfactant associated protein B [Source:MGI Symbol;Acc:MGI:109516]	protein_coding	1.257	0.242	2	20	4	**2.94e-05**	**NA**	89.1
ENSMUSG00000017718	Afmid	arylformamidase [Source:MGI Symbol;Acc:MGI:2448704]	protein_coding	1.255	0.195	81	199	82	**5.29e-12**	**7.91e-01**	93.01
ENSMUSG00000035020	Epgn	epithelial mitogen [Source:MGI Symbol;Acc:MGI:1919170]	protein_coding	1.255	0.005	10	45	9	**4.84e-05**	**9.98e-01**	101.41
ENSMUSG00000030337	Vamp1	vesicle-associated membrane protein 1 [Source:MGI Symbol;Acc:MGI:1313276]	protein_coding	-1.253	-0.253	2447	891	1773	**1.24e-16**	**5.19e-01**	65.93
ENSMUSG00000061143	Maml3	mastermind like 3 (Drosophila) [Source:MGI Symbol;Acc:MGI:2389461]	protein_coding	1.251	0.197	300	717	303	**4.28e-15**	**7.28e-01**	92.89
ENSMUSG00000049985	Ankrd55	ankyrin repeat domain 55 [Source:MGI Symbol;Acc:MGI:1924568]	protein_coding	1.25	0.427	10	40	16	**3.45e-05**	**5.94e-01**	79.56
ENSMUSG00000050675	Gp1ba	glycoprotein 1b, alpha polypeptide [Source:MGI Symbol;Acc:MGI:1333744]	protein_coding	-1.25	-0.175	209	71	159	**2.17e-11**	**8.02e-01**	74.18
ENSMUSG00000086554	9530034E10Rik	RIKEN cDNA 9530034E10 gene [Source:MGI Symbol;Acc:MGI:1925865]	antisense	1.25	-0.041	3	24	2	**3.87e-05**	**NA**	103.35
ENSMUSG00000020331	Hcn2	hyperpolarization-activated, cyclic nucleotide-gated K+ 2 [Source:MGI Symbol;Acc:MGI:1298210]	protein_coding	1.249	0.547	94	277	137	**1.23e-06**	**2.49e-01**	72.6
ENSMUSG00000035916	Ptprq	protein tyrosine phosphatase, receptor type, Q [Source:MGI Symbol;Acc:MGI:1096349]	protein_coding	1.249	0.465	44	130	60	**2.54e-06**	**4.37e-01**	77.55
ENSMUSG00000050147	F2rl3	coagulation factor II (thrombin) receptor-like 3 [Source:MGI Symbol;Acc:MGI:1298207]	protein_coding	-1.246	0.001	152	45	131	**1.01e-06**	**9.99e-01**	94.84
ENSMUSG00000081855	Rpl17-ps5	ribosomal protein L17, pseudogene 5 [Source:MGI Symbol;Acc:MGI:3704246]	processed_pseudogene	-1.244	-0.341	907	222	555	**1.55e-05**	**7.02e-01**	57.07
ENSMUSG00000057762	Gm6169	predicted gene 6169 [Source:MGI Symbol;Acc:MGI:3646298]	protein_coding	1.241	0.829	112	264	182	**1.22e-15**	**5.49e-06**	50.26
ENSMUSG00000073374	C030034I22Rik	RIKEN cDNA C030034I22 gene [Source:MGI Symbol;Acc:MGI:1924783]	lincRNA	1.237	0.674	66	167	102	**6.96e-09**	**2.68e-02**	62.74
ENSMUSG00000024105	Themis3	thymocyte selection associated family member 3 [Source:MGI Symbol;Acc:MGI:1921806]	protein_coding	-1.235	-1.065	72	1	6	**4.44e-06**	**6.63e-04**	6.66
ENSMUSG00000035860	Cdhr3	cadherin-related family member 3 [Source:MGI Symbol;Acc:MGI:1916014]	protein_coding	1.233	0.449	29	84	40	**3.28e-06**	**4.71e-01**	77.95
ENSMUSG00000020181	Nav3	neuron navigator 3 [Source:MGI Symbol;Acc:MGI:2183703]	protein_coding	1.23	0.156	116	278	114	**1.40e-12**	**8.45e-01**	94.81
ENSMUSG00000021055	Esr2	estrogen receptor 2 (beta) [Source:MGI Symbol;Acc:MGI:109392]	protein_coding	1.23	1.146	5	33	28	**7.15e-05**	**1.30e-03**	17.71
ENSMUSG00000081929	Rps11-ps2	ribosomal protein S11, pseudogene 2 [Source:MGI Symbol;Acc:MGI:3651796]	processed_pseudogene	1.23	0.661	47	123	72	**6.52e-08**	**5.25e-02**	63
ENSMUSG00000035226	Rims4	regulating synaptic membrane exocytosis 4 [Source:MGI Symbol;Acc:MGI:2674366]	protein_coding	1.229	0.357	130	312	149	**3.77e-11**	**4.05e-01**	83.48
ENSMUSG00000022952	Runx1	runt related transcription factor 1 [Source:MGI Symbol;Acc:MGI:99852]	protein_coding	1.227	0.446	219	524	272	**5.08e-11**	**1.95e-01**	78.12
ENSMUSG00000025213	Kazald1	Kazal-type serine peptidase inhibitor domain 1 [Source:MGI Symbol;Acc:MGI:2147606]	protein_coding	-1.226	-0.998	263	84	96	**2.30e-07**	**2.73e-04**	8.29
ENSMUSG00000028885	Smpdl3b	sphingomyelin phosphodiesterase, acid-like 3B [Source:MGI Symbol;Acc:MGI:1916022]	protein_coding	1.224	0.458	43	114	56	**2.15e-07**	**3.62e-01**	77.03
ENSMUSG00000100210	Hist1h3f	histone cluster 1, H3f [Source:MGI Symbol;Acc:MGI:2448329]	protein_coding	1.222	0.324	23	67	28	**4.93e-06**	**7.09e-01**	85.31
ENSMUSG00000026435	Slc45a3	solute carrier family 45, member 3 [Source:MGI Symbol;Acc:MGI:1922082]	protein_coding	1.217	0.406	113	282	138	**2.65e-08**	**4.07e-01**	80.57
ENSMUSG00000027107	Chrna1	cholinergic receptor, nicotinic, alpha polypeptide 1 (muscle) [Source:MGI Symbol;Acc:MGI:87885]	protein_coding	1.216	0.401	1499	3836	1875	**7.90e-08**	**4.35e-01**	80.64
ENSMUSG00000023800	Tiam2	T cell lymphoma invasion and metastasis 2 [Source:MGI Symbol;Acc:MGI:1344338]	protein_coding	1.214	0.444	625	1556	799	**4.34e-08**	**3.22e-01**	77.95
ENSMUSG00000028417	Tal2	T cell acute lymphocytic leukemia 2 [Source:MGI Symbol;Acc:MGI:99540]	protein_coding	1.214	0.237	72	187	76	**5.44e-07**	**7.97e-01**	90
ENSMUSG00000052631	Sh2d6	SH2 domain containing 6 [Source:MGI Symbol;Acc:MGI:1918380]	protein_coding	1.213	0.257	242	701	277	**7.39e-06**	**7.96e-01**	89.11
ENSMUSG00000023039	Krt7	keratin 7 [Source:MGI Symbol;Acc:MGI:96704]	protein_coding	1.212	0.197	11	40	12	**6.91e-05**	**8.96e-01**	91.4
ENSMUSG00000081396	Gm15132	predicted gene 15132 [Source:MGI Symbol;Acc:MGI:3705367]	processed_pseudogene	1.211	0.346	3	23	6	**6.76e-05**	**NA**	82.07
ENSMUSG00000053719	Klk1b26	kallikrein 1-related petidase b26 [Source:MGI Symbol;Acc:MGI:891981]	protein_coding	1.21	1.06	35	158	130	**9.67e-05**	**3.96e-03**	25.38
ENSMUSG00000045776	Lrtm1	leucine-rich repeats and transmembrane domains 1 [Source:MGI Symbol;Acc:MGI:2442106]	protein_coding	-1.207	-0.641	3892	1377	1989	**6.61e-10**	**1.64e-02**	30.76
ENSMUSG00000056091	St3gal5	ST3 beta-galactoside alpha-2,3-sialyltransferase 5 [Source:MGI Symbol;Acc:MGI:1339963]	protein_coding	1.207	0.59	4799	11385	6756	**4.67e-10**	**3.59e-02**	67.92
ENSMUSG00000031628	Casp3	caspase 3 [Source:MGI Symbol;Acc:MGI:107739]	protein_coding	1.203	0.256	76	189	81	**4.21e-08**	**7.38e-01**	88.6
ENSMUSG00000054843	Atrnl1	attractin like 1 [Source:MGI Symbol;Acc:MGI:2147749]	protein_coding	1.203	0.459	919	2325	1210	**3.70e-08**	**2.72e-01**	76.8
ENSMUSG00000052336	Cx3cr1	chemokine (C-X3-C motif) receptor 1 [Source:MGI Symbol;Acc:MGI:1333815]	protein_coding	1.201	0.133	6	30	7	**1.23e-04**	**9.40e-01**	94.79
ENSMUSG00000055138	Gm4861	predicted gene 4861 [Source:MGI Symbol;Acc:MGI:3643777]	protein_coding	1.2	-0.003	9	34	7	**1.13e-04**	**9.99e-01**	102.16
ENSMUSG00000091561	Gm6665	predicted gene 6665 [Source:MGI Symbol;Acc:MGI:3643253]	protein_coding	-1.2	-1.065	117	35	38	**7.36e-06**	**5.37e-04**	3.63
ENSMUSG00000023043	Krt18	keratin 18 [Source:MGI Symbol;Acc:MGI:96692]	protein_coding	1.195	0.297	25	81	31	**4.96e-05**	**7.69e-01**	86.28
ENSMUSG00000025432	Avil	advillin [Source:MGI Symbol;Acc:MGI:1333798]	protein_coding	-1.191	-0.747	371	94	145	**7.20e-05**	**9.75e-02**	21.77
ENSMUSG00000028937	Acot7	acyl-CoA thioesterase 7 [Source:MGI Symbol;Acc:MGI:1917275]	protein_coding	-1.191	-0.232	1231	467	907	**1.86e-16**	**5.47e-01**	67.26
ENSMUSG00000095306	Gm21807	predicted gene, 21807 [Source:MGI Symbol;Acc:MGI:5433971]	pseudogene	1.191	0.482	40	124	60	**3.62e-05**	**4.54e-01**	75.68
ENSMUSG00000063388	BC023105	cDNA sequence BC023105 [Source:MGI Symbol;Acc:MGI:2384767]	pseudogene	-1.189	0.169	6148	1935	6186	**4.89e-06**	**8.96e-01**	118.23
ENSMUSG00000029205	Chrna9	cholinergic receptor, nicotinic, alpha polypeptide 9 [Source:MGI Symbol;Acc:MGI:1202403]	protein_coding	1.188	0.207	13	271	35	**2.67e-05**	**8.74e-01**	91.68
ENSMUSG00000029161	Cgref1	cell growth regulator with EF hand domain 1 [Source:MGI Symbol;Acc:MGI:1915817]	protein_coding	1.187	1.264	296	692	676	**1.11e-10**	**2.99e-11**	1.76
ENSMUSG00000032595	Cdhr4	cadherin-related family member 4 [Source:MGI Symbol;Acc:MGI:1916648]	protein_coding	1.181	0.309	10	47	15	**1.58e-04**	**7.69e-01**	85.04
ENSMUSG00000027654	Fam83d	family with sequence similarity 83, member D [Source:MGI Symbol;Acc:MGI:1919128]	protein_coding	-1.18	-0.759	326	81	119	**1.14e-04**	**9.67e-02**	20.9
ENSMUSG00000040852	Plekhh2	pleckstrin homology domain containing, family H (with MyTH4 domain) member 2 [Source:MGI Symbol;Acc:MGI:2146813]	protein_coding	1.18	0.088	145	343	135	**4.76e-09**	**9.45e-01**	97.78
ENSMUSG00000067597	Dgat2l6	diacylglycerol O-acyltransferase 2-like 6 [Source:MGI Symbol;Acc:MGI:3045268]	protein_coding	-1.178	0.058	101	28	92	**3.48e-05**	**9.72e-01**	102.51
ENSMUSG00000010136	Pifo	primary cilia formation [Source:MGI Symbol;Acc:MGI:1923670]	protein_coding	1.176	0.626	4	29	14	**1.53e-04**	**2.50e-01**	62.79
ENSMUSG00000003476	Crhr2	corticotropin releasing hormone receptor 2 [Source:MGI Symbol;Acc:MGI:894312]	protein_coding	-1.173	-0.662	2336	866	1224	**9.30e-11**	**4.18e-03**	27.57
ENSMUSG00000079499	6530402F18Rik	RIKEN cDNA 6530402F18 gene [Source:MGI Symbol;Acc:MGI:1923470]	processed_transcript	1.173	1.021	201	458	384	**2.43e-11**	**9.03e-08**	27.68
ENSMUSG00000036855	Gjd4	gap junction protein, delta 4 [Source:MGI Symbol;Acc:MGI:2444990]	protein_coding	1.171	0.11	1	23	2	**1.72e-05**	**NA**	95.82
ENSMUSG00000038244	Mical2	microtubule associated monooxygenase, calponin and LIM domain containing 2 [Source:MGI Symbol;Acc:MGI:2444947]	protein_coding	-1.171	-0.475	9742	3471	5758	**3.83e-09**	**1.71e-01**	43.02
ENSMUSG00000035513	Ntng2	netrin G2 [Source:MGI Symbol;Acc:MGI:2159341]	protein_coding	1.169	0.555	746	1630	969	**8.63e-17**	**2.25e-03**	68.61
ENSMUSG00000049866	Arl4c	ADP-ribosylation factor-like 4C [Source:MGI Symbol;Acc:MGI:2445172]	protein_coding	1.168	0.162	96	233	96	**6.93e-08**	**8.80e-01**	93.57
ENSMUSG00000097960	A330074K22Rik	RIKEN cDNA A330074K22 gene [Source:MGI Symbol;Acc:MGI:3045392]	lincRNA	1.168	0.037	171	462	155	**1.93e-05**	**9.81e-01**	100.3
ENSMUSG00000032750	Gab3	growth factor receptor bound protein 2-associated protein 3 [Source:MGI Symbol;Acc:MGI:2387324]	protein_coding	1.167	0.902	11	37	27	**7.47e-05**	**1.84e-02**	37.65

ENSMUSG00000024294	Mib1	mindbomb E3 ubiquitin protein ligase 1 [Source:MGI Symbol;Acc:MGI:2443157]	protein_coding	-1.165	-0.652	8255	1759	3286	**2.32e-04**	**2.33e-01**	27.43
ENSMUSG00000071637	Cebpd	CCAAT/enhancer binding protein (C/EBP), delta [Source:MGI Symbol;Acc:MGI:103573]	protein_coding	-1.163	0.08	1437	564	1330	**1.30e-18**	**9.17e-01**	104.47
ENSMUSG00000097125	Gm26885	predicted gene, 26885 [Source:MGI Symbol;Acc:MGI:5477379]	lincRNA	-1.162	-0.351	131	32	79	**1.51e-04**	**7.08e-01**	54.49
ENSMUSG00000021922	Itih4	inter alpha-trypsin inhibitor, heavy chain 4 [Source:MGI Symbol;Acc:MGI:109536]	protein_coding	1.161	-0.065	23	69	18	**8.43e-05**	**9.72e-01**	105.06
ENSMUSG00000011382	Dhdh	dihydrodiol dehydrogenase (dimeric) [Source:MGI Symbol;Acc:MGI:1919005]	protein_coding	-1.159	-0.263	4231	1657	3036	**4.49e-15**	**4.68e-01**	63.08
ENSMUSG00000027446	9230104L09Rik	RIKEN cDNA 9230104L09 gene [Source:MGI Symbol;Acc:MGI:1924955]	protein_coding	1.159	-0.042	17	53	14	**1.13e-04**	**9.81e-01**	103.59
ENSMUSG00000028950	Tas1r1	taste receptor, type 1, member 1 [Source:MGI Symbol;Acc:MGI:1927505]	protein_coding	1.157	0.033	166	414	148	**5.78e-07**	**9.81e-01**	100.4
ENSMUSG00000043230	Fam124b	family with sequence similarity 124, member B [Source:MGI Symbol;Acc:MGI:3026880]	protein_coding	1.149	0.183	15	46	16	**1.15e-04**	**9.05e-01**	91.8
ENSMUSG00000100181	RP23-69L13.3	RP23-69L13.3 (Clone-based (Vega))		1.148	0.512	14	45	22	**1.45e-04**	**4.34e-01**	71.16
ENSMUSG00000010044	Zmynd10	zinc finger, MYND domain containing 10 [Source:MGI Symbol;Acc:MGI:2387863]	protein_coding	-1.147	-0.108	57	14	44	**1.51e-04**	**9.52e-01**	81.83
ENSMUSG00000028621	Cyb5rl	cytochrome b5 reductase-like [Source:MGI Symbol;Acc:MGI:1919657]	protein_coding	-1.143	-0.403	583	231	374	**1.95e-18**	**3.32e-02**	49.38
ENSMUSG00000034648	Lrrn1	leucine rich repeat protein 1, neuronal [Source:MGI Symbol;Acc:MGI:106038]	protein_coding	1.143	0.534	745	1893	1077	**1.17e-05**	**2.62e-01**	69.37
ENSMUSG00000024786	Majin	membrane anchored junction protein [Source:MGI Symbol;Acc:MGI:1923913]	protein_coding	1.142	0.238	20	80	24	**2.87e-04**	**8.56e-01**	88.71
ENSMUSG00000032911	Cspg4	chondroitin sulfate proteoglycan 4 [Source:MGI Symbol;Acc:MGI:2153093]	protein_coding	1.141	0.196	865	1999	893	**7.85e-08**	**8.15e-01**	91.84
ENSMUSG00000029075	Tnfrsf4	tumor necrosis factor receptor superfamily, member 4 [Source:MGI Symbol;Acc:MGI:104512]	protein_coding	-1.139	0.098	160	56	148	**3.34e-07**	**9.38e-01**	107.5
ENSMUSG00000034452	Slc24a1	solute carrier family 24 (sodium/potassium/calcium exchanger), member 1 [Source:MGI Symbol;Acc:MGI:2384871]	protein_coding	1.139	0.177	15	56	17	**2.52e-04**	**9.12e-01**	92.45
ENSMUSG00000021983	Atp8a2	ATPase, aminophospholipid transporter-like, class I, type 8A, member 2 [Source:MGI Symbol;Acc:MGI:1354710]	protein_coding	1.134	0.288	257	721	294	**5.40e-05**	**7.63e-01**	86.16
ENSMUSG00000049939	Lrrc4	leucine rich repeat containing 4 [Source:MGI Symbol;Acc:MGI:2182081]	protein_coding	1.129	0.309	34	88	41	**1.09e-05**	**7.09e-01**	84.83
ENSMUSG00000072778	Vmn2r27	vomeronasal 2, receptor27 [Source:MGI Symbol;Acc:MGI:3761517]	protein_coding	1.129	0.612	1	14	6	**1.75e-04**	**NA**	60.38
ENSMUSG00000017412	Cacnb4	calcium channel, voltage-dependent, beta 4 subunit [Source:MGI Symbol;Acc:MGI:103301]	protein_coding	1.128	0.292	27	70	32	**1.72e-05**	**7.47e-01**	85.4
ENSMUSG00000053729	Spinkl	serine protease inhibitor, Kazal type-like [Source:MGI Symbol;Acc:MGI:1924674]	protein_coding	1.128	-0.032	14	44	11	**2.54e-04**	**9.86e-01**	103.81
ENSMUSG00000071855	Ccdc112	coiled-coil domain containing 112 [Source:MGI Symbol;Acc:MGI:1918800]	protein_coding	1.127	0.214	28	74	31	**2.10e-05**	**8.53e-01**	90.81
ENSMUSG00000097863	1010001B22Rik	RIKEN cDNA 1010001B22 gene [Source:MGI Symbol;Acc:MGI:1922653]	lincRNA	1.126	0.663	46	115	71	**5.47e-06**	**7.62e-02**	57.14
ENSMUSG00000024846	Cst6	cystatin E/M [Source:MGI Symbol;Acc:MGI:1920970]	protein_coding	-1.124	-0.176	926	348	692	**4.86e-08**	**8.36e-01**	72.45
ENSMUSG00000029607	Ankrd61	ankyrin repeat domain 61 [Source:MGI Symbol;Acc:MGI:1913979]	protein_coding	1.123	1.711	33	158	299	**3.70e-04**	**3.02e-08**	-97.4
ENSMUSG00000040010	Slc7a5	solute carrier family 7 (cationic amino acid transporter, y+ system), member 5 [Source:MGI Symbol;Acc:MGI:1298205]	protein_coding	-1.122	-0.616	457	158	241	**1.41e-05**	**1.36e-01**	29.33
ENSMUSG00000062542	Syt9	synaptotagmin IX [Source:MGI Symbol;Acc:MGI:1926373]	protein_coding	1.122	0.635	48	161	89	**2.39e-04**	**2.27e-01**	61.13
ENSMUSG00000036856	Wnt4	wingless-type MMTV integration site family, member 4 [Source:MGI Symbol;Acc:MGI:98957]	protein_coding	-1.12	-0.117	1564	574	1195	**4.16e-07**	**9.27e-01**	79.07
ENSMUSG00000007656	Arpp19	cAMP-regulated phosphoprotein 19 [Source:MGI Symbol;Acc:MGI:1891691]	protein_coding	1.119	0.417	4479	9223	5291	**1.86e-28**	**1.54e-03**	76.95
ENSMUSG00000021253	Tgfb3	transforming growth factor, beta 3 [Source:MGI Symbol;Acc:MGI:98727]	protein_coding	1.117	-0.065	2940	6098	2412	**1.33e-22**	**9.27e-01**	105.95
ENSMUSG00000042734	Ttc9	tetratricopeptide repeat domain 9 [Source:MGI Symbol;Acc:MGI:1916730]	protein_coding	1.115	0.368	482	2305	796	**4.95e-04**	**6.95e-01**	81.03
ENSMUSG00000037188	Grhl3	grainyhead-like 3 (Drosophila) [Source:MGI Symbol;Acc:MGI:2655333]	protein_coding	1.114	0.379	12	46	18	**3.94e-04**	**6.73e-01**	78.64
ENSMUSG00000086219	Srrm4os	serine/arginine repetitive matrix 4, opposite strand [Source:MGI Symbol;Acc:MGI:1924048]	antisense	-1.113	-0.154	107	17	72	**4.83e-04**	**9.29e-01**	75.4
ENSMUSG00000057230	Aak1	AP2 associated kinase 1 [Source:MGI Symbol;Acc:MGI:1098687]	protein_coding	1.111	0.379	388	857	462	**3.96e-09**	**3.34e-01**	79.83
ENSMUSG00000057913	Gm10032	predicted gene 10032 [Source:MGI Symbol;Acc:MGI:3642901]	protein_coding	1.11	0.237	17	77	22	**4.92e-04**	**8.58e-01**	88.9
ENSMUSG00000034584	Exph5	exophilin 5 [Source:MGI Symbol;Acc:MGI:2443248]	protein_coding	1.107	-0.46	38	132	16	**4.78e-04**	**5.41e-01**	119.53
ENSMUSG00000087018	2900072N19Rik	RIKEN cDNA 2900072N19 gene [Source:MGI Symbol;Acc:MGI:1920283]	lincRNA	-1.107	-0.725	34	4	10	**3.73e-04**	**1.32e-01**	22.26
ENSMUSG00000056423	Uts2b	urotensin 2B [Source:MGI Symbol;Acc:MGI:2677064]	protein_coding	-1.106	-0.807	77	16	24	**5.37e-04**	**7.62e-02**	15.31
ENSMUSG00000062661	Ncs1	neuronal calcium sensor 1 [Source:MGI Symbol;Acc:MGI:109166]	protein_coding	1.106	0.471	400	827	491	**3.90e-19**	**4.73e-03**	72.62
ENSMUSG00000102060	1700061E17Rik	RIKEN cDNA 1700061E17 gene [Source:MGI Symbol;Acc:MGI:3642196]	antisense	1.106	0.3	9	30	12	**4.34e-04**	**7.83e-01**	84.31
ENSMUSG00000020020	Usp44	ubiquitin specific peptidase 44 [Source:MGI Symbol;Acc:MGI:3045318]	protein_coding	1.105	0.256	11	37	14	**3.71e-04**	**8.30e-01**	86.24
ENSMUSG00000028782	Adgrb2	adhesion G protein-coupled receptor B2 [Source:MGI Symbol;Acc:MGI:2451244]	protein_coding	1.105	0.336	47	112	56	**3.38e-06**	**6.18e-01**	82.09
ENSMUSG00000097769	Snhg4	small nucleolar RNA host gene 4 [Source:MGI Symbol;Acc:MGI:4937091]	processed_transcript	1.105	0.575	107	236	147	**3.91e-09**	**3.65e-02**	64.17
ENSMUSG00000099760	Gm28800	predicted gene 28800 [Source:MGI Symbol;Acc:MGI:5579506]	antisense	1.105	0.09	15	51	15	**4.39e-04**	**9.64e-01**	97.25
ENSMUSG00000025887	Casp12	caspase 12 [Source:MGI Symbol;Acc:MGI:1312922]	protein_coding	-1.103	-0.996	1357	459	483	**5.09e-05**	**1.63e-03**	2.15
ENSMUSG00000025420	Katnal2	katanin p60 subunit A-like 2 [Source:MGI Symbol;Acc:MGI:1924234]	protein_coding	1.1	1.212	197	546	548	**7.70e-05**	**5.65e-05**	-4.91
ENSMUSG00000097666	A330094K24Rik	RIKEN cDNA A330094K24 gene [Source:MGI Symbol;Acc:MGI:1924343]	lincRNA	1.1	0.263	205	526	226	**3.54e-05**	**7.77e-01**	87.12
ENSMUSG00000024134	Six2	sine oculis-related homeobox 2 [Source:MGI Symbol;Acc:MGI:102778]	protein_coding	-1.099	-0.541	2867	1027	1564	**9.01e-06**	**2.12e-01**	34.74
ENSMUSG00000079428	Tceal7	transcription elongation factor A (SII)-like 7 [Source:MGI Symbol;Acc:MGI:1915746]	protein_coding	1.097	-0.622	1060	2585	502	**4.87e-05**	**1.58e-01**	126.07
ENSMUSG00000087579	1500017E21Rik	RIKEN cDNA 1500017E21 gene [Source:MGI Symbol;Acc:MGI:1919243]	processed_transcript	1.097	-0.208	412	1492	258	**6.67e-04**	**8.89e-01**	109.65
ENSMUSG00000035354	Uvrag	UV radiation resistance associated gene [Source:MGI Symbol;Acc:MGI:1925860]	protein_coding	1.096	0.264	734	1508	781	**1.36e-18**	**3.29e-01**	87.05
ENSMUSG00000031885	Cbfb	core binding factor beta [Source:MGI Symbol;Acc:MGI:99851]	protein_coding	-1.095	-0.603	12764	5287	7126	**9.05e-16**	**2.97e-04**	28.87
ENSMUSG00000001020	S100a4	S100 calcium binding protein A4 [Source:MGI Symbol;Acc:MGI:1330282]	protein_coding	1.088	-0.064	494	1056	402	**1.01e-09**	**9.56e-01**	105.72
ENSMUSG00000092403	Gm20497	predicted gene 20497 [Source:MGI Symbol;Acc:MGI:5141962]	pseudogene	1.088	0.361	20	106	37	**6.23e-04**	**7.02e-01**	80.5
ENSMUSG00000022847	Thpo	thrombopoietin [Source:MGI Symbol;Acc:MGI:101875]	protein_coding	1.086	0.624	354	752	502	**9.97e-11**	**3.82e-03**	59.35
ENSMUSG00000069273	Hist1h3e	histone cluster 1, H3e [Source:MGI Symbol;Acc:MGI:2448326]	protein_coding	1.086	0.524	13	42	22	**4.54e-04**	**4.19e-01**	66.88
ENSMUSG00000079164	Tlr5	toll-like receptor 5 [Source:MGI Symbol;Acc:MGI:1858171]	protein_coding	1.086	0.54	143	364	212	**6.21e-05**	**2.76e-01**	66.98
ENSMUSG00000015962	1700016C15Rik	RIKEN cDNA 1700016C15 gene [Source:MGI Symbol;Acc:MGI:1916678]	protein_coding	1.083	0.654	6	24	14	**6.48e-04**	**2.25e-01**	54.43
ENSMUSG00000035232	Pdk3	pyruvate dehydrogenase kinase, isoenzyme 3 [Source:MGI Symbol;Acc:MGI:2384308]	protein_coding	1.082	0.416	64	156	81	**1.65e-05**	**4.73e-01**	75.51
ENSMUSG00000070822	Zscan18	zinc finger and SCAN domain containing 18 [Source:MGI Symbol;Acc:MGI:3643810]	protein_coding	1.082	0.099	69	156	65	**3.54e-06**	**9.45e-01**	96.63
ENSMUSG00000053819	Camk2d	calcium/calmodulin-dependent protein kinase II, delta [Source:MGI Symbol;Acc:MGI:1341265]	protein_coding	1.081	0.183	3083	6292	3087	**3.75e-17**	**6.40e-01**	91.93
ENSMUSG00000090942	F830016B08Rik	RIKEN cDNA F830016B08 gene [Source:MGI Symbol;Acc:MGI:3588218]	protein_coding	-1.081	-0.454	657	229	375	**3.96e-05**	**4.19e-01**	41.52
ENSMUSG00000030825	Hsd17b14	hydroxysteroid (17-beta) dehydrogenase 14 [Source:MGI Symbol;Acc:MGI:1913315]	protein_coding	1.08	0.329	62	142	72	**2.85e-06**	**6.11e-01**	82.18
ENSMUSG00000027004	Frzb	frizzled-related protein [Source:MGI Symbol;Acc:MGI:892032]	protein_coding	1.077	-0.082	1273	2846	1042	**1.72e-06**	**9.53e-01**	106.68
ENSMUSG00000042155	Klhl23	kelch-like 23 [Source:MGI Symbol;Acc:MGI:2683536]	protein_coding	1.076	0.545	1577	3313	2105	**5.42e-11**	**1.57e-02**	65.7
ENSMUSG00000086003	B230206L02Rik	RIKEN cDNA B230206L02 gene [Source:MGI Symbol;Acc:MGI:1924598]	processed_transcript	1.076	0.213	130	279	134	**7.64e-09**	**7.48e-01**	90.04
ENSMUSG00000095742	CAAA01147332.1		protein_coding	-1.075	0.126	601	237	561	**9.73e-09**	**8.94e-01**	111.85
ENSMUSG00000030731	Syt3	synaptotagmin III [Source:MGI Symbol;Acc:MGI:99665]	protein_coding	-1.071	-0.536	474	181	258	**1.17e-06**	**1.31e-01**	34.08

ENSMUSG00000044033	Ccdc141	coiled-coil domain containing 141 [Source:MGI Symbol;Acc:MGI:1919735]	protein_coding	1.071	-0.047	296	634	247	**1.53e-07**	**9.72e-01**	105.19
ENSMUSG00000041852	Tcf20	transcription factor 20 [Source:MGI Symbol;Acc:MGI:108399]	protein_coding	1.069	0.203	949	2170	992	**4.24e-06**	**8.21e-01**	90.65
ENSMUSG00000028255	Clca1	chloride channel accessory 1 [Source:MGI Symbol;Acc:MGI:1346342]	protein_coding	1.067	0.347	5	51	14	**3.42e-04**	**6.88e-01**	80.45
ENSMUSG00000028399	Ptprd	protein tyrosine phosphatase, receptor type, D [Source:MGI Symbol;Acc:MGI:97812]	protein_coding	1.067	0.102	357	737	337	**4.70e-10**	**9.21e-01**	97.01
ENSMUSG00000078612	1700024P16Rik	RIKEN cDNA 1700024P16 gene [Source:MGI Symbol;Acc:MGI:2685466]	protein_coding	1.067	0.787	8	41	27	**8.35e-04**	**8.60e-02**	42.38
ENSMUSG00000025352	Gdf11	growth differentiation factor 11 [Source:MGI Symbol;Acc:MGI:1338027]	protein_coding	1.062	0.692	796	1900	1263	**2.54e-05**	**5.34e-02**	52.08
ENSMUSG00000062760	1810041L15Rik	RIKEN cDNA 1810041L15 gene [Source:MGI Symbol;Acc:MGI:1919551]	protein_coding	1.062	-0.107	38	97	28	**3.06e-04**	**9.52e-01**	107.26
ENSMUSG00000086915	Gm16365	predicted gene 16365 [Source:MGI Symbol;Acc:MGI:3840143]	antisense	1.062	0.285	87	186	95	**8.06e-08**	**6.22e-01**	84.28
ENSMUSG00000006205	Htra1	HtrA serine peptidase 1 [Source:MGI Symbol;Acc:MGI:1929076]	protein_coding	-1.061	-0.515	2836	1095	1614	**5.04e-06**	**2.07e-01**	35.31
ENSMUSG00000041923	Nol4	nucleolar protein 4 [Source:MGI Symbol;Acc:MGI:2441684]	protein_coding	1.061	0.631	13	41	24	**7.20e-04**	**2.41e-01**	55.94
ENSMUSG00000026117	Zap70	zeta-chain (TCR) associated protein kinase [Source:MGI Symbol;Acc:MGI:99613]	protein_coding	1.06	0.185	33	102	34	**7.86e-04**	**9.05e-01**	90.8
ENSMUSG00000051980	Casr	calcium-sensing receptor [Source:MGI Symbol;Acc:MGI:1351351]	protein_coding	-1.06	-1.456	803	329	228	**2.19e-09**	**2.38e-16**	-27.05
ENSMUSG00000087410	2310065F04Rik	RIKEN cDNA 2310065F04 gene [Source:MGI Symbol;Acc:MGI:1921434]	lincRNA	-1.06	0.137	8626	3675	8210	**3.46e-16**	**7.88e-01**	112.38
ENSMUSG00000026411	Tmem9	transmembrane protein 9 [Source:MGI Symbol;Acc:MGI:1913491]	protein_coding	1.059	0.213	296	624	299	**1.16e-07**	**7.68e-01**	89.86
ENSMUSG00000099364	5730419F03Rik	RIKEN cDNA 5730419F03 gene [Source:MGI Symbol;Acc:MGI:1917787]	lincRNA	1.059	0.873	102	234	191	**1.20e-05**	**2.59e-03**	32.72
ENSMUSG00000100680	1810044D09Rik	RIKEN cDNA 1810044D09 gene [Source:MGI Symbol;Acc:MGI:1917048]	lincRNA	1.059	0.435	88	196	110	**2.72e-06**	**3.50e-01**	73.8
ENSMUSG00000100855	Gm6976	predicted gene 6976	protein_coding	1.059	0.501	11	43	21	**9.78e-04**	**4.73e-01**	68.71
ENSMUSG00000024033	Rsph1	radial spoke head 1 homolog (Chlamydomonas) [Source:MGI Symbol;Acc:MGI:1194909]	protein_coding	1.058	-0.032	84	210	70	**1.48e-04**	**9.84e-01**	103.79
ENSMUSG00000060568	Fam78b	family with sequence similarity 78, member B [Source:MGI Symbol;Acc:MGI:2443050]	protein_coding	-1.058	-0.376	593	205	370	**8.27e-05**	**5.87e-01**	48.17
ENSMUSG00000018339	Gpx3	glutathione peroxidase 3 [Source:MGI Symbol;Acc:MGI:105102]	protein_coding	-1.057	-0.914	12250	4619	4709	**8.64e-06**	**9.43e-04**	3.63
ENSMUSG00000001870	Ltbp1	latent transforming growth factor beta binding protein 1 [Source:MGI Symbol;Acc:MGI:109151]	protein_coding	1.056	0.6	997	1999	1347	**1.95e-18**	**3.08e-05**	60.14
ENSMUSG00000074476	Spc24	SPC24, NDC80 kinetochore complex component, homolog (S. cerevisiae) [Source:MGI Symbol;Acc:MGI:1914879]	protein_coding	-1.056	-0.74	56	19	24	**1.82e-04**	**6.03e-02**	18.08
ENSMUSG00000047786	Lix1	limb and CNS expressed 1 [Source:MGI Symbol;Acc:MGI:1913893]	protein_coding	1.055	0.219	27	67	29	**1.19e-04**	**8.50e-01**	88.91
ENSMUSG00000018893	Mb	myoglobin [Source:MGI Symbol;Acc:MGI:96922]	protein_coding	1.054	0.221	139987	337771	150002	**1.54e-04**	**8.43e-01**	88.91
ENSMUSG00000022416	Cacna1i	calcium channel, voltage-dependent, alpha 1I subunit [Source:MGI Symbol;Acc:MGI:2178051]	protein_coding	1.053	1.323	25	120	163	**1.10e-03**	**1.04e-04**	-34.48
ENSMUSG00000010122	Slc47a1	solute carrier family 47, member 1 [Source:MGI Symbol;Acc:MGI:1914723]	protein_coding	1.052	0.568	1252	2834	1812	**2.57e-05**	**1.72e-01**	62.7
ENSMUSG00000052155	Acvr2a	activin receptor IIA [Source:MGI Symbol;Acc:MGI:102806]	protein_coding	-1.05	-0.979	3233	1279	1286	**3.09e-06**	**1.07e-04**	-1
ENSMUSG00000051985	Igfn1	immunoglobulin-like and fibronectin type III domain containing 1 [Source:MGI Symbol;Acc:MGI:3045352]	protein_coding	1.049	-0.699	3031	7140	1413	**4.53e-05**	**5.49e-02**	130.77
ENSMUSG00000053835	H2-T24	histocompatibility 2, T region locus 24 [Source:MGI Symbol;Acc:MGI:95958]	protein_coding	1.048	0.704	352	719	516	**3.86e-10**	**5.24e-04**	50.15
ENSMUSG00000079442	St6galnac4	ST6 (alpha-N-acetyl-neuraminyl-2,3-beta-galactosyl-1,3)-N-acetylgalactosaminide alpha-2,6-sialyltransferase 4 [Source:MGI Symbol;Acc:MGI:1341894]	protein_coding	1.048	0.019	1434	2930	1260	**3.17e-09**	**9.86e-01**	101.67
ENSMUSG00000033684	Qsox1	quiescin Q6 sulfhydryl oxidase 1 [Source:MGI Symbol;Acc:MGI:1330818]	protein_coding	1.046	0.58	1740	3431	2310	**4.45e-21**	**9.07e-06**	61.07
ENSMUSG00000097183	Gm17501	predicted gene, 17501 [Source:MGI Symbol;Acc:MGI:4937135]	lincRNA	1.046	0.33	46	108	55	**5.02e-05**	**6.59e-01**	81.43
ENSMUSG00000029563	Foxp2	forkhead box P2 [Source:MGI Symbol;Acc:MGI:2148705]	protein_coding	1.045	0.667	95	204	141	**2.40e-07**	**1.38e-02**	53.27
ENSMUSG00000022404	Slc25a17	solute carrier family 25 (mitochondrial carrier, peroxisomal membrane protein), member 17 [Source:MGI Symbol;Acc:MGI:1342248]	protein_coding	1.044	0.261	205	411	216	**7.73e-14**	**4.50e-01**	86
ENSMUSG00000027777	Schip1	schwannomin interacting protein 1 [Source:MGI Symbol;Acc:MGI:1353557]	protein_coding	1.044	0.579	4101	7944	5368	**6.99e-38**	**7.94e-11**	61.23
ENSMUSG00000072964	Bhlhb9	basic helix-loop-helix domain containing, class B9 [Source:MGI Symbol;Acc:MGI:1917487]	protein_coding	1.044	0.4	136	281	162	**2.36e-08**	**2.73e-01**	75.7
ENSMUSG00000101574	1700112H15Rik	RIKEN cDNA 1700112H15 gene [Source:MGI Symbol;Acc:MGI:1920840]	lincRNA	1.044	0.256	4	44	10	**4.60e-04**	**8.10e-01**	86.45
ENSMUSG00000044786	Zfp36	zinc finger protein 36 [Source:MGI Symbol;Acc:MGI:99180]	protein_coding	-1.042	-0.471	3472	1353	2101	**4.92e-06**	**2.60e-01**	38.54
ENSMUSG00000099465	Gm3830	predicted gene 3830 [Source:MGI Symbol;Acc:MGI:3782002]	lincRNA	1.042	-0.734	54	130	21	**2.53e-04**	**8.61e-02**	133.75
ENSMUSG00000099583	Hist1h3d	histone cluster 1, H3d [Source:MGI Symbol;Acc:MGI:2448322]	protein_coding	1.04	0.367	69	145	81	**1.85e-07**	**4.22e-01**	78.56
ENSMUSG00000026675	Hsd17b7	hydroxysteroid (17-beta) dehydrogenase 7 [Source:MGI Symbol;Acc:MGI:1330808]	protein_coding	-1.039	-0.673	1065	352	497	**5.46e-04**	**1.57e-01**	21.12
ENSMUSG00000038194	Lhb	luteinizing hormone beta [Source:MGI Symbol;Acc:MGI:96782]	processed_transcript	-1.038	-0.444	94	33	54	**1.22e-04**	**4.39e-01**	42.57
ENSMUSG00000052852	Reep1	receptor accessory protein 1 [Source:MGI Symbol;Acc:MGI:1098827]	protein_coding	-1.037	-0.666	8317	3613	4390	**2.48e-15**	**1.14e-05**	20.87
ENSMUSG00000066026	Dhrs3	dehydrogenase/reductase (SDR family) member 3 [Source:MGI Symbol;Acc:MGI:1315215]	protein_coding	-1.037	-0.155	1959	808	1486	**2.23e-08**	**8.44e-01**	73.06
ENSMUSG00000031221	Igbp1	immunoglobulin (CD79A) binding protein 1 [Source:MGI Symbol;Acc:MGI:1346500]	protein_coding	1.036	0.234	1869	3626	1910	**4.24e-26**	**2.25e-01**	88.01
ENSMUSG00000060579	Fhit	fragile histidine triad gene [Source:MGI Symbol;Acc:MGI:1277947]	protein_coding	1.036	0.216	44	96	46	**7.73e-06**	**8.13e-01**	88.88
ENSMUSG00000028197	Col24a1	collagen, type XXIV, alpha 1 [Source:MGI Symbol;Acc:MGI:1918605]	protein_coding	1.028	0.984	532	1182	1034	**1.83e-05**	**2.78e-04**	17.85
ENSMUSG00000042724	Map3k9	mitogen-activated protein kinase kinase kinase 9 [Source:MGI Symbol;Acc:MGI:2449952]	protein_coding	1.027	0.767	23	57	42	**2.03e-04**	**4.28e-02**	40.11
ENSMUSG00000051498	Tlr6	toll-like receptor 6 [Source:MGI Symbol;Acc:MGI:1341296]	protein_coding	1.027	0.027	76	159	68	**3.15e-07**	**9.82e-01**	101.52
ENSMUSG00000034765	Dusp5	dual specificity phosphatase 5 [Source:MGI Symbol;Acc:MGI:2685183]	protein_coding	1.025	0.254	158	321	168	**1.40e-08**	**6.34e-01**	86.22
ENSMUSG00000030465	Psd3	pleckstrin and Sec7 domain containing 3 [Source:MGI Symbol;Acc:MGI:1918215]	protein_coding	1.024	0.571	1828	3688	2467	**8.80e-11**	**5.25e-03**	61.13
ENSMUSG00000046733	Gprc5a	G protein-coupled receptor, family C, group 5, member A [Source:MGI Symbol;Acc:MGI:1891250]	protein_coding	-1.024	-0.292	189	75	129	**9.95e-06**	**6.56e-01**	57.33
ENSMUSG00000061959	Ces1e	carboxylesterase 1E [Source:MGI Symbol;Acc:MGI:95432]	protein_coding	1.024	-0.108	19	50	15	**5.51e-04**	**9.53e-01**	108.89
ENSMUSG00000083567	Gm11451	predicted gene 11451 [Source:MGI Symbol;Acc:MGI:3705412]	processed_pseudogene	1.023	0.301	102	211	112	**1.32e-07**	**5.61e-01**	82.45
ENSMUSG00000101086	Gm28651	predicted gene 28651 [Source:MGI Symbol;Acc:MGI:5579357]	lincRNA	1.022	0.113	157	332	151	**2.26e-06**	**9.27e-01**	95.68
ENSMUSG00000037553	Zdhhc18	zinc finger, DHHC domain containing 18 [Source:MGI Symbol;Acc:MGI:3527792]	protein_coding	-1.02	-0.102	2891	1244	2319	**3.70e-10**	**9.09e-01**	79.53
ENSMUSG00000056749	Nfil3	nuclear factor, interleukin 3, regulated [Source:MGI Symbol;Acc:MGI:109495]	protein_coding	-1.018	-0.367	3383	1379	2229	**6.35e-07**	**4.09e-01**	48.44
ENSMUSG00000031562	Dctd	dCMP deaminase [Source:MGI Symbol;Acc:MGI:2444529]	protein_coding	1.016	0.168	19	50	20	**7.57e-04**	**9.15e-01**	91.94
ENSMUSG00000087042	Gm11611	predicted gene 11611 [Source:MGI Symbol;Acc:MGI:3707011]	sense_intronic	-1.016	-0.168	76	25	57	**5.93e-04**	**9.09e-01**	72.45
ENSMUSG00000031146	Plp2	proteolipid protein 2 [Source:MGI Symbol;Acc:MGI:1298382]	protein_coding	1.014	0.645	1347	2562	1849	**1.02e-30**	**1.82e-11**	53.35
ENSMUSG00000039328	Rnf122	ring finger protein 122 [Source:MGI Symbol;Acc:MGI:1916117]	protein_coding	1.013	0.448	792	1579	950	**3.16e-09**	**1.05e-01**	71.33
ENSMUSG00000097027	Gm26559	predicted gene, 26559 [Source:MGI Symbol;Acc:MGI:5477053]	lincRNA	1.013	0.197	3	16	4	**1.29e-03**	**NA**	88.77
ENSMUSG00000037406	Htra4	HtrA serine peptidase 4 [Source:MGI Symbol;Acc:MGI:3036260]	protein_coding	-1.012	-0.92	277	28	37	**6.70e-04**	**1.13e-02**	4.04
ENSMUSG00000036617	Etl4	enhancer trap locus 4 [Source:MGI Symbol;Acc:MGI:95454]	protein_coding	1.01	0.433	5164	10201	6230	**2.72e-11**	**6.46e-02**	72.49
ENSMUSG00000038205	Prkab2	protein kinase, AMP-activated, beta 2 non-catalytic subunit [Source:MGI Symbol;Acc:MGI:1336185]	protein_coding	-1.01	-0.185	37524	16310	28362	**2.32e-10**	**7.37e-01**	68.53
ENSMUSG00000078915	Hsp25-ps1	heat shock protein 25, pseudogene 1 [Source:MGI Symbol;Acc:MGI:96241]	pseudogene	1.01	0.133	53	126	51	**3.90e-04**	**9.32e-01**	93.61

ENSMUSG00000097819	Gm26813	predicted gene, 26813 [Source:MGI Symbol;Acc:MGI:5477307]	lincRNA	1.009	-0.271	116	233	80	**1.98e-08**	**6.16e-01**	117.84
ENSMUSG00000038453	Srcin1	SRC kinase signaling inhibitor 1 [Source:MGI Symbol;Acc:MGI:1933179]	protein_coding	-1.007	-0.45	333	133	201	**3.46e-05**	**3.50e-01**	39.38
ENSMUSG00000040502	March9	membrane-associated ring finger (C3HC4) 9 [Source:MGI Symbol;Acc:MGI:2446144]	protein_coding	1.005	0.732	58	131	96	**5.34e-05**	**2.85e-02**	43.29
ENSMUSG00000027215	Cd82	CD82 antigen [Source:MGI Symbol;Acc:MGI:104651]	protein_coding	-1.003	-0.482	790	343	480	**1.23e-10**	**2.90e-02**	36.23

	gastrocnemius		*quadriceps*	
	Genes with CTG	All genes	Genes with CTG	All genes
Genes deregulated in HSA vs WT (FC>2)	17	376	27	588
Genes dergulated in HSA vs WT (FC>2) and corrected in pip6A-treated mice	15	322	20	495
Genes deregulated only in pip6a-treated mice vs WT (FC>2)	1	15	4	20

Supplementary table 2. Number of genes containing CTG tract (n ≥ 7) significantly deregulated in Pip6a-PMO-CAG7, HSA-LR and WT mice compared to all significantly deregulated genes.

Supplementary table 3: list and annotation of the most deregulated splicing events in the gastocnemius muscles of HSA-LR and pip6a-PMO treated mice compared to WT mice.

Table columns description:	
Ensembl Gene ID	*ENSEMBL gene identifier*
Name	*HGNC gene name*
Description	*Gene description*
Splice event	*Splice event*
Exon number	*Exon number defined using Fasterdb database*
Exon_bin	*Exon bin identifier*
Fold change (log2) WT vs. HSA-LR	*Fold change (log2) between WT and HSA-LR mice*
Fold Change (Log2) WT vs. Pip6a-PMO	*Fold change (log2) between WT and pip6a-PMO treated mice*
Gene type	*Gene type*
chr	*Chromosome number*
Strand	*Strand*
Start	*Exon bin start*
End	*Exon bin end*
Length	*Exon bin length*
EUC WT	*Exon Usage Coefficient (DEXseq) for WT*
EUC HSA-LR	*Exon Usage Coefficient (DEXseq) for HSA-LR*
EUC pip6a-PMO	*Exon Usage Coefficient (DEXseq) for pip6a-PMO treated*
Adj. p-value WT vs. HSA-LR	*Adjusted p-values for WT vs. HSA-LR*
Adj. p-value WT vs. Pip6a-PMO	*Adjusted p-value for WT vs. Pip6a-PMO*
Correction	*percentage of correction in pip6a-PMO treated compared to HSA-LR*

Splice events description:	
se	*Cassette Exon*
afe	*Alternative First Exon*
ale	*Alternative Last Exon*
ir	*Intron Retention*
mult	*Multiple Exons*
mxe	*Mutually exclusive Exons*
ss	*alternative Splice Site*

Ensembl Gene ID	Name	Description	Exon number	exon_bin	Fold change (log2) WT vs. HSA-LR	Fold Change (Log2) WT vs. Pip6a-PMO	Splice event	Gene type	Chr.	Strand	Start	End	Length	EUC WT	EUC HSA-LR	EUC pip6a-PMO	Adj.p-value	Correction (%)
ENSMUSG00000061723	Tnnt3	troponin T3, skeletal, fast [Source:MGI Symbol;Acc:MGI:109550]	8	E019	6.854	0.61	se	protein_coding	chr7	+	142 507 770	142 507 788	19	50.598	148.251	58.326	6.44e-61	92.1
ENSMUSG00000038056	Kmt2c	lysine (K)-specific methyltransferase 2C [Source:MGI Symbol;Acc:MGI:2444959]	30	E041	6.603	5.824	se	protein_coding	chr5	-	25 329 241	25 329 273	33	1.157	11.282	8.653	3.65e-14	26
ENSMUSG00000061723	Tnnt3	troponin T3, skeletal, fast [Source:MGI Symbol;Acc:MGI:109550]	8	E018	6.435	0.543	se	protein_coding	chr7	+	142 507 750	142 507 769	20	56.65	149.162	63.801	4.20e-30	92.3
ENSMUSG00000048154	Kmt2d	lysine (K)-specific methyltransferase 2D [Source:MGI Symbol;Acc:MGI:2682319]		E040	4.793	4.32	ss	protein_coding	chr15	-	98 856 069	98 856 077	9	1.514	7.929	6.74	6.04e-08	18.5
ENSMUSG00000021798	Ldb3	LIM domain binding 3 [Source:MGI Symbol;Acc:MGI:1344412]	11	E007	4.241	0.872	se	protein_coding	chr14	-	34 555 343	34 555 528	186	25.112	73.637	32.692	1.45e-43	84.4
ENSMUSG00000026489	Coq8a	coenzyme Q8A [Source:MGI Symbol;Acc:MGI:1914676]		E033	3.993	-0.001	ale	protein_coding	chr1	-	180 179 500	180 179 592	93	5.013	19.382	5.012	1.80e-23	100
ENSMUSG00000028964	Park7	Parkinson disease (autosomal recessive, early onset) 7 [Source:MGI Symbol;Acc:MGI:2135637]		E013	3.965	0.694	ale	protein_coding	chr4	-	150 905 126	150 905 249	124	5.256	20.081	6.675	2.54e-17	90.4
ENSMUSG00000069601	Ank3	ankyrin 3, epithelial [Source:MGI Symbol;Acc:MGI:88026]	32	E067	3.687	0.429	se	protein_coding	chr10	+	69 953 440	69 953 472	33	2.916	10.375	3.383	2.07e-19	93.7
ENSMUSG00000029642	Polr1d	polymerase (RNA) I polypeptide D [Source:MGI Symbol;Acc:MGI:108403]	3	E008	3.6	3.498	se	protein_coding	chr5	+	147 101 300	147 101 303	4	8.6	28.093	27.231	2.14e-02	4.4
ENSMUSG00000057897	Camk2b	calcium/calmodulin-dependent protein kinase II, beta [Source:MGI Symbol;Acc:MGI:88257]	16	E022	3.468	1.05	mxe	protein_coding	chr11	-	5 982 759	5 982 761	3	13.871	40.609	19.616	2.34e-29	78.5
ENSMUSG00000029862	Clcn1	chloride channel, voltage-sensitive 1 [Source:MGI Symbol;Acc:MGI:88417]	7	E010	3.417	0.472	se	protein_coding	chr6	+	42 293 444	42 293 522	79	11.189	33.481	13.121	4.23e-49	91.3
ENSMUSG00000024381	Bin1	bridging integrator 1 [Source:MGI Symbol;Acc:MGI:108092]	7	E007	3.226	0.43	se	protein_coding	chr18	+	32 414 945	32 415 037	93	14.309	38.992	16.509	1.41e-38	91.1
ENSMUSG00000034055	Phka1	phosphorylase kinase alpha 1 [Source:MGI Symbol;Acc:MGI:97576]	19	E017	-3.148	-0.482	se	protein_coding	chrX	-	102 557 096	102 557 272	177	61.227	26.565	54.964	6.17e-88	81.9
ENSMUSG00000062908	Acadm	acyl-Coenzyme A dehydrogenase, medium chain [Source:MGI Symbol;Acc:MGI:87867]		E017	3.128	1.369	afe	protein_coding	chr3	-	153 937 784	153 937 809	26	5.677	16.43	9.082	8.08e-30	68.3
ENSMUSG00000049225	Pdp1	pyruvate dehyrogenase phosphatase catalytic subunit 1 [Source:MGI Symbol;Acc:MGI:2685870]	2	E006	3.045	-0.313	afe	protein_coding	chr4	-	11 965 614	11 965 648	35	7.513	20.862	6.746	2.11e-99	105.7
ENSMUSG00000051747	Ttn	titin [Source:MGI Symbol;Acc:MGI:98864]	11	E379	-3.044	-0.922	se	protein_coding	chr2	-	76 965 777	76 965 914	138	21.573	7.789	15.969	5.40e-26	59.3
ENSMUSG00000028207	Asph	aspartate-beta-hydroxylase [Source:MGI Symbol;Acc:MGI:1914186]	7	E032	-2.961	-0.893	ir	protein_coding	chr4	-	9 630 547	9 630 925	379	35.339	13.903	27.127	4.88e-58	61.7
ENSMUSG00000028207	Asph	aspartate-beta-hydroxylase [Source:MGI Symbol;Acc:MGI:1914186]	7	E033	-2.931	-1.05	ir	protein_coding	chr4	-	9 630 926	9 630 952	27	29.323	11.332	21.136	1.04e-42	54.5
ENSMUSG00000008140	Emc10	ER membrane protein complex subunit 10 [Source:MGI Symbol;Acc:MGI:1916933]	8	E001	-2.805	-0.754	ale	protein_coding	chr7	-	44 489 937	44 490 110	174	23.286	9.178	18.266	2.07e-22	64.4
ENSMUSG00000027763	Mbnl1	muscleblind-like 1 (Drosophila) [Source:MGI Symbol;Acc:MGI:1928482]	7	E038	2.675	0.412	se	protein_coding	chr3	+	60 614 683	60 614 736	54	12.22	29.035	14.035	9.14e-26	89.2
ENSMUSG00000051747	Ttn	titin [Source:MGI Symbol;Acc:MGI:98864]	311	E017	2.649	1.494	mult	protein_coding	chr2	-	76 716 749	76 717 048	300	15.714	35.903	25.447	2.25e-03	51.8
ENSMUSG00000030592	Ryr1	ryanodine receptor 1, skeletal muscle [Source:MGI Symbol;Acc:MGI:99659]		E045	-2.639	-0.146	se	protein_coding	chr7	-	29 056 234	29 056 248	15	83.375	48.086	81.303	1.20e-76	94.1
ENSMUSG00000027601	Mtfr1	mitochondrial fission regulator 1 [Source:MGI Symbol;Acc:MGI:1914722]	5	E014	-2.634	-0.323	se	protein_coding	chr3	+	19 208 491	19 208 527	37	14.116	5.751	12.666	2.07e-11	82.7
ENSMUSG00000058925	1700011I03Rik	RIKEN cDNA 1700011I03 gene [Source:MGI Symbol;Acc:MGI:1922694]	1	E002	2.622	2.515	afe	protein_coding	chr18	+	57 533 829	57 533 881	53	4.127	10.16	9.795	1.99e-03	6
ENSMUSG00000051747	Ttn	titin [Source:MGI Symbol;Acc:MGI:98864]	312	E016	2.614	1.404	mult	protein_coding	chr2	-	76 715 540	76 716 115	576	24.53	51.089	37.252	2.20e-02	52.1
ENSMUSG00000021699	Pde4d	phosphodiesterase 4D, cAMP specific [Source:MGI Symbol;Acc:MGI:99555]		E026	2.571	-0.838	afe	protein_coding	chr13	+	109 757 221	109 757 270	50	6.236	14.948	4.672	8.24e-05	118
ENSMUSG00000076617	Ighm	immunoglobulin heavy constant mu [Source:MGI Symbol;Acc:MGI:96448]		E003	2.569	0.301	ale	IG_C_gene	chr12	-	113 420 741	113 420 928	188	24.955	51.209	27.387	9.56e-33	90.7
ENSMUSG00000061723	Tnnt3	troponin T3, skeletal, fast [Source:MGI Symbol;Acc:MGI:109550]	4	E010	2.526	0.061	se	protein_coding	chr7	+	142 502 740	142 502 757	18	113.043	150.397	113.935	3.70e-03	97.6
ENSMUSG00000051747	Ttn	titin [Source:MGI Symbol;Acc:MGI:98864]	313	E015	2.505	1.403	mult	protein_coding	chr2	-	76 715 144	76 715 449	306	25.921	51.931	39.109	7.30e-02	49.3
ENSMUSG00000051747	Ttn	titin [Source:MGI Symbol;Acc:MGI:98864]	310	E018	2.48	1.395	mult	protein_coding	chr2	-	76 717 173	76 717 478	306	14.519	31.918	22.898	1.11e-02	51.8
ENSMUSG00000026950	Neb	nebulin [Source:MGI Symbol;Acc:MGI:97292]	86	E091	2.429	1.432	mult	protein_coding	chr2	-	52 221 782	52 221 843	62	11.254	24.976	18.153	7.63e-03	49.7
ENSMUSG00000026950	Neb	nebulin [Source:MGI Symbol;Acc:MGI:97292]	85	E093	2.423	1.339	mult	protein_coding	chr2	-	52 222 901	52 223 048	148	11.456	25.348	17.915	8.43e-03	53.5
ENSMUSG00000026950	Neb	nebulin [Source:MGI Symbol;Acc:MGI:97292]	85	E092	2.411	1.367	mult	protein_coding	chr2	-	52 222 737	52 222 900	164	12.127	26.602	19.1	5.35e-03	51.8
ENSMUSG00000026950	Neb	nebulin [Source:MGI Symbol;Acc:MGI:97292]	83	E096	2.394	1.347	mult	protein_coding	chr2	-	52 225 569	52 225 673	105	10.357	22.875	16.284	1.28e-03	52.7
ENSMUSG00000026950	Neb	nebulin [Source:MGI Symbol;Acc:MGI:97292]		E090	2.372	1.392	mult	protein_coding	chr2	-	52 221 640	52 221 781	142	14.434	30.755	22.742	7.79e-03	49.1
ENSMUSG00000031865	Dctn1	dynactin 1 [Source:MGI Symbol;Acc:MGI:107745]	7	E013	2.37	-0.268	afe	protein_coding	chr6	+	83 186 428	83 186 445	18	8.605	19.061	7.852	1.76e-09	107.2
ENSMUSG00000049550	Clip1	CAP-GLY domain containing linker protein 1 [Source:MGI Symbol;Acc:MGI:1928401]		E013	2.366	0.019	se	protein_coding	chr5	-	123 581 592	123 581 597	6	15.324	32.376	15.419	2.22e-22	99.4
ENSMUSG00000061462	Obscn	obscurin, cytoskeletal calmodulin and titin-interacting RhoGEF [Source:MGI Symbol;Acc:MGI:2681862]	47	E082	2.321	1.328	mult	protein_coding	chr11	-	59 061 424	59 061 687	264	20.686	41.35	31.227	4.36e-07	49

ENSMUSG00000058925	1700011I03Rik	RIKEN cDNA 1700011I03 gene [Source:MGI Symbol;Acc:MGI:1922694]	1	E001	2.275	2.009	afe	protein_coding	chr18	+	57 533 780	57 533 828	49	4.127	9.026	8.242	2.43e-02	16
ENSMUSG00000061462	Obscn	obscurin, cytoskeletal calmodulin and titin-interacting RhoGEF [Source:MGI Symbol;Acc:MGI:2681862]	45	E084	2.275	1.326	mult	protein_coding	chr11	-	59 062 647	59 062 910	264	24.54	47	36.453	1.13e-05	47
ENSMUSG00000026950	Neb	nebulin [Source:MGI Symbol;Acc:MGI:97292]	84	E095	2.262	1.239	mult	protein_coding	chr2	-	52 224 514	52 224 621	108	11.241	23.68	17.016	3.12e-02	53.6
ENSMUSG00000026950	Neb	nebulin [Source:MGI Symbol;Acc:MGI:97292]		E087	2.25	1.272	mult	protein_coding	chr2	-	52 219 090	52 219 401	312	20.734	40.636	30.783	5.21e-02	49.5
ENSMUSG00000030730	Atp2a1	ATPase, Ca++ transporting, cardiac muscle, fast twitch 1 [Source:MGI Symbol;Acc:MGI:105058]	22	E003	-2.232	0.127	se	protein_coding	chr7	-	126 446 545	126 446 586	42	158.481	125.408	160.373	1.16e-78	105.7
ENSMUSG00000029478	Ncor2	nuclear receptor co-repressor 2 [Source:MGI Symbol;Acc:MGI:1337080]	48	E009	2.226	0.293	se	protein_coding	chr5	-	125 019 819	125 019 959	141	14.823	30.153	16.34	5.51e-34	90.1
ENSMUSG00000061723	Tnnt3	troponin T3, skeletal, fast [Source:MGI Symbol;Acc:MGI:109550]	2	E016	2.225	0.259	ir	protein_coding	chr7	+	142 505 300	142 505 491	192	45.67	74.658	48.753	6.70e-09	89.4
ENSMUSG00000026950	Neb	nebulin [Source:MGI Symbol;Acc:MGI:97292]		E088	2.22	1.235	mult	protein_coding	chr2	-	52 220 148	52 220 255	108	16.293	32.74	24.283	1.92e-02	51.4
ENSMUSG00000026950	Neb	nebulin [Source:MGI Symbol;Acc:MGI:97292]	82	E097	2.219	1.377	mult	protein_coding	chr2	-	52 226 443	52 226 646	204	11.914	24.654	18.835	4.60e-03	45.7
ENSMUSG00000026950	Neb	nebulin [Source:MGI Symbol;Acc:MGI:97292]		E089	2.185	1.236	mult	protein_coding	chr2	-	52 220 894	52 220 998	105	15.702	31.361	23.457	5.02e-02	50.5
ENSMUSG00000061462	Obscn	obscurin, cytoskeletal calmodulin and titin-interacting RhoGEF [Source:MGI Symbol;Acc:MGI:2681862]	48	E081	2.165	1.324	mult	protein_coding	chr11	-	59 060 844	59 061 107	264	26.856	49.167	39.502	9.46e-06	43.3
ENSMUSG00000061462	Obscn	obscurin, cytoskeletal calmodulin and titin-interacting RhoGEF [Source:MGI Symbol;Acc:MGI:2681862]	46	E083	2.164	1.167	mult	protein_coding	chr11	-	59 062 031	59 062 294	264	26.691	48.911	37.612	1.49e-04	50.8
ENSMUSG00000048154	Kmt2d	lysine (K)-specific methyltransferase 2D [Source:MGI Symbol;Acc:MGI:2682319]	22	E041	2.142	1.842	ss	protein_coding	chr15	-	98 856 078	98 856 182	105	8.897	18.266	16.552	1.36e-20	18.3
ENSMUSG00000027523	Gnas	GNAS (guanine nucleotide binding protein, alpha stimulating) complex locus [Source:MGI Symbol;Acc:MGI:95777]	10	E021	-2.096	-0.439	afe	protein_coding	chr2	+	174 330 089	174 330 096	8	10.138	4.939	8.729	2.60e-02	72.9
ENSMUSG00000048154	Kmt2d	lysine (K)-specific methyltransferase 2D [Source:MGI Symbol;Acc:MGI:2682319]	23	E039	2.068	1.711	mult	protein_coding	chr15	-	98 855 768	98 855 898	131	9.387	18.771	16.697	2.25e-22	22.1
ENSMUSG00000058925	1700011I03Rik	RIKEN cDNA 1700011I03 gene [Source:MGI Symbol;Acc:MGI:1922694]	1	E003	2.048	1.822	afe	protein_coding	chr18	+	57 533 882	57 533 968	87	6.294	12.66	11.729	1.92e-03	14.6
ENSMUSG00000030249	Abcc9	ATP-binding cassette, sub-family C (CFTR/MRP), member 9 [Source:MGI Symbol;Acc:MGI:1352630]	19	E025	2.034	0.258	se	protein_coding	chr6	-	142 648 027	142 648 065	39	13.486	26.102	14.702	7.14e-09	90.4
ENSMUSG00000031636	Pdlim3	PDZ and LIM domain 3 [Source:MGI Symbol;Acc:MGI:1859274]	4	E007	2.027	-0.44	se	protein_coding	chr8	+	45 908 469	45 908 656	188	22.481	40.971	19.52	4.06e-34	116
ENSMUSG00000048154	Kmt2d	lysine (K)-specific methyltransferase 2D [Source:MGI Symbol;Acc:MGI:2682319]	19	E045	2.024	1.811	mult	protein_coding	chr15	-	98 857 388	98 857 435	48	7.533	14.967	13.937	4.48e-16	13.9
ENSMUSG00000022139	Mbnl2	muscleblind-like 2 [Source:MGI Symbol;Acc:MGI:2145597]	9	E006	2.021	0.71	se	protein_coding	chr14	+	120 395 712	120 395 765	54	11.068	21.617	14.062	1.51e-31	71.6
ENSMUSG00000024969	Mark2	MAP/microtubule affinity regulating kinase 2 [Source:MGI Symbol;Acc:MGI:99638]	16	E020	1.953	-0.291	se	protein_coding	chr19	-	7 281 942	7 281 959	18	7.119	13.83	6.44	7.11e-22	110.1
ENSMUSG00000025871	4833439L19Rik	RIKEN cDNA 4833439L19 gene [Source:MGI Symbol;Acc:MGI:1921162]	2	E016	1.952	0.14	me	protein_coding	chr13	-	54 564 515	54 564 621	107	22.662	40.419	23.691	2.31e-79	94.2
ENSMUSG00000024294	Mib1	mindbomb E3 ubiquitin protein ligase 1 [Source:MGI Symbol;Acc:MGI:2443157]	8	E013	1.908	1.057	afe	protein_coding	chr18	+	10 760 804	10 760 808	5	5.533	10.641	7.957	7.99e-02	52.6
ENSMUSG00000061723	Tnnt3	troponin T3, skeletal, fast [Source:MGI Symbol;Acc:MGI:109550]	7	E017	1.905	-0.083	ir	protein_coding	chr7	+	142 505 516	142 505 530	15	115.786	143.956	114.57	3.18e-04	104.3
ENSMUSG00000048154	Kmt2d	lysine (K)-specific methyltransferase 2D [Source:MGI Symbol;Acc:MGI:2682319]	28	E032	1.886	1.404	se	protein_coding	chr15	-	98 854 531	98 854 615	85	10.806	20.236	17.293	1.49e-29	31.2
ENSMUSG00000057897	Camk2b	calcium/calmodulin-dependent protein kinase II, beta [Source:MGI Symbol;Acc:MGI:88257]	15	E024	-1.88	-0.151	mxe	protein_coding	chr11	-	5 987 057	5 987 131	75	48.628	28.903	46.822	3.28e-70	90.8
ENSMUSG00000025147	Mob2	MOB kinase activator 2 [Source:MGI Symbol;Acc:MGI:1919891]	3	E016	-1.872	-0.822	se	protein_coding	chr7	-	142 025 952	142 025 957	6	8.361	4.39	6.306	5.23e-04	48.3
ENSMUSG00000096054	Syne1	spectrin repeat containing, nuclear envelope 1 [Source:MGI Symbol;Acc:MGI:1927152]		E037	1.868	0.641	afe	protein_coding	chr10	-	5 117 100	5 117 122	23	6.587	12.459	8.208	2.43e-13	72.4
ENSMUSG00000031924	Cyb5b	cytochrome b5 type B [Source:MGI Symbol;Acc:MGI:1913677]	2	E002	1.858	0.86	afe	protein_coding	chr8	+	107 165 915	107 165 972	58	6.59	12.421	8.85	3.16e-02	61.2
ENSMUSG00000048154	Kmt2d	lysine (K)-specific methyltransferase 2D [Source:MGI Symbol;Acc:MGI:2682319]	26	E034	1.856	1.443	mult	protein_coding	chr15	-	98 855 025	98 855 135	111	10.068	18.731	16.355	8.71e-24	27.4
ENSMUSG00000048154	Kmt2d	lysine (K)-specific methyltransferase 2D [Source:MGI Symbol;Acc:MGI:2682319]	24	E036	1.856	1.413	mult	protein_coding	chr15	-	98 855 535	98 855 681	147	10.286	19.117	16.532	2.46e-24	29.3
ENSMUSG00000045962	Wnk1	WNK lysine deficient protein kinase 1 [Source:MGI Symbol;Acc:MGI:2442092]	4	E076	1.846	0.913	afe	protein_coding	chr6	-	119 990 168	119 990 249	82	10.436	19.324	14.202	2.27e-02	57.6
ENSMUSG00000045962	Wnk1	WNK lysine deficient protein kinase 1 [Source:MGI Symbol;Acc:MGI:2442092]	4	E074	1.845	0.71	afe	protein_coding	chr6	-	119 990 092	119 990 126	35	9.263	17.225	11.789	1.27e-05	68.3
ENSMUSG00000028514	Usp24	ubiquitin specific peptidase 24 [Source:MGI Symbol;Acc:MGI:1919936]	20	E029	1.843	0.528	se	protein_coding	chr4	+	106 373 991	106 374 044	54	7.14	13.37	8.555	8.49e-02	77.3
ENSMUSG00000064302	Clasp1	CLIP associating protein 1 [Source:MGI Symbol;Acc:MGI:1923957]	20	E045	-1.839	-0.069	afe	protein_coding	chr1	+	118 512 175	118 512 222	48	16.834	9.063	16.456	1.09e-17	95.1
ENSMUSG00000018501	Ncor1	nuclear receptor co-repressor 1 [Source:MGI Symbol;Acc:MGI:1349717]	20	E062	1.837	1.209	ss	protein_coding	chr11	-	62 378 497	62 378 514	18	4.765	8.959	7.226	2.65e-02	41.3
ENSMUSG00000048154	Kmt2d	lysine (K)-specific methyltransferase 2D [Source:MGI Symbol;Acc:MGI:2682319]	23	E038	1.836	1.399	mult	protein_coding	chr15	-	98 855 764	98 855 767	4	7.072	13.214	11.401	5.74e-11	29.5
ENSMUSG00000048154	Kmt2d	lysine (K)-specific methyltransferase 2D [Source:MGI Symbol;Acc:MGI:2682319]	21	E042	1.833	1.501	afe	protein_coding	chr15	-	98 856 270	98 856 309	40	8.896	16.501	14.782	2.81e-14	22.6
ENSMUSG00000034377	Tulp4	tubby like protein 4 [Source:MGI Symbol;Acc:MGI:1916092]	5	E020	1.826	1.751	afe	protein_coding	chr17	+	6 138 790	6 138 792	3	4.646	8.706	8.485	6.23e-03	5.4
ENSMUSG00000048154	Kmt2d	lysine (K)-specific methyltransferase 2D [Source:MGI Symbol;Acc:MGI:2682319]	29	E031	1.817	1.448	mult	protein_coding	chr15	-	98 854 183	98 854 424	242	13.489	24.404	21.709	3.70e-34	24.7
ENSMUSG00000061723	Tnnt3	troponin T3, skeletal, fast [Source:MGI Symbol;Acc:MGI:109550]		E021	1.814	0.23	se	protein_coding	chr7	+	142 507 845	142 507 863	19	38.455	60.265	40.945	8.59e-10	88.6
ENSMUSG00000056602	Fry	FRY microtubule binding protein [Source:MGI Symbol;Acc:MGI:2443895]	7	E016	1.809	0.654	ale	protein_coding	chr5	+	150 345 925	150 345 930	6	4.199	7.828	5.263	3.76e-04	70.7
ENSMUSG00000045962	Wnk1	WNK lysine deficient protein kinase 1 [Source:MGI Symbol;Acc:MGI:2442092]	7	E070	1.808	0.858	ale	protein_coding	chr6	-	119 972 768	119 972 856	89	10.738	19.61	14.34	2.23e-03	59.4
ENSMUSG00000022890	Atp5j	ATP synthase, H+ transporting, mitochondrial F0 complex, subunit F [Source:MGI Symbol;Acc:MGI:107777]		E027	-1.802	0.014	afe	protein_coding	chr16	-	84 835 603	84 835 625	23	9.022	4.857	9.064	5.54e-02	101
ENSMUSG00000045962	Wnk1	WNK lysine deficient protein kinase 1 [Source:MGI Symbol;Acc:MGI:2442092]	4	E075	1.788	0.82	afe	protein_coding	chr6	-	119 990 127	119 990 167	41	9.217	16.824	12.176	3.92e-04	61.1
ENSMUSG00000061462	Obscn	obscurin, cytoskeletal calmodulin and titin-interacting RhoGEF [Source:MGI Symbol;Acc:MGI:2681862]	49	E079	1.787	0.964	mult	protein_coding	chr11	-	59 055 961	59 056 224	264	33.036	53.031	43.172	5.13e-04	49.3
ENSMUSG00000048154	Kmt2d	lysine (K)-specific methyltransferase 2D [Source:MGI Symbol;Acc:MGI:2682319]	25	E035	1.784	1.386	mult	protein_coding	chr15	-	98 855 310	98 855 375	66	10.391	18.861	16.551	6.72e-24	27.3
ENSMUSG00000001036	Epn2	epsin 2 [Source:MGI Symbol;Acc:MGI:1333766]	4	E015	1.771	0.969	se	protein_coding	chr11	-	61 544 314	61 544 328	15	4.533	8.338	6.331	5.02e-04	52.8
ENSMUSG00000028207	Asph	aspartate-beta-hydroxylase [Source:MGI Symbol;Acc:MGI:1914186]	26	E008	1.769	-0.089	se	protein_coding	chr4	-	9 514 589	9 514 678	90	8.857	16.087	8.592	7.20e-25	103.7
ENSMUSG00000028906	Epb41	erythrocyte membrane protein band 4.1 [Source:MGI Symbol;Acc:MGI:95401]	21	E015	-1.766	-0.143	se	protein_coding	chr4	-	131 955 543	131 955 578	36	27.977	15.901	26.778	1.36e-60	90.1

ENSMUSG00000048154	Kmt2d	lysine (K)-specific methyltransferase 2D [Source:MGI Symbol;Acc:MGI:2682319]	21	E043	1.764	1.536	mult	protein_coding	chr15	-	98 856 310	98 856 389	80	9.726	17.582	16.311	1.17e-12	16.2
ENSMUSG00000035247	Hectd1	HECT domain containing 1 [Source:MGI Symbol;Acc:MGI:2384768]	7	E041	1.752	0.578	se	protein_coding	chr12	-	51 800 896	51 800 972	77	8.899	16.068	10.834	5.48e-03	73
ENSMUSG00000047921	Trappc9	trafficking protein particle complex 9 [Source:MGI Symbol;Acc:MGI:1923760]	3	E024	-1.744	-0.018	afe	protein_coding	chr15	-	73 055 748	73 055 812	65	19.783	11.072	19.668	7.68e-18	98.7
ENSMUSG00000061723	Tnnt3	troponin T3, skeletal, fast [Source:MGI Symbol;Acc:MGI:109550]		E020	1.742	0.185	se	protein_coding	chr7	+	142 507 789	142 507 807	19	37.785	58.497	39.762	3.79e-09	90.5
ENSMUSG00000020456	Ogdh	oxoglutarate (alpha-ketoglutarate) dehydrogenase (lipoamide) [Source:MGI Symbol;Acc:MGI:1098267]	8	E017	-1.712	-0.471	se	protein_coding	chr11	+	6 324 878	6 324 922	45	11.43	6.366	9.742	2.91e-02	66.7
ENSMUSG00000061462	Obscn	obscurin, cytoskeletal calmodulin and titin-interacting RhoGEF [Source:MGI Symbol;Acc:MGI:2681862]	51	E077	1.709	1.164	mult	protein_coding	chr11	-	59 054 774	59 055 037	264	26.337	43.056	37.135	8.53e-05	35.4
ENSMUSG00000057897	Camk2b	calcium/calmodulin-dependent protein kinase II, beta [Source:MGI Symbol;Acc:MGI:88257]	21	E016	-1.7	0.127	mult	protein_coding	chr11	-	5 977 812	5 977 940	129	37.467	22.487	38.808	1.19e-32	109
ENSMUSG00000048154	Kmt2d	lysine (K)-specific methyltransferase 2D [Source:MGI Symbol;Acc:MGI:2682319]	27	E033	1.683	1.213	mult	protein_coding	chr15	-	98 854 714	98 854 851	138	12.408	21.618	18.573	1.09e-21	33.1
ENSMUSG00000061462	Obscn	obscurin, cytoskeletal calmodulin and titin-interacting RhoGEF [Source:MGI Symbol;Acc:MGI:2681862]	114	E001	-1.681	-0.262	mult	protein_coding	chr11	-	58 994 256	58 994 258	3	7.366	4.127	6.731	1.40e-02	80.4
ENSMUSG00000048154	Kmt2d	lysine (K)-specific methyltransferase 2D [Source:MGI Symbol;Acc:MGI:2682319]	33	E027	1.661	1.498	mult	protein_coding	chr15	-	98 851 607	98 851 789	183	15.931	27.136	25.81	4.52e-22	11.8
ENSMUSG00000048154	Kmt2d	lysine (K)-specific methyltransferase 2D [Source:MGI Symbol;Acc:MGI:2682319]	30	E030	1.66	1.39	mult	protein_coding	chr15	-	98 854 033	98 854 106	74	11.107	19.31	17.678	1.84e-19	19.9
ENSMUSG00000049225	Pdp1	pyruvate dehyrogenase phosphatase catalytic subunit 1 [Source:MGI Symbol;Acc:MGI:2685870]		E008	1.653	0.108	ale	protein_coding	chr4	-	11 965 884	11 965 927	44	6.747	11.86	7.001	3.13e-04	95
ENSMUSG00000024456	Diaph1	diaphanous related formin 1 [Source:MGI Symbol;Acc:MGI:1194490]	1	E043	-1.647	0.054	afe	protein_coding	chr18	-	37 935 412	37 935 423	12	8.785	4.987	8.948	1.16e-05	104.3
ENSMUSG00000032578	Cish	cytokine inducible SH2-containing protein [Source:MGI Symbol;Acc:MGI:103159]		E008	-1.64	1.609	ss	protein_coding	chr9	+	107 299 896	107 299 923	28	7.355	4.18	12.723	5.58e-02	269.1
ENSMUSG00000057897	Camk2b	calcium/calmodulin-dependent protein kinase II, beta [Source:MGI Symbol;Acc:MGI:88257]	22	E015	-1.634	0.102	mult	protein_coding	chr11	-	5 976 765	5 976 893	129	34.667	21.037	35.689	3.10e-24	107.5
ENSMUSG00000057897	Camk2b	calcium/calmodulin-dependent protein kinase II, beta [Source:MGI Symbol;Acc:MGI:88257]	20	E017	-1.633	0.041	mult	protein_coding	chr11	-	5 978 399	5 978 512	114	41.117	25.552	41.566	1.94e-27	102.9
ENSMUSG00000027257	Pacsin3	protein kinase C and casein kinase substrate in neurons 3 [Source:MGI Symbol;Acc:MGI:1891410]	3	E012	1.63	0.605	afe	protein_coding	chr2	+	91 257 329	91 257 334	6	6.938	12.098	8.538	1.02e-08	69
ENSMUSG00000030987	Stim1	stromal interaction molecule 1 [Source:MGI Symbol;Acc:MGI:107476]		E021	1.624	0.304	se	protein_coding	chr7	+	102 428 774	102 428 866	93	19.328	32.027	21.322	8.68e-24	84.3
ENSMUSG00000087410	2310065F04Rik	RIKEN cDNA 2310065F04 gene [Source:MGI Symbol;Acc:MGI:1921434]		E007	1.621	-0.195	afe	lincRNA	chr11	-	67 119 757	67 119 914	158	17.055	28.554	15.988	6.36e-63	109.3
ENSMUSG00000029472	Anapc5	anaphase-promoting complex subunit 5 [Source:MGI Symbol;Acc:MGI:1929722]		E043	1.617	-0.46	afe	protein_coding	chr5	-	122 818 543	122 818 583	41	11.85	20.26	10.138	1.22e-22	120.4
ENSMUSG00000026950	Neb	nebulin [Source:MGI Symbol;Acc:MGI:97292]	80	E099	1.615	0.793	mult	protein_coding	chr2	-	52 228 498	52 228 605	108	10.507	18.04	13.733	2.12e-02	57.2
ENSMUSG00000024431	Nr3c1	nuclear receptor subfamily 3, group C, member 1 [Source:MGI Symbol;Acc:MGI:95824]	10	E002	-1.614	-0.786	ale	protein_coding	chr18	-	39 410 547	39 411 986	1440	74.467	52.92	63.696	5.12e-18	50
ENSMUSG00000020190	Mknk2	MAP kinase-interacting serine/threonine kinase 2 [Source:MGI Symbol;Acc:MGI:894279]		E020	1.613	-0.449		protein_coding	chr10	-	80 672 252	80 672 291	40	9.364	16.125	8.031	7.80e-11	119.7
ENSMUSG00000009090	Ap1b1	adaptor protein complex AP-1, beta 1 subunit [Source:MGI Symbol;Acc:MGI:1096368]	15	E021	1.606	-0.037	se	protein_coding	chr11	+	5 033 242	5 033 262	21	11.421	19.487	11.279	1.03e-15	101.8
ENSMUSG00000061462	Obscn	obscurin, cytoskeletal calmodulin and titin-interacting RhoGEF [Source:MGI Symbol;Acc:MGI:2681862]	50	E078	1.605	0.923	mult	protein_coding	chr11	-	59 055 377	59 055 640	264	30.172	47.05	39.314	2.98e-02	45.8
ENSMUSG00000031352	Hccs	holocytochrome c synthetase [Source:MGI Symbol;Acc:MGI:106911]	1	E014	-1.603	-0.518	afe	protein_coding	chrX	-	169 320 281	169 320 294	14	7.21	4.149	6.033	3.31e-02	61.5
ENSMUSG00000029993	Nfu1	NFU1 iron-sulfur cluster scaffold [Source:MGI Symbol;Acc:MGI:1913290]		E013	-1.6	-0.168	se	protein_coding	chr6	+	87 016 176	87 016 394	219	26.039	15.549	24.714	1.28e-06	87.4
ENSMUSG00000064302	Clasp1	CLIP associating protein 1 [Source:MGI Symbol;Acc:MGI:1923957]	28	E060	1.592	0.375	mxe	protein_coding	chr1	+	118 541 675	118 541 698	24	10.275	17.522	11.668	1.51e-23	80.8
ENSMUSG00000039844	Rapgef1	Rap guanine nucleotide exchange factor (GEF) 1 [Source:MGI Symbol;Acc:MGI:104580]	14	E019	-1.588	-0.069	se	protein_coding	chr2	+	29 720 643	29 720 738	96	34.662	21.35	33.979	1.08e-19	94.9
ENSMUSG00000087410	2310065F04Rik	RIKEN cDNA 2310065F04 gene [Source:MGI Symbol;Acc:MGI:1921434]		E008	1.581	-0.159	afe	lincRNA	chr11	-	67 120 047	67 120 080	34	13.817	23.17	13.101	2.00e-29	107.7
ENSMUSG00000045962	Wnk1	WNK lysine deficient protein kinase 1 [Source:MGI Symbol;Acc:MGI:2442092]	10	E064	1.579	0.813	afe	protein_coding	chr6	-	119 968 476	119 968 663	188	15.322	25.522	20.013	2.58e-02	54
ENSMUSG00000064302	Clasp1	CLIP associating protein 1 [Source:MGI Symbol;Acc:MGI:1923957]	26	E055	1.563	0.146	ir	protein_coding	chr1	+	118 536 135	118 536 197	63	8.217	13.962	8.637	8.57e-07	92.7
ENSMUSG00000048154	Kmt2d	lysine (K)-specific methyltransferase 2D [Source:MGI Symbol;Acc:MGI:2682319]	31	E029	1.557	1.194	mult	protein_coding	chr15	-	98 853 790	98 853 840	51	11.009	18.513	16.431	8.24e-19	27.8
ENSMUSG00000032578	Cish	cytokine inducible SH2-containing protein [Source:MGI Symbol;Acc:MGI:103159]		E006	-1.555	1.046	afe	protein_coding	chr9	+	107 299 155	107 299 213	59	7.477	4.376	10.687	7.74e-03	203.5
ENSMUSG00000045962	Wnk1	WNK lysine deficient protein kinase 1 [Source:MGI Symbol;Acc:MGI:2442092]	16	E050	1.554	0.681	se	protein_coding	chr6	-	119 952 869	119 952 969	101	13.038	21.741	16.366	2.38e-02	61.8
ENSMUSG00000033196	Myh2	myosin, heavy polypeptide 2, skeletal muscle, adult [Source:MGI Symbol;Acc:MGI:1339710]	24	E026	1.546	-0.089	ss	protein_coding	chr11	+	67 188 662	67 188 838	177	98.033	120.72	96.741	1.75e-20	105.7
ENSMUSG00000061723	Tnnt3	troponin T3, skeletal, fast [Source:MGI Symbol;Acc:MGI:109550]		E014	1.537	0.369	se	protein_coding	chr7	+	142 504 691	142 504 698	8	45.122	64.557	49.516	8.42e-06	77.4
ENSMUSG00000028619	Tceanc2	transcription elongation factor A (SII) N-terminal and central domain containing 2 [Source:MGI Symbol;Acc:MGI:1913776]		E013	1.535	0.71	ale	protein_coding	chr4	-	107 176 616	107 176 624	9	5.847	9.898	7.465	5.53e-03	60.1
ENSMUSG00000028207	Asph	aspartate-beta-hydroxylase [Source:MGI Symbol;Acc:MGI:1914186]	15	E021	1.529	0.039	mult	protein_coding	chr4	-	9 598 733	9 598 780	48	10.957	18.257	11.102	1.12e-11	98
ENSMUSG00000027827	Kcnab1	potassium voltage-gated channel, shaker-related subfamily, beta member 1 [Source:MGI Symbol;Acc:MGI:109155]	6	E006	1.526	0.02	se	protein_coding	chr3	+	65 266 491	65 266 534	44	6.74	11.351	6.786	1.03e-02	99
ENSMUSG00000033196	Myh2	myosin, heavy polypeptide 2, skeletal muscle, adult [Source:MGI Symbol;Acc:MGI:1339710]	23	E025	1.526	-0.076	ss	protein_coding	chr11	+	67 188 308	67 188 550	243	97.395	119.778	96.296	3.05e-19	104.9
ENSMUSG00000028207	Asph	aspartate-beta-hydroxylase [Source:MGI Symbol;Acc:MGI:1914186]	14	E022	1.525	0.135	mult	protein_coding	chr4	-	9 601 307	9 601 363	57	12.18	20.188	12.748	1.41e-06	92.9
ENSMUSG00000058925	1700011I03Rik	RIKEN cDNA 1700011I03 gene [Source:MGI Symbol;Acc:MGI:1922694]	2	E004	1.52	1.319	afe	protein_coding	chr18	+	57 538 033	57 538 084	52	8.217	13.762	12.864	9.60e-04	16.2
ENSMUSG00000038485	Socs7	suppressor of cytokine signaling 7 [Source:MGI Symbol;Acc:MGI:2651588]	4	E004	1.513	0.247	mxe	protein_coding	chr11	+	97 376 997	97 377 098	102	15.621	25.453	16.949	6.60e-05	86.5
ENSMUSG00000061462	Obscn	obscurin, cytoskeletal calmodulin and titin-interacting RhoGEF [Source:MGI Symbol;Acc:MGI:2681862]	52	E076	1.509	0.965	mult	protein_coding	chr11	-	59 054 162	59 054 425	264	27.042	41.78	35.965	5.84e-05	39.5
ENSMUSG00000030616	Sytl2	synaptotagmin-like 2 [Source:MGI Symbol;Acc:MGI:1933366]		E026	-1.507	-1.477	afe	protein_coding	chr7	+	90 387 359	90 387 371	13	5.766	3.427	3.462	5.30e-02	1.5
ENSMUSG00000061313	Ddhd2	DDHD domain containing 2 [Source:MGI Symbol;Acc:MGI:1919358]	1	E033	-1.505	-0.567	afe	protein_coding	chr8	-	25 754 267	25 754 280	14	7.975	4.751	6.565	6.48e-02	56.3

ENSMUSG00000027022	Xirp2	xin actin-binding repeat containing 2 [Source:MGI Symbol;Acc:MGI:2685198]	6	E006	1.499	0.059	se	protein_coding	chr2	+	67 507 047	67 507 180	134	16.376	26.486	16.699	1.22e-03	96.8
ENSMUSG00000020190	Mknk2	MAP kinase-interacting serine/threonine kinase 2 [Source:MGI Symbol;Acc:MGI:894279]		E019	1.498	-0.754	afe	protein_coding	chr10	-	80 672 170	80 672 251	82	10.88	17.951	8.413	2.68e-08	134.9
ENSMUSG00000093904	Tomm20	translocase of outer mitochondrial membrane 20 homolog (yeast) [Source:MGI Symbol;Acc:MGI:1915202]	1	E007	-1.495	-0.501	afe	protein_coding	chr8	-	126 945 841	126 945 844	4	8.268	4.944	6.962	2.01e-02	60.7
ENSMUSG00000048154	Kmt2d	lysine (K)-specific methyltransferase 2D [Source:MGI Symbol;Acc:MGI:2682319]	13	E051	1.494	1.308	mult	protein_coding	chr15	-	98 861 051	98 861 164	114	10.401	17.165	16.141	5.42e-08	15.1
ENSMUSG00000002107	Celf2	CUGBP, Elav-like family member 2 [Source:MGI Symbol;Acc:MGI:1338822]		E012	1.493	-0.651	ir	protein_coding	chr2	-	6 546 859	6 546 871	13	5.777	9.64	4.614	3.51e-08	130.1
ENSMUSG00000045962	Wnk1	WNK lysine deficient protein kinase 1 [Source:MGI Symbol;Acc:MGI:2442092]	13	E058	1.493	0.743	afe	protein_coding	chr6	-	119 960 644	119 960 724	81	14.073	22.928	18.004	4.11e-02	55.6
ENSMUSG00000023092	Fhl1	four and a half LIM domains 1 [Source:MGI Symbol;Acc:MGI:1298387]	1	E002	-1.48	0.109	afe	protein_coding	chrX	+	56 731 865	56 731 868	4	10	6.023	10.377	3.08e-02	109.5
ENSMUSG00000029642	Polr1d	polymerase (RNA) I polypeptide D [Source:MGI Symbol;Acc:MGI:108403]	3	E007	1.473	1.292	se	protein_coding	chr5	+	147 101 229	147 101 299	71	20.506	32.33	30.645	5.78e-02	14.3
ENSMUSG00000048154	Kmt2d	lysine (K)-specific methyltransferase 2D [Source:MGI Symbol;Acc:MGI:2682319]	14	E050	1.466	1.405	mult	protein_coding	chr15	-	98 860 634	98 860 744	111	9.845	16.123	15.804	2.04e-13	5.1
ENSMUSG00000039782	Cpeb2	cytoplasmic polyadenylation element binding protein 2 [Source:MGI Symbol;Acc:MGI:2442640]	7	E017	1.465	0.464	se	protein_coding	chr5	+	43 268 869	43 268 892	24	8.029	13.207	9.409	5.53e-06	73.3
ENSMUSG00000028207	Asph	aspartate-beta-hydroxylase [Source:MGI Symbol;Acc:MGI:1914186]	13	E023	1.464	0.143	mult	protein_coding	chr4	-	9 602 469	9 602 501	33	11.814	19.224	12.397	3.11e-04	92.1
ENSMUSG00000026309	Ilkap	integrin-linked kinase-associated serine/threonine phosphatase 2C [Source:MGI Symbol;Acc:MGI:1914694]	1	E037	-1.463	0.097	afe	protein_coding	chr1	-	91 398 779	91 398 783	5	6.63	4.003	6.854	4.31e-02	108.5
ENSMUSG00000032497	Lrrfip2	leucine rich repeat (in FLII) interacting protein 2 [Source:MGI Symbol;Acc:MGI:1918518]		E018	-1.462	-0.024	se	protein_coding	chr9	+	111 179 177	111 179 221	45	26.753	16.755	26.559	8.16e-28	98.1
ENSMUSG00000001911	Nfix	nuclear factor I/X [Source:MGI Symbol;Acc:MGI:97311]	8	E018	1.461	0.246	se	protein_coding	chr8	-	84 723 728	84 723 850	123	32.819	48.73	35.235	5.26e-62	84.8
ENSMUSG00000038886	Man2a2	mannosidase 2, alpha 2 [Source:MGI Symbol;Acc:MGI:2150656]		E028	1.457	0.826	afe	protein_coding	chr7	-	80 363 463	80 363 466	4	5.945	9.797	7.895	2.62e-03	49.4
ENSMUSG00000028207	Asph	aspartate-beta-hydroxylase [Source:MGI Symbol;Acc:MGI:1914186]	18	E017	1.453	-0.109	mult	protein_coding	chr4	-	9 594 663	9 594 698	36	11.5	18.663	11.082	6.17e-09	105.8
ENSMUSG00000023092	Fhl1	four and a half LIM domains 1 [Source:MGI Symbol;Acc:MGI:1298387]		E010	-1.448	2.363	afe	protein_coding	chrX	+	56 786 527	56 786 582	56	17.166	10.567	35.713	8.07e-02	381
ENSMUSG00000027257	Pacsin3	protein kinase C and casein kinase substrate in neurons 3 [Source:MGI Symbol;Acc:MGI:1891410]	2	E009	-1.442	-0.347	afe	protein_coding	chr2	+	91 256 813	91 256 837	25	19.277	11.939	17.208	3.36e-14	71.8
ENSMUSG00000045962	Wnk1	WNK lysine deficient protein kinase 1 [Source:MGI Symbol;Acc:MGI:2442092]	14	E057	1.433	0.71	afe	protein_coding	chr6	-	119 956 171	119 956 196	26	12.331	19.828	15.639	6.49e-02	55.9
ENSMUSG00000026031	Cflar	CASP8 and FADD-like apoptosis regulator [Source:MGI Symbol;Acc:MGI:1336166]	1	E003	1.432	1.121	afe	protein_coding	chr1	+	58 711 516	58 711 533	18	5.528	9.039	8.125	3.66e-03	26
ENSMUSG00000028478	Clta	clathrin, light polypeptide (Lca) [Source:MGI Symbol;Acc:MGI:894297]	6	E011	1.432	-0.231	afe	protein_coding	chr4	+	44 030 215	44 030 268	54	9.484	15.376	8.766	5.47e-07	112.2
ENSMUSG00000048154	Kmt2d	lysine (K)-specific methyltransferase 2D [Source:MGI Symbol;Acc:MGI:2682319]	18	E046	1.422	1.244	mult	protein_coding	chr15	-	98 857 525	98 857 634	110	10.281	16.574	15.625	1.63e-10	15.1
ENSMUSG00000027291	Vps39	VPS39 HOPS complex subunit [Source:MGI Symbol;Acc:MGI:2443189]	4	E040	-1.421	0.003	se	protein_coding	chr2	-	120 346 857	120 346 889	33	14.086	8.703	14.1	1.87e-07	100.3
ENSMUSG00000034525	Ice1	interactor of little elongation complex ELL subunit 1 [Source:MGI Symbol;Acc:MGI:2385865]	1	E011	1.413	0.711	afe	protein_coding	chr13	-	70 615 074	70 615 113	40	6.467	10.487	8.255	3.19e-04	55.5
ENSMUSG00000048154	Kmt2d	lysine (K)-specific methyltransferase 2D [Source:MGI Symbol;Acc:MGI:2682319]	15	E049	1.409	1.059	mult	protein_coding	chr15	-	98 859 510	98 859 614	105	10.81	17.327	15.434	8.53e-12	29
ENSMUSG00000045962	Wnk1	WNK lysine deficient protein kinase 1 [Source:MGI Symbol;Acc:MGI:2442092]	14	E055	1.405	0.663	afe	protein_coding	chr6	-	119 956 071	119 956 095	25	12.711	20.233	15.864	7.91e-02	58.1
ENSMUSG00000020744	Slc25a19	solute carrier family 25 (mitochondrial thiamine pyrophosphate carrier), member 19 [Source:MGI Symbol;Acc:MGI:1914533]		E013	1.402	0.496	afe	protein_coding	chr11	-	115 623 412	115 623 417	6	6.453	10.428	7.653	3.00e-02	69.8
ENSMUSG00000028207	Asph	aspartate-beta-hydroxylase [Source:MGI Symbol;Acc:MGI:1914186]	17	E018	1.397	-0.058	mult	protein_coding	chr4	-	9 595 377	9 595 415	39	11.45	18.25	11.226	5.35e-12	103.3
ENSMUSG00000048154	Kmt2d	lysine (K)-specific methyltransferase 2D [Source:MGI Symbol;Acc:MGI:2682319]	16	E048	1.394	0.948	mult	protein_coding	chr15	-	98 858 042	98 858 223	182	12.426	19.721	17.05	1.17e-07	36.6
ENSMUSG00000005625	Psmd4	proteasome (prosome, macropain) 26S subunit, non-ATPase, 4 [Source:MGI Symbol;Acc:MGI:1201670]	10	E001	-1.393	-0.436	ale	protein_coding	chr3	-	95 032 694	95 032 696	3	17.512	10.988	15.161	6.15e-08	64
ENSMUSG00000029472	Anapc5	anaphase-promoting complex subunit 5 [Source:MGI Symbol;Acc:MGI:1929722]		E044	1.393	-0.598	afe	protein_coding	chr5	-	122 818 736	122 818 853	118	14.333	22.598	11.723	6.60e-31	131.6
ENSMUSG00000031700	Gpt2	glutamic pyruvate transaminase (alanine aminotransferase) 2 [Source:MGI Symbol;Acc:MGI:1915391]		E011	-1.393	-0.174	afe	protein_coding	chr8	+	85 515 977	85 516 157	181	32.74	21.335	31.105	3.96e-08	85.7
ENSMUSG00000020315	Sptbn1	spectrin beta, non-erythrocytic 1 [Source:MGI Symbol;Acc:MGI:98388]	10	E037	1.39	0.657	se	protein_coding	chr11	-	30 146 739	30 146 851	113	11.759	18.679	14.666	6.76e-02	58
ENSMUSG00000025401	Myo1a	myosin IA [Source:MGI Symbol;Acc:MGI:107732]	26	E026	1.385	1.623	se	protein_coding	chr10	+	127 720 153	127 720 280	128	8.136	13.026	14.109	7.14e-02	-22.1
ENSMUSG00000073557	Ppp1r12b	protein phosphatase 1, regulatory (inhibitor) subunit 12B [Source:MGI Symbol;Acc:MGI:1916417]	30	E008	-1.385	-0.073	afe	protein_coding	chr1	-	134 765 897	134 765 942	46	35.007	23.026	34.281	8.14e-13	93.9
ENSMUSG00000001419	Mef2d	myocyte enhancer factor 2D [Source:MGI Symbol;Acc:MGI:99533]	4	E008	1.383	-0.162	mxe	protein_coding	chr3	+	88 158 034	88 158 171	138	29.299	43.322	27.892	1.24e-31	110
ENSMUSG00000057229	Atp5sl	ATP5S-like [Source:MGI Symbol;Acc:MGI:1913599]		E011	-1.377	-0.197	ale	protein_coding	chr7	+	25 621 973	25 622 003	31	15.08	9.473	14.118	2.17e-11	82.9
ENSMUSG00000028465	Tln1	talin 1 [Source:MGI Symbol;Acc:MGI:1099832]	1	E073	1.376	0.338	afe	protein_coding	chr4	-	43 562 130	43 562 157	28	16.653	25.901	18.608	7.37e-41	78.9
ENSMUSG00000045962	Wnk1	WNK lysine deficient protein kinase 1 [Source:MGI Symbol;Acc:MGI:2442092]	14	E056	1.376	0.677	afe	protein_coding	chr6	-	119 956 096	119 956 170	75	14.032	22.032	17.565	5.00e-02	55.8
ENSMUSG00000055320	Tead1	TEA domain family member 1 [Source:MGI Symbol;Acc:MGI:101876]	13	E036	-1.373	-0.289	ale	protein_coding	chr7	+	112 899 991	112 906 805	6815	109.085	89.086	104.851	3.10e-02	78.8
ENSMUSG00000048154	Kmt2d	lysine (K)-specific methyltransferase 2D [Source:MGI Symbol;Acc:MGI:2682319]	53	E005	-1.372	-1.233	mult	protein_coding	chr15	-	98 834 908	98 834 981	74	39.614	26.548	27.702	2.70e-24	8.8
ENSMUSG00000028207	Asph	aspartate-beta-hydroxylase [Source:MGI Symbol;Acc:MGI:1914186]	16	E020	1.366	-0.135	mult	protein_coding	chr4	-	9 598 269	9 598 301	33	11.446	18.059	10.934	1.37e-10	107.7
ENSMUSG00000024845	Tmem134	transmembrane protein 134 [Source:MGI Symbol;Acc:MGI:1914240]		E016	1.363	0.168	ir	processed_transcript	chr19	+	4 127 640	4 127 697	58	14.103	22.048	14.917	1.08e-11	89.8
ENSMUSG00000024845	Tmem134	transmembrane protein 134 [Source:MGI Symbol;Acc:MGI:1914240]		E018	1.362	0.133	ir	processed_transcript	chr19	+	4 127 740	4 127 759	20	13.531	21.183	14.148	4.51e-12	91.9
ENSMUSG00000035247	Hectd1	HECT domain containing 1 [Source:MGI Symbol;Acc:MGI:2384768]	15	E033	1.362	0.773	se	protein_coding	chr12	-	51 790 172	51 790 248	77	12.552	19.711	16.254	3.75e-02	48.3
ENSMUSG00000028207	Asph	aspartate-beta-hydroxylase [Source:MGI Symbol;Acc:MGI:1914186]	12	E024	1.354	-0.064	mult	protein_coding	chr4	-	9 604 516	9 604 629	114	14.103	21.983	13.804	1.04e-03	103.8
ENSMUSG00000051910	Sox6	SRY (sex determining region Y)-box 6 [Source:MGI Symbol;Acc:MGI:98368]		E001	-1.352	-0.21	ale	protein_coding	chr7	-	115 470 872	115 471 343	472	57.892	41.504	55.195	9.44e-02	83.5
ENSMUSG00000001017	Chtop	chromatin target of PRMT1 [Source:MGI Symbol;Acc:MGI:1913761]		E011	-1.35	-0.098	se	protein_coding	chr3	-	90 505 395	90 505 478	84	20.819	13.343	20.171	9.17e-09	91.3
ENSMUSG00000027559	Car3	carbonic anhydrase 3 [Source:MGI Symbol;Acc:MGI:88270]		E007	-1.349	-0.929	afe	protein_coding	chr3	+	14 869 178	14 869 234	57	22.145	14.237	16.369	4.48e-04	27
ENSMUSG00000034006	Pqlc1	PQ loop repeat containing 1 [Source:MGI Symbol;Acc:MGI:1914193]	5	E007	1.349	1.316	afe	protein_coding	chr18	+	80 256 284	80 256 287	4	4.264	6.788	6.711	6.11e-02	3
ENSMUSG00000048154	Kmt2d	lysine (K)-specific methyltransferase 2D [Source:MGI Symbol;Acc:MGI:2682319]	54	E004	-1.347	-1.199	mult	protein_coding	chr15	-	98 834 716	98 834 824	109	42.975	29.344	30.672	2.20e-29	9.7
ENSMUSG00000036918	Ttc7	tetratricopeptide repeat domain 7 [Source:MGI Symbol;Acc:MGI:1920999]	11	E020	-1.346	-0.553	ale	protein_coding	chr17	+	87 334 267	87 334 273	7	10.635	6.712	8.81	4.76e-07	53.5
ENSMUSG00000061723	Tnnt3	troponin T3, skeletal, fast [Source:MGI Symbol;Acc:MGI:109550]	5	E011	1.344	0.632	afe	protein_coding	chr7	+	142 504 023	142 504 047	25	45.833	62.781	53.525	3.54e-20	54.6
ENSMUSG00000028207	Asph	aspartate-beta-hydroxylase [Source:MGI Symbol;Acc:MGI:1914186]	29	E005	1.343	-0.707	afe	protein_coding	chr4	-	9 475 459	9 475 481	23	9.755	15.341	7.661	1.03e-07	137.5

ENSMUSG00000048154	Kmt2d	lysine (K)-specific methyltransferase 2D [Source:MGI Symbol;Acc:MGI:2682319]	55	E003	-1.34	-1.174	mult	protein_coding	chr15	-	98 834 301	98 834 592	292	49.982	35.002	36.689	9.45e-28	11.3
ENSMUSG00000024845	Tmem134	transmembrane protein 134 [Source:MGI Symbol;Acc:MGI:1914240]		E017	1.337	0.153	ir	processed_transcript	chr19	+	4 127 698	4 127 739	42	14.439	22.361	15.194	1.07e-12	90.5
ENSMUSG00000023830	Igf2r	insulin-like growth factor 2 receptor [Source:MGI Symbol;Acc:MGI:96435]	5	E050	1.331	0.53	ale	protein_coding	chr17	-	12 748 644	12 748 652	9	7.066	11.133	8.475	6.81e-02	65.4
ENSMUSG00000048154	Kmt2d	lysine (K)-specific methyltransferase 2D [Source:MGI Symbol;Acc:MGI:2682319]	52	E006	-1.327	-1.191	mult	protein_coding	chr15	-	98 835 182	98 835 467	286	48.009	33.525	34.868	1.14e-16	9.3
ENSMUSG00000085779	Atcayos	ataxia, cerebellar, Cayman type, opposite strand [Source:MGI Symbol;Acc:MGI:1916928]		E004	-1.321	-0.091	ale	antisense	chr10	+	81 197 204	81 198 439	1236	58.316	42.212	57.142	5.47e-16	92.7
ENSMUSG00000025162	Csnk1d	casein kinase 1, delta [Source:MGI Symbol;Acc:MGI:1355272]	11	E004	-1.318	-0.106	se	protein_coding	chr11	-	120 964 948	120 965 010	63	26.303	17.251	25.457	1.85e-34	90.7
ENSMUSG00000028207	Asph	aspartate-beta-hydroxylase [Source:MGI Symbol;Acc:MGI:1914186]	29	E004	1.317	-0.407	afe	protein_coding	chr4	-	9 475 390	9 475 458	69	10.491	16.328	9.133	3.45e-04	123.3
ENSMUSG00000008822	Acyp1	acylphosphatase 1, erythrocyte (common) type [Source:MGI Symbol;Acc:MGI:1913454]		E009	-1.316	0.177	afe	protein_coding	chr12	-	85 288 178	85 288 438	261	24.769	16.196	26.167	1.10e-18	116.3
ENSMUSG00000041329	Atp1b2	ATPase, Na+/K+ transporting, beta 2 polypeptide [Source:MGI Symbol;Acc:MGI:88109]		E019	1.314	0.408	afe	protein_coding	chr11	-	69 603 554	69 603 613	60	14.036	21.603	16.079	4.11e-10	73
ENSMUSG00000036918	Ttc7	tetratricopeptide repeat domain 7 [Source:MGI Symbol;Acc:MGI:1920999]	17	E028	1.313	0.502	ale	protein_coding	chr17	+	87 361 849	87 361 921	73	16.554	25.261	19.509	9.09e-22	66.1
ENSMUSG00000048154	Kmt2d	lysine (K)-specific methyltransferase 2D [Source:MGI Symbol;Acc:MGI:2682319]	17	E047	1.303	0.984	mult	protein_coding	chr15	-	98 857 722	98 857 886	165	11.874	18.33	16.505	3.27e-10	28.3
ENSMUSG00000060913	Trim55	tripartite motif-containing 55 [Source:MGI Symbol;Acc:MGI:3036269]	9	E011	-1.297	-0.019	se	protein_coding	chr3	+	19 674 254	19 674 541	288	43.529	30.22	43.311	7.03e-26	98.4
ENSMUSG00000022744	Cldnd1	claudin domain containing 1 [Source:MGI Symbol;Acc:MGI:2447860]		E006	-1.296	-0.044	se	protein_coding	chr16	+	58 729 158	58 729 172	15	8.588	5.503	8.46	7.82e-03	95.9
ENSMUSG00000073557	Ppp1r12b	protein phosphatase 1, regulatory (inhibitor) subunit 12B [Source:MGI Symbol;Acc:MGI:1916417]	30	E009	-1.294	-0.066	afe	protein_coding	chr1	-	134 765 943	134 766 077	135	38.499	26.337	37.805	2.09e-10	94.3
ENSMUSG00000048154	Kmt2d	lysine (K)-specific methyltransferase 2D [Source:MGI Symbol;Acc:MGI:2682319]	51	E008	-1.293	-1.097	afe	protein_coding	chr15	-	98 837 075	98 837 121	47	37.739	25.763	27.366	1.93e-14	13.4
ENSMUSG00000036918	Ttc7	tetratricopeptide repeat domain 7 [Source:MGI Symbol;Acc:MGI:1920999]	17	E029	1.291	0.613	ale	protein_coding	chr17	+	87 361 922	87 361 943	22	14.744	22.476	18.057	7.22e-15	57.2
ENSMUSG00000052934	Fbxo31	F-box protein 31 [Source:MGI Symbol;Acc:MGI:1354708]		E015	-1.291	-0.009	se	protein_coding	chr8	-	121 564 638	121 564 724	87	41.828	28.936	41.728	1.98e-11	99.2
ENSMUSG00000028207	Asph	aspartate-beta-hydroxylase [Source:MGI Symbol;Acc:MGI:1914186]	19	E016	1.288	-0.023	mult	protein_coding	chr4	-	9 583 812	9 583 856	45	11.921	18.307	11.827	5.32e-04	101.5
ENSMUSG00000053929	Cyhr1	cysteine and histidine rich 1 [Source:MGI Symbol;Acc:MGI:1859320]	2	E009	1.285	0.039	ale	protein_coding	chr15	-	76 659 440	76 659 505	66	10.738	16.526	10.879	7.46e-05	97.5
ENSMUSG00000026773	Pfkfb3	6-phosphofructo-2-kinase/fructose-2,6-biphosphatase 3 [Source:MGI Symbol;Acc:MGI:2181202]	19	E002	-1.283	0.136	ale	protein_coding	chr2	-	11 471 791	11 471 823	33	20.77	13.614	21.694	1.03e-04	112.9
ENSMUSG00000023118	Sympk	symplekin [Source:MGI Symbol;Acc:MGI:1915438]		E005	-1.282	-0.539	se	protein_coding	chr7	+	19 027 897	19 028 022	126	19.946	13.055	16.726	1.82e-03	53.3
ENSMUSG00000029446	Psph	phosphoserine phosphatase [Source:MGI Symbol;Acc:MGI:97788]		E011	-1.269	-0.135	se	protein_coding	chr5	-	129 776 830	129 776 865	36	15.051	9.811	14.391	1.60e-09	87.4
ENSMUSG00000038708	Golga4	golgi autoantigen, golgin subfamily a, 4 [Source:MGI Symbol;Acc:MGI:1859646]	25	E048	1.266	0.164	ale	protein_coding	chr9	+	118 579 542	118 579 592	51	9.68	14.842	10.234	3.18e-05	89.3
ENSMUSG00000038708	Golga4	golgi autoantigen, golgin subfamily a, 4 [Source:MGI Symbol;Acc:MGI:1859646]	25	E049	1.264	0.023	ale	protein_coding	chr9	+	118 579 593	118 579 606	14	8.211	12.619	8.275	1.83e-02	98.5
ENSMUSG00000019194	Scn1b	sodium channel, voltage-gated, type I, beta [Source:MGI Symbol;Acc:MGI:98247]		E002	1.259	0.333	ir	protein_coding	chr7	-	31 117 137	31 117 223	87	34.869	48.826	38.293	1.90e-38	75.5
ENSMUSG00000026150	Mff	mitochondrial fission factor [Source:MGI Symbol;Acc:MGI:1922984]		E009	-1.242	-0.129	se	protein_coding	chr1	+	82 728 067	82 728 238	172	28.79	19.483	27.683	1.60e-09	88.1
ENSMUSG00000048154	Kmt2d	lysine (K)-specific methyltransferase 2D [Source:MGI Symbol;Acc:MGI:2682319]	51	E009	-1.237	-1.111	afe	protein_coding	chr15	-	98 837 122	98 837 205	84	38.967	27.171	28.236	2.91e-11	9
ENSMUSG00000036550	Cnot1	CCR4-NOT transcription complex, subunit 1 [Source:MGI Symbol;Acc:MGI:2442402]	1	E084	1.234	0.008	afe	protein_coding	chr8	-	95 807 382	95 807 396	15	6.552	9.996	6.57	9.42e-02	99.5
ENSMUSG00000031812	Map1lc3b	microtubule-associated protein 1 light chain 3 beta [Source:MGI Symbol;Acc:MGI:1914693]		E009	-1.228	-0.398	ir	protein_coding	chr8	+	121 593 546	121 593 650	105	18.933	12.595	16.615	3.01e-05	63.4
ENSMUSG00000061462	Obscn	obscurin, cytoskeletal calmodulin and titin-interacting RhoGEF [Source:MGI Symbol;Acc:MGI:2681862]	114	E002	-1.228	0.136	mult	protein_coding	chr11	-	58 994 259	58 994 509	251	70.588	54.273	72.477	4.13e-08	111.6
ENSMUSG00000051910	Sox6	SRY (sex determining region Y)-box 6 [Source:MGI Symbol;Acc:MGI:98368]	22	E002	-1.227	-0.451	ale	protein_coding	chr7	-	115 471 344	115 474 455	3112	85.31	68.093	78.893	3.32e-06	62.7
ENSMUSG00000025586	Cpeb1	cytoplasmic polyadenylation element binding protein 1 [Source:MGI Symbol;Acc:MGI:108442]	7	E013	1.226	0.283	se	protein_coding	chr7	-	81 356 894	81 356 908	15	5.557	8.466	6.125	4.71e-02	80.5
ENSMUSG00000029152	Ociad1	OCIA domain containing 1 [Source:MGI Symbol;Acc:MGI:1915345]	7	E022	-1.223	-0.116	afe	protein_coding	chr5	+	73 306 781	73 306 798	18	27.831	18.907	26.862	7.50e-35	89.1
ENSMUSG00000079055	Slc8a3	solute carrier family 8 (sodium/calcium exchanger), member 3 [Source:MGI Symbol;Acc:MGI:107976]	4	E010	1.218	-0.252	mxe	protein_coding	chr12	-	81 214 066	81 214 169	104	18.762	27.59	17.273	5.61e-14	116.9
ENSMUSG00000018661	Cog1	component of oligomeric golgi complex 1 [Source:MGI Symbol;Acc:MGI:1333873]		E025	-1.216	0.096	se	protein_coding	chr11	+	113 661 659	113 661 665	7	21.523	14.446	22.196	4.17e-10	109.5
ENSMUSG00000026150	Mff	mitochondrial fission factor [Source:MGI Symbol;Acc:MGI:1922984]	1	E003	-1.215	0.045	afe	protein_coding	chr1	+	82 724 919	82 724 924	6	7.617	5.015	7.736	7.43e-02	104.6
ENSMUSG00000048154	Kmt2d	lysine (K)-specific methyltransferase 2D [Source:MGI Symbol;Acc:MGI:2682319]	34	E026	1.212	1.089	mult	protein_coding	chr15	-	98 851 395	98 851 531	137	15.175	22.533	21.667	1.96e-10	11.8
ENSMUSG00000023830	Igf2r	insulin-like growth factor 2 receptor [Source:MGI Symbol;Acc:MGI:96435]	21	E035	1.211	0.656	se	protein_coding	chr17	-	12 715 378	12 715 493	116	11.843	17.737	14.762	4.75e-05	50.5
ENSMUSG00000061462	Obscn	obscurin, cytoskeletal calmodulin and titin-interacting RhoGEF [Source:MGI Symbol;Acc:MGI:2681862]	107	E009	-1.209	-0.087	mult	protein_coding	chr11	-	58 997 163	58 997 285	123	73.72	57.403	72.512	1.25e-14	92.6
ENSMUSG00000061462	Obscn	obscurin, cytoskeletal calmodulin and titin-interacting RhoGEF [Source:MGI Symbol;Acc:MGI:2681862]	106	E010	-1.209	-0.095	mult	protein_coding	chr11	-	58 997 975	58 998 142	168	79.151	62.493	77.811	2.03e-27	92
ENSMUSG00000061462	Obscn	obscurin, cytoskeletal calmodulin and titin-interacting RhoGEF [Source:MGI Symbol;Acc:MGI:2681862]	105	E011	-1.209	-0.088	mult	protein_coding	chr11	-	58 998 261	58 998 353	93	75.623	59.174	74.396	2.81e-31	92.5
ENSMUSG00000058056	Palld	palladin, cytoskeletal associated protein [Source:MGI Symbol;Acc:MGI:1919583]		E003	-1.206	-0.026	afe	protein_coding	chr8	-	61 515 021	61 515 034	14	24.384	16.519	24.186	1.76e-22	97.5
ENSMUSG00000025871	4833439L19Rik	RIKEN cDNA 4833439L19 gene [Source:MGI Symbol;Acc:MGI:1921162]	3	E014	1.203	0.126	se	protein_coding	chr13	-	54 564 185	54 564 272	88	29.215	41.201	30.34	2.50e-71	90.6
ENSMUSG00000000131	Xpo6	exportin 6 [Source:MGI Symbol;Acc:MGI:2429950]		E008	-1.198	-0.007	se	protein_coding	chr7	-	126 103 623	126 103 715	93	19.41	13.055	19.366	2.92e-10	99.3
ENSMUSG00000026131	Dst	dystonin [Source:MGI Symbol;Acc:MGI:104627]	72	E093	1.198	0.63	se	protein_coding	chr1	+	34 249 804	34 250 130	327	22.108	31.952	26.93	6.80e-02	51
ENSMUSG00000026950	Neb	nebulin [Source:MGI Symbol;Acc:MGI:97292]	152	E010	1.195	0.055	mxe	protein_coding	chr2	-	52 150 537	52 150 629	93	92.817	110.255	93.609	7.72e-03	95.5
ENSMUSG00000048154	Kmt2d	lysine (K)-specific methyltransferase 2D [Source:MGI Symbol;Acc:MGI:2682319]	36	E024	1.195	1.332	mult	protein_coding	chr15	-	98 847 358	98 847 481	124	19.822	28.848	30.056	8.46e-13	-13.4
ENSMUSG00000018661	Cog1	component of oligomeric golgi complex 1 [Source:MGI Symbol;Acc:MGI:1333873]		E026	-1.193	0.062	se	protein_coding	chr11	+	113 661 666	113 661 693	28	21.752	14.718	22.184	1.62e-15	106.1

ENSMUSG00000058056	Palld	palladin, cytoskeletal associated protein [Source:MGI Symbol;Acc:MGI:1919583]		E002	-1.185	-0.073	afe	protein_coding	chr8	-	61 514 925	61 515 020	96	25.799	17.657	25.224	3.10e-26	92.9
ENSMUSG00000085614	1700123M08Rik	RIKEN cDNA 1700123M08 gene [Source:MGI Symbol;Acc:MGI:1923915]		E005	-1.183	-0.232	afe	processed_transcript	chr4	+	11 980 142	11 980 161	20	11.117	7.423	10.275	3.18e-05	77.2
ENSMUSG00000061462	Obscn	obscurin, cytoskeletal calmodulin and titin-interacting RhoGEF [Source:MGI Symbol;Acc:MGI:2681862]	93	E023	-1.182	-0.191	mult	protein_coding	chr11	-	59 008 041	59 008 209	169	64.856	49.614	62.296	1.39e-24	83.2
ENSMUSG00000022139	Mbnl2	muscleblind-like 2 [Source:MGI Symbol;Acc:MGI:2145597]	12	E008	1.176	0.339	se	protein_coding	chr14	+	120 404 638	120 404 732	95	16.182	23.68	18.091	1.12e-06	74.5
ENSMUSG00000061462	Obscn	obscurin, cytoskeletal calmodulin and titin-interacting RhoGEF [Source:MGI Symbol;Acc:MGI:2681862]	113	E003	-1.176	0.145	mult	protein_coding	chr11	-	58 994 685	58 994 834	150	78.591	62.399	80.646	1.14e-08	112.7
ENSMUSG00000029152	Ociad1	OCIA domain containing 1 [Source:MGI Symbol;Acc:MGI:1915345]	7	E021	-1.175	-0.126	afe	protein_coding	chr5	+	73 306 754	73 306 780	27	27.369	18.861	26.326	6.58e-31	87.8
ENSMUSG00000061462	Obscn	obscurin, cytoskeletal calmodulin and titin-interacting RhoGEF [Source:MGI Symbol;Acc:MGI:2681862]	104	E012	-1.175	-0.057	mult	protein_coding	chr11	-	58 998 481	58 998 561	81	72.82	57.003	72.025	1.20e-21	95
ENSMUSG00000018501	Ncor1	nuclear receptor co-repressor 1 [Source:MGI Symbol;Acc:MGI:1349717]	25	E047	1.169	-0.292	se, fe	protein_coding	chr11	-	62 359 761	62 359 791	31	6.8	10.144	6.15	8.74e-06	119.4
ENSMUSG00000019795	Pcmt1	protein-L-isoaspartate (D-aspartate) O-methyltransferase 1 [Source:MGI Symbol;Acc:MGI:97502]	8	E001	-1.162	-0.486	ale	protein_coding	chr10	-	7 629 373	7 629 380	8	12.724	8.575	10.796	7.08e-02	53.5
ENSMUSG00000026031	Cflar	CASP8 and FADD-like apoptosis regulator [Source:MGI Symbol;Acc:MGI:1336166]	1	E004	1.159	0.957	afe	protein_coding	chr1	+	58 711 534	58 711 543	10	6.306	9.382	8.757	2.33e-02	20.3
ENSMUSG00000061462	Obscn	obscurin, cytoskeletal calmodulin and titin-interacting RhoGEF [Source:MGI Symbol;Acc:MGI:2681862]	111	E005	-1.156	0.072	mult	protein_coding	chr11	-	58 995 338	58 995 561	224	82.196	66.096	83.223	2.64e-12	106.4
ENSMUSG00000061462	Obscn	obscurin, cytoskeletal calmodulin and titin-interacting RhoGEF [Source:MGI Symbol;Acc:MGI:2681862]	103	E013	-1.155	-0.127	mult	protein_coding	chr11	-	58 999 195	59 001 178	1984	108.772	91.936	106.911	7.02e-30	88.9
ENSMUSG00000026031	Cflar	CASP8 and FADD-like apoptosis regulator [Source:MGI Symbol;Acc:MGI:1336166]	1	E006	1.154	0.736	afe	protein_coding	chr1	+	58 711 551	58 711 559	9	7.441	11.033	9.572	2.01e-02	40.7
ENSMUSG00000061462	Obscn	obscurin, cytoskeletal calmodulin and titin-interacting RhoGEF [Source:MGI Symbol;Acc:MGI:2681862]	102	E014	-1.15	-0.228	mult	protein_coding	chr11	-	59 001 454	59 001 531	78	65.051	50.185	61.993	2.04e-38	79.4
ENSMUSG00000061462	Obscn	obscurin, cytoskeletal calmodulin and titin-interacting RhoGEF [Source:MGI Symbol;Acc:MGI:2681862]	100	E016	-1.148	-0.204	mult	protein_coding	chr11	-	59 002 017	59 002 169	153	68.556	53.418	65.779	2.95e-52	81.7
ENSMUSG00000061462	Obscn	obscurin, cytoskeletal calmodulin and titin-interacting RhoGEF [Source:MGI Symbol;Acc:MGI:2681862]		E025	-1.148	-0.129	mult	protein_coding	chr11	-	59 009 098	59 009 222	125	62.193	47.631	60.486	3.04e-29	88.3
ENSMUSG00000061462	Obscn	obscurin, cytoskeletal calmodulin and titin-interacting RhoGEF [Source:MGI Symbol;Acc:MGI:2681862]	43	E087	1.148	0.64	mult	protein_coding	chr11	-	59 064 187	59 064 450	264	34.388	46.922	41.094	2.38e-03	46.5
ENSMUSG00000005625	Psmd4	proteasome (prosome, macropain) 26S subunit, non-ATPase, 4 [Source:MGI Symbol;Acc:MGI:1201670]		E002	-1.147	-0.334	ale	protein_coding	chr3	-	95 032 697	95 032 703	7	18.343	12.525	16.438	5.07e-07	67.2
ENSMUSG00000029446	Psph	phosphoserine phosphatase [Source:MGI Symbol;Acc:MGI:97788]		E012	-1.145	0.086	se	protein_coding	chr5	-	129 776 866	129 776 941	76	16.35	11.137	16.819	3.91e-11	109
ENSMUSG00000061462	Obscn	obscurin, cytoskeletal calmodulin and titin-interacting RhoGEF [Source:MGI Symbol;Acc:MGI:2681862]	92	E024	-1.145	-0.145	mult	protein_coding	chr11	-	59 008 509	59 008 621	113	63.234	48.599	61.298	2.95e-33	86.8
ENSMUSG00000061462	Obscn	obscurin, cytoskeletal calmodulin and titin-interacting RhoGEF [Source:MGI Symbol;Acc:MGI:2681862]	98	E018	-1.143	-0.182	mult	protein_coding	chr11	-	59 003 207	59 003 379	173	71.551	56.248	69.047	9.15e-46	83.6
ENSMUSG00000061462	Obscn	obscurin, cytoskeletal calmodulin and titin-interacting RhoGEF [Source:MGI Symbol;Acc:MGI:2681862]		E026	-1.14	-0.068	mult	protein_coding	chr11	-	59 009 582	59 009 663	82	56.878	42.998	56.005	3.54e-51	93.7
ENSMUSG00000061462	Obscn	obscurin, cytoskeletal calmodulin and titin-interacting RhoGEF [Source:MGI Symbol;Acc:MGI:2681862]	99	E017	-1.138	-0.16	mult	protein_coding	chr11	-	59 002 991	59 003 017	27	59.603	45.436	57.523	5.54e-58	85.3
ENSMUSG00000048154	Kmt2d	lysine (K)-specific methyltransferase 2D [Source:MGI Symbol;Acc:MGI:2682319]	50	E010	-1.137	-0.986	mult	protein_coding	chr15	-	98 837 295	98 837 431	137	37.8	27.084	28.363	2.71e-12	11.9
ENSMUSG00000061462	Obscn	obscurin, cytoskeletal calmodulin and titin-interacting RhoGEF [Source:MGI Symbol;Acc:MGI:2681862]	112	E004	-1.137	0.118	mult	protein_coding	chr11	-	58 995 028	58 995 103	76	73.939	58.547	75.593	5.26e-10	110.7
ENSMUSG00000061462	Obscn	obscurin, cytoskeletal calmodulin and titin-interacting RhoGEF [Source:MGI Symbol;Acc:MGI:2681862]		E027	-1.135	-0.145	mult	protein_coding	chr11	-	59 010 342	59 010 536	195	63.537	49.002	61.605	5.13e-35	86.7
ENSMUSG00000018411	Mapt	microtubule-associated protein tau [Source:MGI Symbol;Acc:MGI:97180]		E018	-1.134	-0.114	se	protein_coding	chr11	+	104 305 212	104 305 312	101	25.008	17.377	24.134	8.07e-05	88.5
ENSMUSG00000061462	Obscn	obscurin, cytoskeletal calmodulin and titin-interacting RhoGEF [Source:MGI Symbol;Acc:MGI:2681862]	110	E006	-1.134	0.022	mult	protein_coding	chr11	-	58 995 788	58 996 027	240	80.21	64.494	80.524	1.60e-13	102
ENSMUSG00000061462	Obscn	obscurin, cytoskeletal calmodulin and titin-interacting RhoGEF [Source:MGI Symbol;Acc:MGI:2681862]	101	E015	-1.134	-0.235	mult	protein_coding	chr11	-	59 001 879	59 001 924	46	60.988	46.714	57.91	5.33e-32	78.4
ENSMUSG00000024431	Nr3c1	nuclear receptor subfamily 3, group C, member 1 [Source:MGI Symbol;Acc:MGI:95824]	3	E017	1.128	1.013	afe	protein_coding	chr18	-	39 487 233	39 487 245	13	13.676	19.846	19.118	7.49e-11	11.8
ENSMUSG00000024381	Bin1	bridging integrator 1 [Source:MGI Symbol;Acc:MGI:108092]	16	E016	1.127	0.297	se	protein_coding	chr18	+	32 431 671	32 431 742	72	59.009	74.295	62.94	4.26e-21	74.3
ENSMUSG00000026603	Smyd2	SET and MYND domain containing 2 [Source:MGI Symbol;Acc:MGI:1915889]	1	E019	-1.127	-0.091	afe	protein_coding	chr1	-	189 922 254	189 922 363	110	18.434	12.675	17.896	3.90e-02	90.7
ENSMUSG00000035247	Hectd1	HECT domain containing 1 [Source:MGI Symbol;Acc:MGI:2384768]	21	E024	1.127	0.496	se	protein_coding	chr12	-	51 774 651	51 774 790	140	17.139	24.632	20.153	3.52e-02	59.8
ENSMUSG00000022744	Cldnd1	claudin domain containing 1 [Source:MGI Symbol;Acc:MGI:2447860]	2	E008	-1.124	0.138	se	protein_coding	chr16	+	58 729 180	58 729 223	44	12.033	8.208	12.608	1.46e-05	115
ENSMUSG00000032238	Rora	RAR-related orphan receptor alpha [Source:MGI Symbol;Acc:MGI:104661]		E008	1.123	-0.646	se	protein_coding	chr9	+	69 196 075	69 196 104	30	9.238	13.512	7.406	7.62e-03	142.9
ENSMUSG00000061462	Obscn	obscurin, cytoskeletal calmodulin and titin-interacting RhoGEF [Source:MGI Symbol;Acc:MGI:2681862]	94	E022	-1.123	-0.201	mult	protein_coding	chr11	-	59 007 823	59 007 901	79	61.673	47.467	59.019	1.17e-23	81.3
ENSMUSG00000001847	Rac1	RAS-related C3 botulinum substrate 1 [Source:MGI Symbol;Acc:MGI:97845]	1	E012	-1.122	-0.134	afe	protein_coding	chr5	-	143 527 994	143 528 036	43	16.949	11.648	16.212	4.44e-02	86.1
ENSMUSG00000000296	Tpd52l1	tumor protein D52-like 1 [Source:MGI Symbol;Acc:MGI:1298386]	5	E007	1.121	-0.677	se	protein_coding	chr10	-	31 343 012	31 343 026	15	6.942	10.185	5.499	8.29e-02	144.5
ENSMUSG00000013076	Amotl1	angiomotin-like 1 [Source:MGI Symbol;Acc:MGI:1922973]	2	E021	1.12	0.247	se	protein_coding	chr9	-	14 633 902	14 633 952	51	20.654	29.309	22.356	1.74e-14	80.3
ENSMUSG00000018501	Ncor1	nuclear receptor co-repressor 1 [Source:MGI Symbol;Acc:MGI:1349717]	13	E081	1.117	0.41	ss	protein_coding	chr11	-	62 395 193	62 395 228	36	12.232	17.748	14.039	1.02e-09	67.2
ENSMUSG00000008348	Ubc	ubiquitin C [Source:MGI Symbol;Acc:MGI:98889]		E004	-1.115	-0.869	afe	protein_coding	chr5	-	125 389 100	125 389 186	87	17.968	12.395	13.467	2.50e-02	19.2
ENSMUSG00000032449	Slc25a36	solute carrier family 25, member 36 [Source:MGI Symbol;Acc:MGI:1924909]	1	E024	-1.112	0.061	afe	protein_coding	chr9	-	97 110 988	97 111 007	20	9.292	6.346	9.488	1.21e-02	106.7
ENSMUSG00000020882	Cacnb1	calcium channel, voltage-dependent, beta 1 subunit [Source:MGI Symbol;Acc:MGI:102522]	9	E015	1.109	0.176	se	protein_coding	chr11	-	98 010 627	98 010 646	20	13.585	19.598	14.41	2.77e-05	86.3
ENSMUSG00000061462	Obscn	obscurin, cytoskeletal calmodulin and titin-interacting RhoGEF [Source:MGI Symbol;Acc:MGI:2681862]	97	E019	-1.108	-0.197	mult	protein_coding	chr11	-	59 003 629	59 003 706	78	65.301	50.928	62.655	1.56e-53	81.6
ENSMUSG00000006005	Tpr	translocated promoter region, nuclear basket protein [Source:MGI Symbol;Acc:MGI:1922066]	14	E018	1.106	0.152	se	protein_coding	chr1	+	150 411 997	150 412 008	12	8.825	12.847	9.296	7.20e-04	88.3
ENSMUSG00000061950	Ppp4r1	protein phosphatase 4, regulatory subunit 1 [Source:MGI Symbol;Acc:MGI:1917601]		E024	-1.106	-0.361	afe	protein_coding	chr17	+	65 817 000	65 817 027	28	11.386	7.81	10.072	1.70e-03	63.2

ENSMUSG00000061462	Obscn	obscurin, cytoskeletal calmodulin and titin-interacting RhoGEF [Source:MGI Symbol;Acc:MGI:2681862]	108	E008	-1.104	-0.015	mult	protein_coding	chr11	-	58 996 846	58 997 016	171	77.165	62.014	76.953	4.20e-13	98.6
ENSMUSG00000026031	Cflar	CASP8 and FADD-like apoptosis regulator [Source:MGI Symbol;Acc:MGI:1336166]	1	E005	1.102	0.76	afe	protein_coding	chr1	+	58 711 544	58 711 550	7	7.174	10.455	9.306	5.62e-02	35
ENSMUSG00000045962	Wnk1	WNK lysine deficient protein kinase 1 [Source:MGI Symbol;Acc:MGI:2442092]	19	E043	1.099	0.55	afe	protein_coding	chr6	-	119 951 015	119 951 131	117	17.505	24.911	20.935	3.54e-02	53.7
ENSMUSG00000048154	Kmt2d	lysine (K)-specific methyltransferase 2D [Source:MGI Symbol;Acc:MGI:2682319]	35	E025	1.099	1.229	mult	protein_coding	chr15	-	98 849 220	98 851 201	1982	40.703	53.723	55.372	6.71e-17	-12.7
ENSMUSG00000032449	Slc25a36	solute carrier family 25, member 36 [Source:MGI Symbol;Acc:MGI:1924909]	1	E025	-1.096	0.028	afe	protein_coding	chr9	-	97 111 008	97 111 033	26	9.099	6.248	9.185	1.11e-02	103
ENSMUSG00000061462	Obscn	obscurin, cytoskeletal calmodulin and titin-interacting RhoGEF [Source:MGI Symbol;Acc:MGI:2681862]	109	E007	-1.094	0.001	mult	protein_coding	chr11	-	58 996 227	58 996 311	85	70.632	56.013	70.65	8.49e-14	100.1
ENSMUSG00000061462	Obscn	obscurin, cytoskeletal calmodulin and titin-interacting RhoGEF [Source:MGI Symbol;Acc:MGI:2681862]	96	E020	-1.087	-0.194	mult	protein_coding	chr11	-	59 006 757	59 006 817	61	61.499	47.733	58.948	2.68e-40	81.5
ENSMUSG00000047139	Cd24a	CD24a antigen [Source:MGI Symbol;Acc:MGI:88323]	1	E002	-1.086	-0.345	afe	protein_coding	chr10	+	43 579 146	43 579 168	23	20.611	14.423	18.429	2.75e-13	64.7
ENSMUSG00000026202	Tuba4a	tubulin, alpha 4A [Source:MGI Symbol;Acc:MGI:1095410]	5	E002	1.081	0.25	ale	protein_coding	chr1	-	75 214 972	75 215 611	640	72.371	87.665	75.866	4.25e-49	77.2
ENSMUSG00000036918	Ttc7	tetratricopeptide repeat domain 7 [Source:MGI Symbol;Acc:MGI:1920999]	19	E032	1.081	0.297	ale	protein_coding	chr17	+	87 370 459	87 370 661	203	19.278	27.146	21.221	7.44e-13	75.3
ENSMUSG00000000131	Xpo6	exportin 6 [Source:MGI Symbol;Acc:MGI:2429950]		E007	-1.077	-0.103	se	protein_coding	chr7	-	126 103 079	126 103 190	112	22.351	15.739	21.629	3.87e-11	89.1
ENSMUSG00000036918	Ttc7	tetratricopeptide repeat domain 7 [Source:MGI Symbol;Acc:MGI:1920999]	18	E030	1.077	0.347	se	protein_coding	chr17	+	87 363 456	87 363 590	135	18.624	26.244	20.838	3.12e-07	70.9
ENSMUSG00000042675	Ypel3	yippee-like 3 (Drosophila) [Source:MGI Symbol;Acc:MGI:1913340]	1	E005	-1.077	0.182	afe	protein_coding	chr7	+	126 777 001	126 777 004	4	17.36	12.118	18.427	1.22e-02	120.3
ENSMUSG00000004788	Eif2b2	eukaryotic translation initiation factor 2B, subunit 2 beta [Source:MGI Symbol;Acc:MGI:2145118]	1	E001	1.076	0.176	afe	protein_coding	chr12	+	85 219 481	85 219 503	23	6.159	8.91	6.543	4.06e-02	86
ENSMUSG00000018501	Ncor1	nuclear receptor co-repressor 1 [Source:MGI Symbol;Acc:MGI:1349717]	13	E080	1.076	0.402	ss,afe	protein_coding	chr11	-	62 395 186	62 395 192	7	11.693	16.758	13.389	1.37e-07	66.5
ENSMUSG00000061462	Obscn	obscurin, cytoskeletal calmodulin and titin-interacting RhoGEF [Source:MGI Symbol;Acc:MGI:2681862]	95	E021	-1.075	-0.207	mult	protein_coding	chr11	-	59 007 524	59 007 724	201	68.357	54.152	65.536	1.13e-24	80.1
ENSMUSG00000026131	Dst	dystonin [Source:MGI Symbol;Acc:MGI:104627]	73	E094	1.073	0.603	se	protein_coding	chr1	+	34 250 610	34 250 694	85	17.495	24.699	21.279	8.52e-02	47.5
ENSMUSG00000026207	Speg	SPEG complex locus [Source:MGI Symbol;Acc:MGI:109282]	14	E035	1.072	0.393	ale	protein_coding	chr1	+	75 404 208	75 404 370	163	18.112	25.513	20.58	1.32e-04	66.7
ENSMUSG00000028760	Eif4g3	eukaryotic translation initiation factor 4 gamma, 3 [Source:MGI Symbol;Acc:MGI:1923935]		E017	-1.067	0.06	se	protein_coding	chr4	+	138 091 365	138 091 380	16	11.961	8.319	12.207	9.55e-04	106.8
ENSMUSG00000034758	Tle6	transducin-like enhancer of split 6 [Source:MGI Symbol;Acc:MGI:2149593]	7	E029	-1.067	-0.388	ss	protein_coding	chr10	-	81 596 252	81 596 255	4	8.813	6.11	7.716	9.33e-02	59.4
ENSMUSG00000041225	Arhgap12	Rho GTPase activating protein 12 [Source:MGI Symbol;Acc:MGI:1922665]	8	E023	-1.065	-0.502	se	protein_coding	chr18	-	6 057 559	6 057 591	33	10.4	7.226	8.765	8.59e-03	48.5
ENSMUSG00000030592	Ryr1	ryanodine receptor 1, skeletal muscle [Source:MGI Symbol;Acc:MGI:99659]	10	E107	1.06	0.468	se	protein_coding	chr7	-	29 110 845	29 111 001	157	46.812	60.121	52.515	8.60e-02	57.2
ENSMUSG00000020859	Spag9	sperm associated antigen 9 [Source:MGI Symbol;Acc:MGI:1918084]	30	E051	-1.057	-0.004	ale	protein_coding	chr11	+	94 113 263	94 113 301	39	30.918	22.319	30.877	1.60e-25	99.5
ENSMUSG00000024212	Mllt1	myeloid/lymphoid or mixed-lineage leukemia; translocated to, 1 [Source:MGI Symbol;Acc:MGI:1927238]	5	E008	-1.054	0.003	se	protein_coding	chr17	-	56 902 563	56 902 688	126	20.116	14.217	20.132	2.94e-09	100.3
ENSMUSG00000029439	Sfswap	splicing factor, suppressor of white-apricot homolog (Drosophila) [Source:MGI Symbol;Acc:MGI:101760]	15	E044	-1.054	0.273	ale	protein_coding	chr5	+	129 559 469	129 559 499	31	12.008	8.391	13.167	1.31e-02	132
ENSMUSG00000030774	Pak1	p21 protein (Cdc42/Rac)-activated kinase 1 [Source:MGI Symbol;Acc:MGI:1339975]	15	E023	-1.053	-0.109	ale	protein_coding	chr7	+	97 911 884	97 912 381	498	43.573	32.519	42.339	5.37e-07	88.8
ENSMUSG00000002343	Armc6	armadillo repeat containing 6 [Source:MGI Symbol;Acc:MGI:1924063]	1	E017	1.052	0.515	afe	protein_coding	chr8	-	70 234 110	70 234 126	17	8.011	11.465	9.553	3.66e-03	55.3
ENSMUSG00000025401	Myo1a	myosin IA [Source:MGI Symbol;Acc:MGI:107732]	18	E018	-1.05	-0.837	se	protein_coding	chr10	+	127 715 250	127 715 343	94	18.117	12.777	13.726	9.28e-02	17.8
ENSMUSG00000037487	Ubr5	ubiquitin protein ligase E3 component n-recognin 5 [Source:MGI Symbol;Acc:MGI:1918040]	26	E034	1.05	0.027	se	protein_coding	chr15	-	38 007 950	38 008 047	98	13.169	18.653	13.289	6.24e-02	97.8
ENSMUSG00000038084	Opa1	OPA1, mitochondrial dynamin like GTPase [Source:MGI Symbol;Acc:MGI:1921393]	5	E009	-1.048	-0.203	se	protein_coding	chr16	+	29 588 823	29 588 876	54	17.725	12.504	16.58	3.92e-04	78.1
ENSMUSG00000026950	Neb	nebulin [Source:MGI Symbol;Acc:MGI:97292]	144	E022	1.046	0.612	mxe	protein_coding	chr2	-	52 161 371	52 161 475	105	96.281	111.582	105.214	3.99e-07	41.6
ENSMUSG00000048154	Kmt2d	lysine (K)-specific methyltransferase 2D [Source:MGI Symbol;Acc:MGI:2682319]	37	E023	1.045	1.114	mult	protein_coding	chr15	-	98 847 138	98 847 222	85	19.14	26.665	27.231	7.12e-10	-7.5
ENSMUSG00000018411	Mapt	microtubule-associated protein tau [Source:MGI Symbol;Acc:MGI:97180]		E019	-1.041	-0.152	se	protein_coding	chr11	+	104 305 313	104 305 409	97	26.106	18.745	24.901	5.10e-06	83.6
ENSMUSG00000079055	Slc8a3	solute carrier family 8 (sodium/calcium exchanger), member 3 [Source:MGI Symbol;Acc:MGI:107976]	5	E008	-1.037	0.017	se	protein_coding	chr12	-	81 209 454	81 209 471	18	26.344	18.945	26.482	9.43e-13	101.9
ENSMUSG00000036918	Ttc7	tetratricopeptide repeat domain 7 [Source:MGI Symbol;Acc:MGI:1920999]	20	E035	1.034	0.244	afe	protein_coding	chr17	+	87 379 849	87 380 086	238	15.486	21.706	16.791	7.15e-10	79
ENSMUSG00000030399	Ckm	creatine kinase, muscle [Source:MGI Symbol;Acc:MGI:88413]	8.9	E012	-1.033	-0.208	ale	protein_coding	chr7	+	19 421 585	19 422 841	1257	97.694	82.801	94.672	1.42e-27	79.7
ENSMUSG00000027257	Pacsin3	protein kinase C and casein kinase substrate in neurons 3 [Source:MGI Symbol;Acc:MGI:1891410]	1	E006	1.032	0.116	afe	protein_coding	chr2	+	91 256 302	91 256 343	42	29.863	40.124	30.915	9.24e-30	89.7
ENSMUSG00000027288	Zfp106	zinc finger protein 106 [Source:MGI Symbol;Acc:MGI:1270153]	5	E034	1.032	0.487	ale	protein_coding	chr2	-	120 535 287	120 535 419	133	37.774	49.518	43.1	2.75e-04	54.6
ENSMUSG00000052934	Fbxo31	F-box protein 31 [Source:MGI Symbol;Acc:MGI:1354708]		E009	1.029	-0.72	afe	protein_coding	chr8	-	121 559 095	121 559 099	5	6.412	9.125	5.004	4.49e-02	151.9
ENSMUSG00000024431	Nr3c1	nuclear receptor subfamily 3, group C, member 1 [Source:MGI Symbol;Acc:MGI:95824]	10	E003	-1.028	-0.707	ale	protein_coding	chr18	-	39 411 987	39 414 481	2495	76.462	62.354	66.682	4.12e-04	30.7
ENSMUSG00000031309	Rps6ka3	ribosomal protein S6 kinase polypeptide 3 [Source:MGI Symbol;Acc:MGI:104557]		E042	-1.027	-0.08	ale	protein_coding	chrX	+	159 364 503	159 368 244	3742	76.127	62.061	75.002	1.06e-25	92
ENSMUSG00000022744	Cldnd1	claudin domain containing 1 [Source:MGI Symbol;Acc:MGI:2447860]	2	E007	-1.023	0.264	se	protein_coding	chr16	+	58 729 173	58 729 179	7	9.356	6.588	10.236	7.47e-02	131.8
ENSMUSG00000029162	Khk	ketohexokinase [Source:MGI Symbol;Acc:MGI:1096353]		E006	-1.022	-0.513	se	protein_coding	chr5	+	30 926 688	30 926 822	135	29.137	21.185	24.887	1.15e-21	46.6
ENSMUSG00000036550	Cnot1	CCR4-NOT transcription complex, subunit 1 [Source:MGI Symbol;Acc:MGI:2442402]	5	E078	1.017	0.099	se	protein_coding	chr8	-	95 773 571	95 773 639	69	10.706	15.076	11.07	1.60e-02	91.7
ENSMUSG00000060206	Zfp462	zinc finger protein 462 [Source:MGI Symbol;Acc:MGI:107690]	13	E020	-1.015	-0.35	ale	protein_coding	chr4	+	55 081 331	55 081 358	28	9.67	6.83	8.581	5.88e-02	61.7
ENSMUSG00000018501	Ncor1	nuclear receptor co-repressor 1 [Source:MGI Symbol;Acc:MGI:1349717]	13	E079	1.014	0.469	ss,afe	protein_coding	chr11	-	62 395 138	62 395 185	48	12.791	17.922	14.968	4.24e-08	57.6
ENSMUSG00000031146	Plp2	proteolipid protein 2 [Source:MGI Symbol;Acc:MGI:1298382]	1	E010	-1.013	-0.627	afe	protein_coding	chrX	-	7 671 373	7 671 390	18	9.941	7.031	8.024	5.04e-02	34.1
ENSMUSG00000025812	Pard3	par-3 family cell polarity regulator [Source:MGI Symbol;Acc:MGI:2135608]	20	E051	-1.011	-0.301	ale	protein_coding	chr8	+	127 410 732	127 410 773	42	15.757	11.224	14.255	3.84e-06	66.9
ENSMUSG00000048271	Rbm33	RNA binding motif protein 33 [Source:MGI Symbol;Acc:MGI:1919670]		E010	-1.011	-0.059	ale	protein_coding	chr5	+	28 352 644	28 352 695	52	14.722	10.471	14.437	2.19e-04	93.3
ENSMUSG00000018819	Lsp1	lymphocyte specific 1 [Source:MGI Symbol;Acc:MGI:96832]		E019	-1.01	0.287	afe	protein_coding	chr7	+	142 489 017	142 489 128	112	18.906	13.533	20.745	7.70e-03	134.2
ENSMUSG00000073131	Vma21	VMA21 vacuolar H+-ATPase homolog (S. cerevisiae) [Source:MGI Symbol;Acc:MGI:1914298]	1	E004	-1.009	-0.732	afe	protein_coding	chrX	+	71 816 790	71 816 812	23	10.727	7.599	8.359	1.24e-02	24.3
ENSMUSG00000034751	Mast4	microtubule associated serine/threonine kinase family member 4 [Source:MGI Symbol;Acc:MGI:1918885]	30	E003	-1.004	-0.452	ale	protein_coding	chr13	-	102 734 786	102 734 875	90	22.521	16.252	19.475	3.75e-03	51.4
ENSMUSG00000021798	Ldb3	LIM domain binding 3 [Source:MGI Symbol;Acc:MGI:1344412]	14	E004	1.003	0.06	se	protein_coding	chr14	-	34 542 427	34 542 607	181	67.69	81.694	68.504	7.20e-06	94.2
ENSMUSG00000048154	Kmt2d	lysine (K)-specific methyltransferase 2D [Source:MGI Symbol;Acc:MGI:2682319]	49	E011	-1.003	-0.878	mult	protein_coding	chr15	-	98 839 277	98 840 417	1141	58.363	45.905	47.38	5.55e-12	11.8

ENSMUSG00000026207	Speg	SPEG complex locus [Source:MGI Symbol;Acc:MGI:109282]	14	E033	1.002	0.241	ale	protein_coding	chr1	+	75 403 777	75 403 990	214	19.059	26.205	20.605	2.37e-07	78.4
ENSMUSG00000030592	Ryr1	ryanodine receptor 1, skeletal muscle [Source:MGI Symbol;Acc:MGI:99659]	3	E118	1.002	0.428	se	protein_coding	chr7	-	29 117 334	29 117 438	105	44.966	57.312	50.077	9.01e-02	58.6

Supplementary Table 4: Differentially expressed proteins from WT, HSA-LR and Pip6a-PMO treated HSA-LR mice as revealed by label-free mass spectrometry analysis (quadriceps).
(UP: number of unique peptides, US unique sequence coverage %, MW, molecular weight in Kda).

	Protein name	Gene name	Accession #	Fold change	UP	US [%]	MW	p-value
WT versus HSA	Ubiquitin carboxyl-terminal hydrolase isozyme L1	Uchl1	Q9R0P9	29,0	8	56,5	24,8	0,0028
	Musculoskeletal embryonic nuclear protein 1	Mustn1	Q99JI1	20,7	3	48,8	8,9	0,0005
	DnaJ homolog subfamily B member 4	Dnajb4	Q9D832	9,5	7	37,7	37,8	0,0151
	Histone H1.0; Histone H1.0, N-terminally processed	H1f0	P10922	8,1	3	16,5	20,9	0,0045
	Heat shock protein beta-7	Hspb7	P35385	6,9	8	68,0	18,6	0,0025
	Heat shock protein beta-1	Hspb1	P14602	6,3	20	90,9	23,0	0,004
	Heat shock protein beta-8	Hspb8	Q9JK92	6,2	2	18,9	21,5	0,0351
	Nebulin-related-anchoring protein	Nrap	Q80XB4	6,2	10	6,5	195,8	0,01
	Tubulin polymerization-promoting protein family member 3	Tppp3	Q9CRB6	6,2	2	19,3	19,0	0,0288
	Four and a half LIM domains protein 1	Fhl1	P97447	5,3	21	72,5	31,9	0,0117
	LIM and cysteine-rich domains protein 1	Lmcd1	Q8VEE1	5,2	11	38,4	41,0	0,0167
	COP9 signalosome complex subunit 7a	Cops7a	Q9CZ04	5,1	3	14,5	30,2	0,0203
	Xin actin-binding repeat-containing protein 1	Xirp1	O70373	4,8	15	25,5	123,4	0,0123
	Activator of 90 kDa heat shock protein ATPase homolog 1	Ahsa1	Q8BK64	4,7	2	9,8	38,1	0,0049
	60S ribosomal protein L18	Rpl18	P35980	4,5	4	23,9	21,6	0,0063
	Ras-related protein Rab-2A	Rab2a	P53994	4,5	3	21,7	23,5	0,0002
	Heat shock protein beta-6	Hspb6	Q5EBG6	4,1	8	92,0	17,5	0,0326
	60S ribosomal protein L6	Rpl6	P47911	4,0	2	8,1	33,5	0,0125
	Heat shock 70 kDa protein 1A	Hspa1a	Q61696	4,0	5	10,6	70,1	0,026
	Major vault protein	Mvp	Q9EQK5	3,9	3	9,5	95,9	0,0321
	Myosin-1	Myh1	Q5SX40	3,8	69	27,3	223,3	0,0031
	Protein S100-A4	S100a4	P07091	3,7	2	24,8	11,7	0,0009
	Protein diaphanous homolog 1	Diaph1	O08808	3,7	3	2,5	139,3	0,0461
	Adiponectin	Adipoq	Q60994	3,1	2	7,7	26,8	0,0478
	Filamin-C	Flnc	Q8VHX6	2,9	119	62,9	291,1	0,0002
	Biglycan	Bgn	P28653	2,6	5	17,1	41,6	0,0024
	Ankyrin-3	Ank3	G5E8K5	2,4	2	1,2	214,1	0,0221
	Myoglobin	Mb	P04247	2,4	21	87,7	17,1	0,0257
	Endoplasmic reticulum resident protein 44	Erp44	Q9D1Q6	2,3	3	14,3	46,9	0,0173
	Heat shock protein beta-2	Hspb2	Q99PR8	2,3	8	71,4	20,4	0,0002
	PDZ and LIM domain protein 3	Pdlim3	O70209	2,3	11	51,9	34,3	0,0011
	Mitogen-activated protein kinase 1	Mapk1	P63085	2,3	3	15,9	41,3	0,0253
	Alpha-actinin-2	Actn2	Q9JI91	2,2	48	60,1	103,8	0,0059
	ER membrane protein complex subunit 1	Emc1	Q8C7X2	2,2	2	3,4	111,6	0,0072
	Serpin B6	Serpinb6	Q60854	2,2	20	67,2	42,6	0,025
	Calpain-3	Capn3	Q64691	2,1	2	3,9	94,2	0,0034
	Heat shock protein beta-3	Hspb3	Q9QZ57	2,1	2	16,9	17,2	0,0055
	Metaxin-2	Mtx2	O88441	1,9	2	12,5	29,8	0,0153
	Kelch-like protein 40	Klhl40	Q9D783	1,9	17	40,3	69,6	9E-05
	BAG family molecular chaperone regulator 3	Bag3	Q9JLV1	1,8	9	21,8	61,9	0,004
	Calpain-2 catalytic subunit	Capn2	O08529	1,8	7	16,1	79,9	0,0004
	Calcium/calmodulin-dependent protein kinase type II subunit delta	Camk2d	Q6PHZ2	1,7	4	8,8	56,4	0,0238
	Annexin A2	Anxa2	P07356	1,7	11	42,8	38,7	0,0002
	PRA1 family protein 3	Arl6ip5	Q8R5J9	1,7	5	20,2	21,6	0,0041
	Asporin	Aspn	Q99MQ4	1,6	9	31,9	42,6	0,0204
	Protein S100-A6	S100a6	P14069	1,6	3	36,0	10,1	0,0007
	Kelch-like protein 41	Klhl41	A2AUC9	1,6	31	59,7	68,2	0,001
	Vimentin	Vim	P20152	1,6	24	60,1	53,7	0,0042
	Annexin A1	Anxa1	P10107	1,6	7	26,9	38,7	0,0256
	40S ribosomal protein S24	Rps24	P62849	1,6	2	18,0	15,4	0,0382
	Myotilin	Myot	Q9JIF9	1,6	30	77,6	55,3	0,003
	Small muscular protein	Smpx	Q9DC77	1,6	3	64,7	9,3	0,0047
	Annexin A5	Anxa5	P48036	1,5	9	30,4	35,8	0,0067
	Probable C->U-editing enzyme APOBEC-2	Apobec2	Q9WV35	1,5	11	67,0	25,7	0,0066
	Telethonin	Tcap	O70548	1,5	5	37,1	19,1	0,0003
	Phosphorylase b kinase regulatory subunit beta	Phkb	Q7TSH2	-1,5	27	34,7	123,9	0,0222
	Tropomyosin alpha-1 chain	Tpm1	P58771	-1,5	27	54,6	32,7	0,0125
	Phosphoglucomutase-1	Pgm1	Q9D0F9	-1,6	44	89,7	61,4	0,0018
	Glutathione S-transferase Mu 1	Gstm1	P10649	-1,6	14	59,2	26,0	0,0016
	Leucine-rich repeat-containing protein 20	Lrrc20	Q8CI70	-1,6	4	25,0	20,8	0,049
	Secernin-3	Scrn3	Q3TMH2	-1,6	10	37,3	47,7	0,0009
	AMP deaminase 1	Ampd1	Q3V1D3	-1,6	40	60,5	86,1	0,0102
	Glycerol-3-phosphate dehydrogenase, mitochondrial	Gpd2	Q64521	-1,6	29	48,7	81,0	0,0003
	SH3 and cysteine-rich domain-containing protein 3	Stac3	Q8BZ71	-1,6	7	21,1	41,0	0,0154
	Alpha-actinin-3	Actn3	O88990	-1,6	63	67,0	103,0	0,0257
	NADP-dependent malic enzyme	Me1	P06801	-1,9	8	28,8	64,0	0,0166
	Glycerol-3-phosphate dehydrogenase [NAD(+)], cytoplasmic	Gpd1	P13707	-1,9	23	61,9	37,6	0,003
	Myosin-binding protein C, fast-type	Mybpc2	Q5XKE0	-2,2	101	84,1	127,4	0,0068
	Trans-1,2-dihydrobenzene-1,2-diol dehydrogenase	Dhdh	Q9DBB8	-2,7	7	43,2	36,3	0,004
	Glutathione S-transferase Mu 2	Gstm2	P15626	-3,0	9	44,0	25,7	0,0025
	Leukotriene A-4 hydrolase	Lta4h	P24527	-4,3	5	11,5	69,1	0,0407
	Alpha-2-macroglobulin receptor-associated protein	Lrpap1	P55302	-4,6	3	8,3	42,2	0,0001
	Ubiquitin thioesterase OTUB1	Otub1	Q7TQI3	-5,3	3	12,2	31,3	0,0019
	PDZ and LIM domain protein 7	Pdlim7	Q3TJD7	-5,3	9	23,0	50,1	0,0013
	Dermatopontin	Dpt	Q9QZZ6	-5,6	2	16,4	24,0	0,0021
	Ig gamma-1 chain C region, membrane-bound form	Ighg1	P01869	-8,2	2	13,6	35,7	0,0492

Protein name	Gene name	Accession #	Fold change	UP	US [%]	MW	p-value
Ubiquitin carboxyl-terminal hydrolase isozyme L1	Uchl1	Q9R0P9	34,9	8	56,5	24,8	0,0022
Musculoskeletal embryonic nuclear protein 1	Mustn1	Q99JI1	11,0	3	48,8	8,9	0,0005
Fatty acid synthase	Fasn	P19096	10,3	3	3,0	272,4	0,0074
Calnexin	Canx	P35564	6,4	3	12,5	67,3	0,0003
DnaJ homolog subfamily B member 4	Dnajb4	Q9D832	6,1	7	37,7	37,8	0,0310
NADH-ubiquinone oxidoreductase chain 1	Mtnd1	P03888	5,5	2	8,8	36,1	0,0198
Flavin reductase (NADPH)	Blvrb	Q923D2	4,8	5	41,3	22,2	0,0480
Ras-related protein Rab-2A	Rab2a	P53994	4,8	3	21,7	23,5	0,0003
Adiponectin	Adipoq	Q60994	4,7	2	7,7	26,8	0,0031
Succinyl-CoA ligase [GDP-forming] subunit beta, mitochondrial	Suclg2	Q9Z2I8	3,6	2	3,9	46,8	0,0079
6-phosphogluconate dehydrogenase, decarboxylating	Pgd	Q9DCD0	2,9	2	7,7	53,2	0,0027
Cytochrome b5 type B	Cyb5b	Q9CQX2	2,7	2	32,2	16,3	0,0047
Hemoglobin subunit beta-2	Hbb-b2	P02089	2,7	6	46,3	15,9	0,0500
Hemoglobin subunit beta-1	Hbb-b1	P02088	2,5	7	58,5	15,8	0,0307
Plasminogen	Plg	P20918	2,5	3	4,2	90,8	0,0291

WT versus Pip6a-PMO

Heat shock protein beta-1	Hspb1	P14602	2,5	20	90,9	23,0	0,0440
Serpin B6	Serpinb6	Q60854	2,4	20	67,2	42,6	0,0242
Mitogen-activated protein kinase 1	Mapk1	P63085	2,4	3	15,9	41,3	0,0159
Calpain-3	Capn3	Q64691	2,2	2	3,9	94,2	0,0061
Heat shock protein beta-2	Hspb2	Q99PR8	1,9	8	71,4	20,4	0,0029
Protein S100-A4	S100a4	P07091	1,8	2	24,8	11,7	0,0200
Integrin-linked protein kinase	Ilk	O55222	1,8	3	6,9	51,4	0,0017
Annexin A5	Anxa5	P48036	1,7	9	30,4	35,8	0,0025
T-complex protein 1 subunit eta	Cct7	P80313	1,7	6	15,3	59,7	0,0283
Dysferlin	Dysf	Q9ESD7	1,7	32	23,3	237,9	0,0011
Fatty acid-binding protein, adipocyte	Fabp4	P04117	1,7	12	68,9	14,7	0,0481
PDZ and LIM domain protein 3	Pdlim3	O70209	1,7	11	51,9	34,3	0,0150
Alpha-actinin-2	Actn2	Q9JI91	1,6	48	60,1	103,8	0,0399
Filamin-C	Flnc	Q8VHX6	1,6	119	62,9	291,1	0,0054
Calcium/calmodulin-dependent protein kinase type II subunit delta	Camk2d	Q6PHZ2	1,6	4	8,8	56,4	0,0018
Polyubiquitin-B	Ubb	P0CG49	1,6	2	19,5	14,7	0,0004
Annexin A11	Anxa11	P97384	1,6	10	26,4	54,1	0,0028
40S ribosomal protein S24	Rps24	P62849	1,6	2	18,0	15,4	0,0242
Spectrin beta chain, erythrocytic	Sptb	P15508	1,6	19	13,3	245,3	0,0211
Protein-glutamine gamma-glutamyltransferase 2	Tgm2	P21981	1,5	9	21,1	77,1	0,0009
60S acidic ribosomal protein P0	Rplp0	P14869	1,5	4	16,4	34,2	0,0090
40S ribosomal protein S7	Rps7	P62082	1,5	4	35,1	22,1	0,0238
Protein-arginine deiminase type-2	Padi2	Q08642	-1,6	15	32,7	76,2	0,0340
UTP--glucose-1-phosphate uridylyltransferase	Ugp2	Q91ZJ5	-1,7	32	67,1	57,0	0,0385
Metaxin-2	Mtx2	O88441	-1,8	2	12,5	29,8	0,0257
Myelin protein P0	Mpz	P27573	-1,9	7	25,8	27,6	0,0081
Target of Myb protein 1	Tom1	O88746	-2,0	3	11,4	54,3	0,0131
Phospholipid hydroperoxide glutathione peroxidase, mitochondrial	Gpx4	O70325	-2,2	2	13,2	22,2	0,0487
COP9 signalosome complex subunit 8	Cops8	Q8VBV7	-2,5	2	19,1	23,3	0,0372
Glutathione S-transferase Mu 2	Gstm2	P15626	-2,5	9	44,0	25,7	0,0053
40S ribosomal protein S16	Rps16	P14131	-5,5	4	27,4	16,4	0,0015
Prostaglandin reductase 2	Ptgr2	Q8VDQ1	-5,7	2	14,5	38,0	0,0139
Myosin regulatory light chain 12B	Myl12b	Q3THE2	-7,8	3	27,9	19,8	0,0090

HSA versus Pip6a-PMO

Protein name	Gene name	Accession #	Fold change	UP	US [%]	MW	p-value
Ubiquitin thioesterase OTUB1	Otub1	Q7TQI3	7,0	3	12,2	31,3	0,0006
Fatty acid synthase	Fasn	P19096	4,8	3	3,0	272,4	0,0054
Plasminogen	Plg	P20918	4,7	3	4,2	90,8	0,0108
Leukotriene A-4 hydrolase	Lta4h	P24527	4,0	5	11,5	69,1	0,0464
Glycine--tRNA ligase	Gars	Q9CZD3	3,2	4	10,0	81,9	0,0448
PDZ and LIM domain protein 7	Pdlim7	Q3TJD7	3,1	9	23,0	50,1	0,0003
Cytochrome b5	Cyb5a	P56395	2,5	2	22,4	15,2	0,0063
Myosin-binding protein H	Mybph	P70402	1,9	23	67,1	52,6	0,012
Myosin-binding protein C, fast-type	Mybpc2	Q5XKE0	1,8	101	84,1	127,4	0,0108
Hemopexin	Hpx	Q91X72	1,6	13	27,4	51,3	0,0286
Proline-rich protein 33	Prr33	Q8C494	1,6	4	18,5	28,5	0,0132
Carboxylesterase 1D	Ces1d	Q8VCT4	1,5	7	20,0	61,8	0,0115
Glycogen phosphorylase, muscle form	Pygm	Q9WUB3	1,5	67	58,7	97,3	0,0114
40S ribosomal protein S3a	Rps3a	P97351	-1,5	6	25,4	29,9	0,0039
DnaJ homolog subfamily B member 4	Dnajb4	Q9D832	-1,6	7	37,7	37,8	0,0005
Hydroxyacylglutathione hydrolase, mitochondrial	Hagh	Q99KB8	-1,6	4	17,8	34,1	0,0354
UTP--glucose-1-phosphate uridylyltransferase	Ugp2	Q91ZJ5	-1,7	32	67,1	57,0	0,0175
Filamin-C	Flnc	Q8VHX6	-1,8	119	62,9	291,1	0,0007
Heat shock protein beta-3	Hspb3	Q9QZ57	-1,8	2	16,9	17,2	0,0249
Musculoskeletal embryonic nuclear protein 1	Mustn1	Q99JI1	-1,9	3	48,8	8,9	0,0304
Myoglobin	Mb	P04247	-1,9	21	87,7	17,1	0,0058
Four and a half LIM domains protein 1	Fhl1	P97447	-2,0	21	72,5	31,9	0,0051
Protein S100-A4	S100a4	P07091	-2,0	2	24,8	11,7	0,004
Alpha-crystallin B chain	Cryab	P23927	-2,0	13	84,6	20,1	0,0328
Protein-arginine deiminase type-2	Padi2	Q08642	-2,1	15	32,7	76,2	0,0127
Coronin-6	Coro6	Q920M5	-2,3	4	11,3	52,6	0,0493
Microfibrillar-associated protein 5	Mfap5	Q9QZJ6	-2,4	2	12,8	18,5	0,0003
Heat shock protein beta-6	Hspb6	Q5EBG6	-2,4	8	92,0	17,5	0,0017
Heat shock protein beta-1	Hspb1	P14602	-2,5	20	90,9	23,0	0,0012
Endoplasmic reticulum resident protein 44	Erp44	Q9D1Q6	-2,6	3	14,3	46,9	0,0173
60S ribosomal protein L6	Rpl6	P47911	-2,6	2	8,1	33,5	0,0218
Myosin-1	Myh1	Q5SX40	-2,7	69	27,3	223,3	0,0061
Heat shock protein beta-7	Hspb7	P35385	-2,9	8	68,0	18,6	0,0035
Sarcolemmal membrane-associated protein	Slmap	Q3URD3	-3,0	3	4,1	96,9	0,0002
Nebulin-related-anchoring protein	Nrap	Q80XB4	-3,1	10	6,5	195,8	0,0395
Myelin protein P0	Mpz	P27573	-3,1	7	25,8	27,6	0,0069
Metaxin-2	Mtx2	O88441	-3,5	2	12,5	29,8	0,0047
LIM and cysteine-rich domains protein 1	Lmcd1	Q8VEE1	-3,9	11	38,4	41,0	0,0315
40S ribosomal protein S16	Rps16	P14131	-5,6	4	27,4	16,4	0,0019
Histone H1.0;Histone H1.0, N-terminally processed	H1f0	P10922	-6,6	3	16,5	20,9	0,0092
Myosin regulatory light chain 12B	Myl12b	Q3THE2	-8,1	3	27,9	19,8	0,0092

Liver	CTL		2 injections, 2 weeks				3 injections, 2 weeks				3 injections, 4 weeks				3 injections, 6 months			
infiltration mononuclear cells	-	-	-	-	-	-	1	-	-	-	-	-	-	-	1	-	1	1
hepatocyte vacuolation	-	-	-	2	-	-	-	-	-	-	2	-	-	-	-	-	-	-
cytoplasmic rarefaction, hepatocytes	1	1	-	-	-	-	-	-	2	-	1	2	-	1	-	-	-	-
hepatocyte hypertrophy	-	-	-	-	-	1	-	1	-	1	-	-	-	-	-	-	-	-
inclusions, hepatocytes	-	-	-	-	-	-	-	1	-	-	-	-	-	-	-	-	-	-
hepatocyte degeneration and necrosis	-	-	1	-	-	-	-	-	1	-	1	-	-	-	1	-	-	-
**Kidney**																		
infiltration mononuclear cells	1	-	1	1	-	-	1	1	1	1	-	1	1	-	-	-	-	-
tubular basophilia	-	-	1	1	-	2	1	2	1	2	1	-	-	1	1	-	-	-
tubular proteinuria	-	-	-	-	3	2	-	1	-	-	1	-	-	1	-	1	-	1
tubular hydropic degeneration	-	-	-	-	3	-	-	-	-	-	-	-	-	-	-	-	-	-
tubular pigment	-	-	-	-	-	-	-	-	-	1	-	-	-	-	-	-	-	-
tubular swelling and dilatation	-	-	-	-	-	-	-	-	-	-	-	-	-	-	-	1	1	1

1: Minimal; 2: Mild; 3: Moderate; 4: Marked

Supplementary table 5. Histological observations in HSA-LR mice after Pip6a-PMO treatment. The toxicology analysis of Pip6a-PMO-CAG7 (Pip6a-PMO) multiple injection treatments in HSA-LR mice indicated the existence of liver changes involving dose dependent hypertrophy and intranuclear inclusions. These changes are present in animals from the 2 injections / 2 weeks and 3 injections / 2 weeks groups but not in the 3 injections / 4 weeks or 3 injections / 6 months groups, suggesting complete recovery. The most significant treatment-related kidney changes included tubular proteinuria and dose dependent tubular basophilia and tubular hydropic degeneration. The reduced incidence and severity of the latter two changes in the 3 injections / 4 weeks and 3 injections / 6 months group suggest partial recovery.

Supplementary table 6. Primers and probes used for PCR, qPCR and FISH/smFISH analysis.

	Transcript	Forward (5' - 3')	Reverse (5' - 3')
**Splicing**	Mouse		
	Clcn1	TTCACATCGCCAGCATCTGTGC	CACGGAACACAAAGGCACTGAATGT
	Mbnl1	GCTGCCCAATACCAGGTCAAC	TGGTGGGAGAAATGCTGTATGC
	Atp2a1	GCTCATGGTCCTCAAGATCTCAC	GGGTCAGTGCCTCAGCTTTG
	Human		
	LDB3	GCAAGACCCTGATGAAGAAGCTC	GACAGAAGGCCGGATGCTG
	MBNL1	GCTGCCCAATACCAGGTCAAC	TGGTGGGAGAAATGCTGTATGC
	SOS1	CAGTACCACAGATGTTTGCAGTG	TCTGGTCGTCTTCGTGGAGGAA
	DMD	TTAGAGGAGGTGATGGAGCA	GATACTAAGGACTCCATCGC
**Expression**	*HSA*	TTCCATCGTCCACCGCAAAT	AGTTTACGATGGCAGCAACG
	Internal probe	FAM-AGACACACTCCACCTCCAGCACGCGACTT-TAMRA	
**FISH and smFISH**	CUG repeats	Cy3-CAGCAGCAGCAGCAGCAGCAG	
		Cy5-CAGCAGCAGCAGCAGCAGCAG	
	hDMPK	TCCGGGGGACACAGCAACCGCTGAATTTACACTCGGACCTCGTCGACATGCATT	
		ACAGGTAGTTCTCATCCTGGAAGGCGAAGTTTACACTCGGACCTCGTCGACATGCATT	
		CAGTCGGACCTCCTTAAGCCTCACCATTACACTCGGACCTCGTCGACATGCATT	
		CAACTGCCGGACGTGTGCCTCTAGGTTTACACTCGGACCTCGTCGACATGCATT	
		TTCTGGTTGTCCGTGCGGATGGCCTCTTACACTCGGACCTCGTCGACATGCATT	
		CGCTGGAACTGCCACTTCAGCTGTTTTTACACTCGGACCTCGTCGACATGCATT	
		CTGTCCCTGAGGGCCATGCAGGAGTATTACACTCGGACCTCGTCGACATGCATT	
		GTGAGCCCGTCCTCCACCAAGTCGAATTACACTCGGACCTCGTCGACATGCATT	
		TGCATGTGTCGGTGGCACCTTCGAAATCTTACACTCGGACCTCGTCGACATGCATT	
		GCTCCTTGTAGTGGACGATCTTGCCATTTACACTCGGACCTCGTCGACATGCATT	
		GCGTCTGCCCATAGAACATTTCATAGGCTTACACTCGGACCTCGTCGACATGCATT	
		CATCTGCCCGCAGCTTGAGGCAAGAGTTACACTCGGACCTCGTCGACATGCATT	
		GCCACAGCGGTCCAGCAGGATGTTGTTTACACTCGGACCTCGTCGACATGCATT	
		GGTTTGATGTCCCTGTGCACGTAGCCATTACACTCGGACCTCGTCGACATGCATT	
		CCGCCCACGTAATACTCCATGACCAGTTACACTCGGACCTCGTCGACATGCATT	
		CGTCTGCTTCATCTTCACTACCGCTATTACACTCGGACCTCGTCGACATGCATT	
		GCCCACTGCAAGAAGTCGGCCACGTATTACACTCGGACCTCGTCGACATGCATT	
		CGCACCTTCCCGAATGTCCGACAGTGTTACACTCGGACCTCGTCGACATGCATT	
		GTCTCCGCCGTGGAATCCGCGTAGAATTACACTCGGACCTCGTCGACATGCATT	
		CTCCCCAAACTTGCTCAGCAGTGTCATTACACTCGGACCTCGTCGACATGCATT	
		CCCTCTTCAGCATGTCCCACTTGTTCTTACACTCGGACCTCGTCGACATGCATT	
		CCCGCGTCCGATCACCTTCAGAATCTTTACACTCGGACCTCGTCGACATGCATT	
		ACCATCCCAGTCGAGGCCAAAGAAGAAGGTTACACTCGGACCTCGTCGACATGCATT	
		AGTAGCCCACAAAAGGCAGGTGGACCCTTACACTCGGACCTCGTCGACATGCATT	
	FLAP	Cy3-AATGCATGTCGACGAGGTCCGAGTGTAA-Cy3	
